# Three-way interaction model to trace the mechanisms involved in Alzheimer’s disease transgenic mice

**DOI:** 10.1371/journal.pone.0184697

**Published:** 2017-09-21

**Authors:** Nasibeh Khayer, Sayed-Amir Marashi, Mehdi Mirzaie, Fatemeh Goshadrou

**Affiliations:** 1 Department of Basic Sciences, Faculty of Paramedical Sciences, Shahid Beheshti University of Medical Sciences, Tehran, Iran; 2 Department of Biotechnology, College of Science, University of Tehran, Tehran, Iran; 3 School of Biological Sciences, Institute for Research in Fundamental Sciences (IPM), Tehran, Iran; 4 Department of Applied Mathematics, Faculty of Mathematical Sciences, Tarbiat Modares University, Tehran, Iran; King Abdullah University of Science and Technology, SAUDI ARABIA

## Abstract

Alzheimer's disease (AD) is the most common cause for dementia in human. Currently, more than 46 million people in the world suffer from AD and it is estimated that by 2050 this number increases to more than 131 million. AD is considered as a complex disease. Therefore, understanding the mechanism of AD is a universal challenge. Nowadays, a huge number of disease-related high-throughput “omics” datasets are freely available. Such datasets contain valuable information about disease-related pathways and their corresponding gene interactions. In the present work, a three-way interaction model is used as a novel approach to understand AD-related mechanisms. This model can trace the dynamic nature of co-expression relationship between two genes by introducing their link to a third gene. Apparently, such relationships cannot be traced by the classical two-way interaction model. Liquid association method was applied to capture the statistically significant triplets which are involved in three-way interaction. Subsequently, gene set enrichment analysis (GSEA) and gene regulatory network (GRN) inference were applied to analyze the biological relevance of the statistically significant triplets. The results of this study suggest that the innate immunity processes are important in AD. Specifically, our results suggest that *H2-Ob* as the switching gene and the gene pair {*Csf1r*, *Milr1*} form a statistically significant and biologically relevant triplet, which may play an important role in AD. We propose that the homeostasis-related link between mast cells and microglia is presumably controlled with *H2-Ob* expression levels as a switching gene.

## Introduction

Alzheimer's disease (AD) is the most common cause for dementia in human. AD often affects individuals over the age of 60, with increasing risk by age. Based on the last report of the AD International, more than 46 million people in the world suffer from AD and it is estimated that by 2050 this number increases to more than 131 million [1]. AD is associated with a complex progression of neurodegeneration that results in memory impairment and loss of other cognitive processes, as well as the presence of non-cognitive symptoms including delusions, agitation and changes in behavior and personality [2]. Although the causes of AD and its progress are not well known yet, it is characterized by the presence of amyloid plaques and neurofibrillary tangles outside and inside the neuronal cells, respectively. Oxidative stress, chronic inflammation, gliosis and excitotoxicity are other pathologic symptoms of this disease. Reduction in neuronal synapses following the cellular death in hippocampus area of brain, can be the reason for loss of spatial memory and anomaly in patients’ behavior [3].

AD pathogenicity procedure is complex. Without a proper understanding of the pathogenicity mechanisms, treatments will only lead to decreasing the disease symptoms and attacks, while the main therapy is not yet available. Therefore, finding pathogenicity mechanisms is a universal concern [4,5].

High-throughput data produced under different biological conditions, includes a large amount of information about gene regulation processes and protein functions. In addition, depending on the applied computational method, different conclusions can be drawn from the same biological data [6–10].

A common way for analyzing functionally-related genes is the use of two-way interaction model, in which expression level of two genes are measured according to their pairwise correlation value [11,12]. Naturally, genes with high correlation tend to be functionally-related. However, because of various reasons, some functionally-related gene pairs may not show a significant correlation. Therefore, such a gene pair may not be discovered with a two-way interaction model. For example, the co-expression relation of two functionally-related genes may change based on the cellular state [13]. Furthermore, some proteins have multiple molecular roles, and therefore, such proteins can be involved in several biological procedures [13]. In particular, some co-expression relations can only be seen in disease condition, but not in healthy tissues [14,15]. Thus, the two-way interaction model may be too simplistic to explain some of the complex molecular relations [16].

In a few previous studies, it had been suggested that classification can help in analyzing the gene expression patterns when the relationship between gene expression values is not monotonic. Aid-Pavlidis and coworkers (2009) traced those genes whose co-expression with BDNF gene is preserved under different conditions, e.g., age, gender and disease state. Dysregulation of this gene is observed in AD, Huntington’s disease and Parkinson's patients. In another work, Wang and coworkers (2015) introduced a method for estimating the strength of gene interactions in groups. Their method infers the strength of gene group interactions using sparse canonical correlation analysis coupled with repeated random partitioning and subsampling of the gene expression data. More specifically, to select the strength of gene group interaction, the co-expression relationship between genes is traced by considering different subsets of genes, and then, determined the conditional dependencies between genes after removing the influences of a set of other functionally related genes.

**Fig 1:**
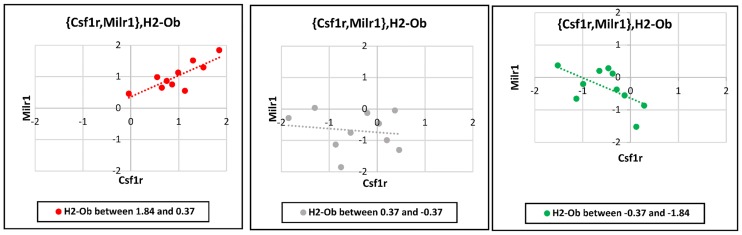
An example of three-way interaction. In a three-way interaction with switching mechanism the correlation between two genes, namely X_1_ and X_2_ is considered. Then, it is assumed that there is a third gene, namely the "switching gene" denoted by X_3_, whose expression level affects the co-expression relationship of the two other genes. In other words, based on the expression levels of the third gene (X_3_), the expression levels of the two other genes ({X_1_, X_2_}) are either directly or inversely correlated. Here, the three-way interaction with switching mechanism between *H2-Ob* (as the switching gene) and {*Csf1r*, *Milr1*} (as {X_1_, X_2_}) is shown. (A) When *H2-Ob* gene is up-regulated (i.e., its normalized expression level is between 0.37 and 1.84), there is a direct correlation between *Milr1* and *Csf1r* expression levels (red); (B) When *H2-Ob* gene is in the intermediate state (i.e., its normalized expression level is between 0.37 and -0.37), expression levels of *Milr1* and *Csf1r* are not correlated (grey); (C) When *H2-Ob* gene is down-regulated (normalized expression level of it is between -1.84 and -0.37), there is an inverse correlation between *Milr1* and *Csf1r* expression levels (green). This triplet will be further explained in the Discussion section.

In the present study, a three-way interaction model is used for determining the disease-related mechanisms in AD. Such a model can trace the dynamic nature of co-expression relationship of two genes ({X_1_, X_2_}) by the introducing a third gene (X_3_), whose expression level modulates the correlation between X_1_ and X_2_. Fig 1 illustrates an example of a three-way interaction. One of the statistical methods for this purpose is liquid association analysis. This method describes co-expression changes of two genes based on the expression level of a third gene, which is sometimes referred to as the controller gene [13].

## Materials and methods

### Gene expression

The dataset includes gene expression of the cerebral cortex of APP/PS1 and WT mouse littermates (accession number E-MTAB-2121 [17] in ArrayExpress database). The data were generated using the Affymetrix microarray platform and the GeneChip Mouse Gene 1.1 ST Array. The raw data were preprocessed and normalized using robust multi-array analysis method (implemented in the Bioconductor Limma package [18]).

One of the main challenges in microarray data analysis is the large dimensionality. To reduce the data size, duplicate probes were removed using genefilter function in the Bioconductor genefilter package [19]. It should be noted that this function retain the highest interquartile range (IQR) probe for each gene.

### Determining three-way interactions

#### Selection of candidate switching genes

In this study, a candidate switching gene is defined to have a significantly different gene expression level in diseased and normal samples. Therefore, empirical Bayes t-test was performed using the Imfit function in the Limma package [18] for each gene, given the groups of arrays. Afterward, false discovery rate estimate (FDR) was calculated using the Benjamini-Hochberg (BH) correction method [20]. Differentially-expressed genes with FDR <0.05 were selected as candidate switching genes.

#### Liquid association triplets

Three-way interactions between candidate switching genes and all possible pairwise combinations of genes in the dataset were calculated using fastMLA function in fast LA package [21]. This package uses a modified liquid association algorithm for determining changes in co-expression relations of a gene pair, X_1_ and X_2_, based on the expression level of a third gene (X_3_).

In the fast liquid association algorithm, for each gene triplet, an MLA score is computed. More specifically, MLA (X_1_, X_2_ |X_3_) can be estimated as:

$$\overset{\wedge }{MLA}=\frac{\overset{M}{\underset{i}{\Sigma }}{\hat{\rho }}_{i}\bar{X_{3i}}}{M}$$

where *M* is the number of bins over X_3_, ${\hat{\rho }}_{i}$ is the Pearson’s correlation coefficient of X_1_ and X_2_ in samples of the *i'*th bin, and $\bar{X_{3i}}$ is the mean of expression values of X_3_ in the *i*'th bin.

Performing two preprocessing steps on the data is necessary before running fastLA.

1. To obtain asymmetric edges for each variable, normal quintile transformation should be performed for each gene, based on the approach proposed by Li [13].
2. Expression level of each gene should be standardized to zero mean and standard deviation of one [22].

The first preprocessing was performed using an in-house implementation, while the second one by using CTT package[23].

False discovery rate (FDR) was estimated by using the Benjamini-Hochberg correction method and liquid association triplets with FDR < 0.001 was chosen as statistically significant triplets.

### Gene set enrichment analysis

Gene set enrichment analysis (GSEA) is a statistical method to assess the significance of the shared association of several genes (proteins) using predefined annotations [24]. For every switching gene, and also for all of the genes involved in all statistically significant triplets, GSEA was separately performed based on biological process and cellular component using the gene ontology (GO) database. Furthermore, the same analyses were performed to find enriched pathways in KEGG database [25]. For the above-mentioned analyses, we used ClueGO tool [26] (with a Kappa threshold of 0.4) within the Cytoscape v.3.3.0 environment [27]. Right-sided hypergeometric test and the Benjamini-Hochberg correction method were used for validation of enrichment analysis.

### Construction of gene regulatory network

A gene regulatory network (GRN) is a framework to model complex regulatory mechanisms that control gene expression in cells. A GRN is represented as a directed graph, which consists of nodes (genes) and directed edges (regulatory relations) that can be activatory or inhibitory. By using such a network, changes in gene expression can be predicted after a particular stimulation [28]. Here, we used ARACNE (Algorithm for the Reconstruction of Accurate Cellular Networks) [29] for constructing the GRN. ARACNE is an approach for reverse engineering of cellular networks from gene expression data. This algorithm infers directed regulatory interactions between each transcriptional regulator and its potential targets based on mutual information. Construction of GRN was performed using geWorkbench_2.6.0 software application ARACNE between switching candidate genes as hub markers and all of the genes involved in the statistically significant triplets by considering *p*-value <0.0001.

The code for above analyses is available from: https://github.com/NKhayer/3wayintraction

## Results

### Preprocessing and normalization of gene expression data

After preprocessing and normalization using robust multi-array analysis method, the final dataset consisted of 30 samples including 15 wild type and 15 APP/PS1 transgenic samples (It should be noted that two arrays (i.e., 15.w.3 and 26.w.12) were discarded from dataset due to low quality). In the original dataset, each sample included 35556 probes. As a result of removing duplicated probes, this number dropped 20527 probes.

Additional data including array images, MA plots, box plots and data histograms before and after of normalization are provided in S1 Fig.

### Selection of candidate switching genes

In order to determine the candidate switching genes, eBayes t-test was performed on the normalized data. We selected 38 differentially-expressed genes by considering FDR <0.05 as the candidate switching genes. The detailed results of this analysis are available in S1 Table.

### Determining significant three-way interactions

**Fig 2:**
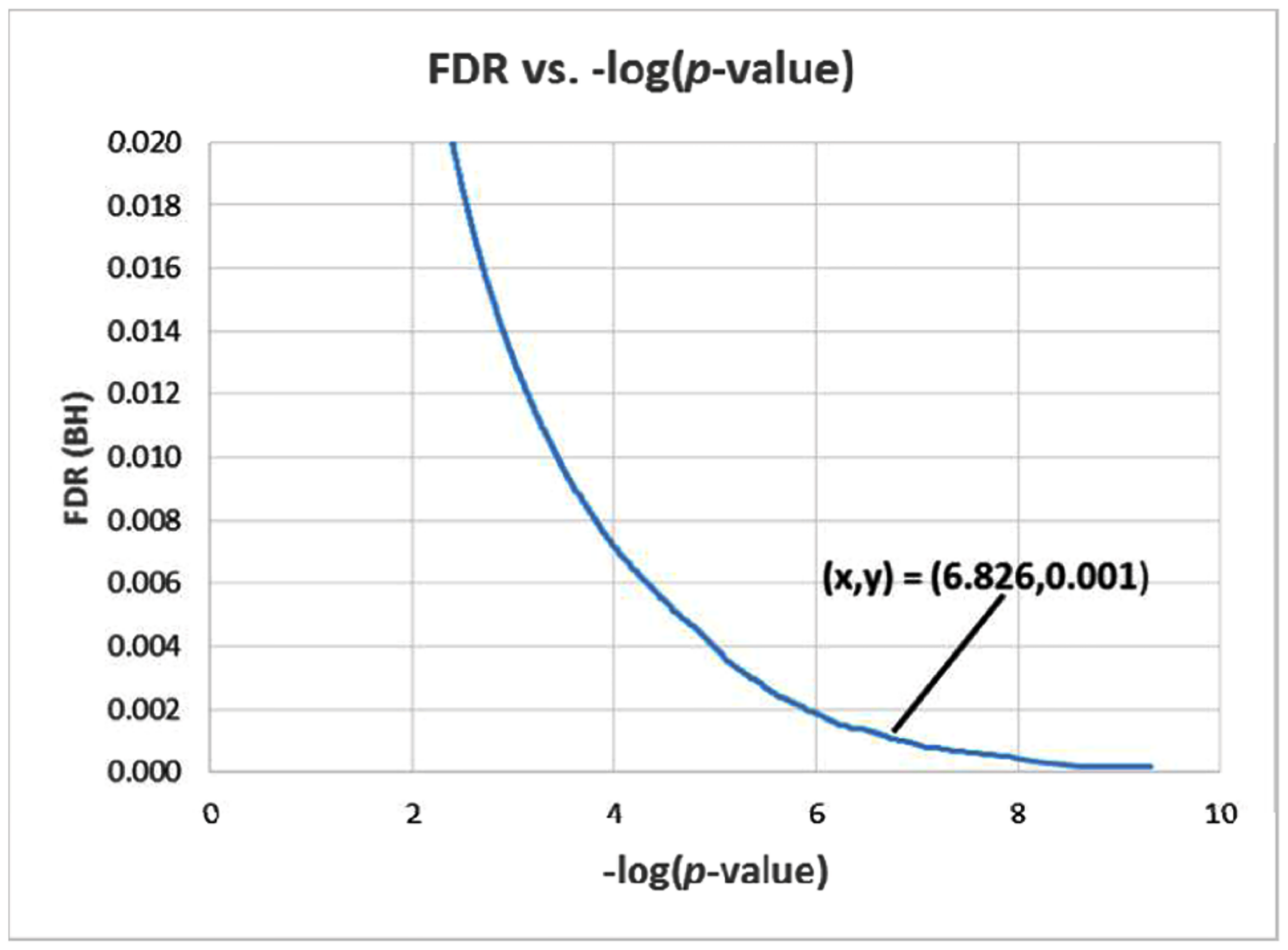
FDR vs. -log (*p*-value). The changes in FDR (BH-corrected *p*-value) versus -log (*p*-value) for the first 300000 results of fastLA [21]. As shown FDR = 0.001 corresponds to -log (*p*- value) = 6.817.

Using fastLA package, liquid association analysis was performed for every combination of a candidate switching gene (X_3_) and every possible pair of genes in the dataset {X_1_, X_2_}. The top 300000 triplets with the highest significance levels based on *p*-value were defined as outputs of this analysis. In S2 Fig, *p*-value histogram of these three-way interactions is available. Fig 2 shows the changes in FDR using Benjamini-Hochberg method versus -log (*p*-value) for the first 300000 triplets. For the rest of our analysis, the set of all three-way interactions were chosen by considering FDR <0.05, which consists of 106 triple combinations. Within these triplets, we found 241 unique genes including, 35 unique X_3_ genes and 207 unique X_1_ or X_2_ genes. These results show that repetition of X_1_ and X_2_ in triplets is rare. The list of all statistically significant triplets are presented in S2 Table. To show the switching genes which are involved in the numerous significant triplets, a network from statistically significant triplets was constructed (see S3 Fig). This network shows that *Slc14a1*, *Dock8*, *Slc11a1* and *Parvg* (with 10, 7, 7 and 6 connections, respectively) have the largest contribution in the statistically significant triplets rather than other X_3_. Also the network expansion shows that the switching mechanisms are not limited to specific number of X_3_, as expected for complex diseases such as AD.

#### Results of gene set enrichment analysis

**Fig 3:**
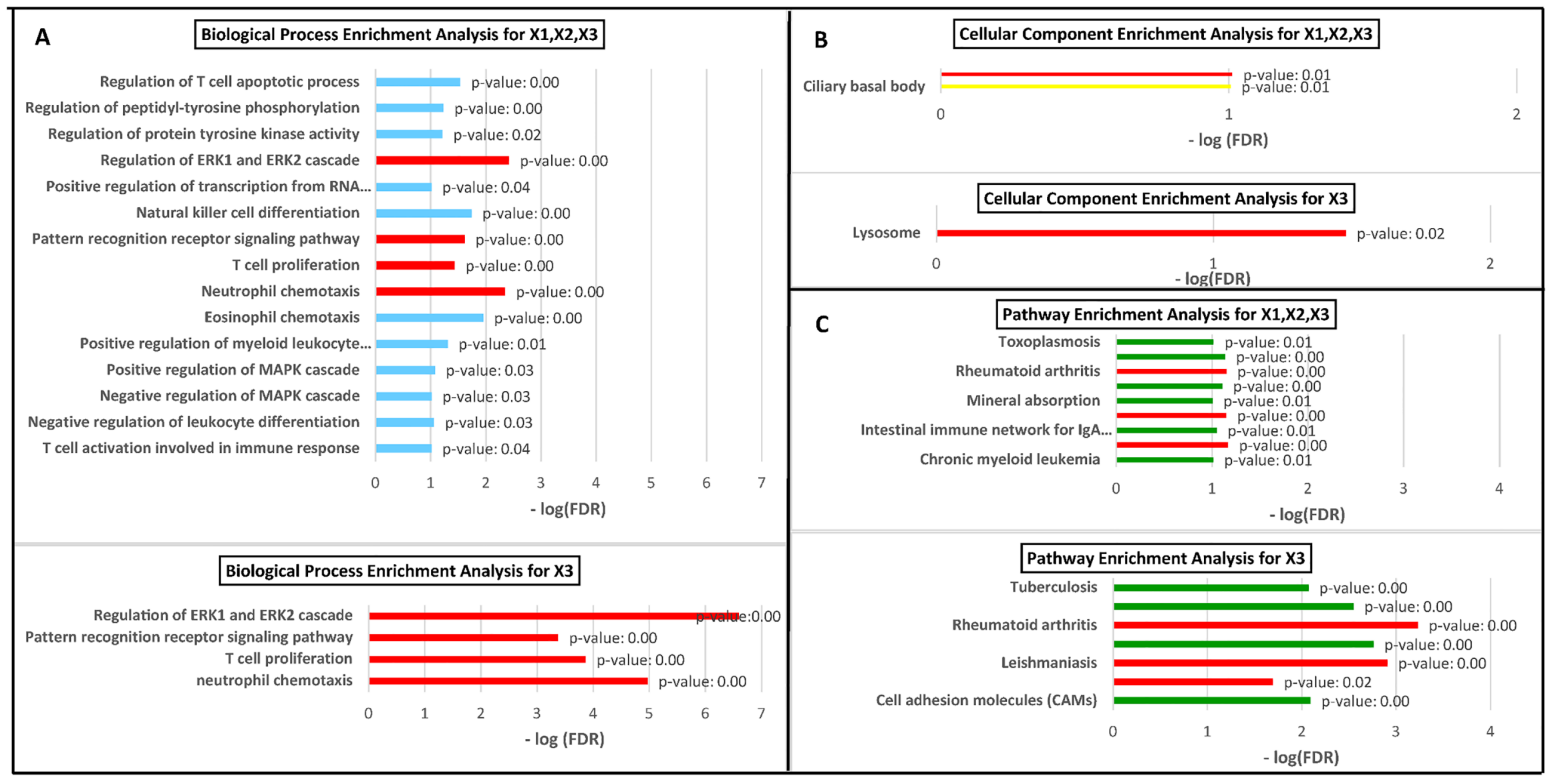
Gene set enrichment analysis. Enriched terms based on (A) “biological process”; (B) “cellular component”; and (C) “KEGG pathway” for two gene groups, genes in X_3_ position and all of the genes involved in the triplets. The common terms in these two groups are shown in red. The high frequency of common terms suggest that the results of liquid association method are consistent with the biological expectation from three-way interactions, that is, the presence of switching and switched genes in the same biological pathway.

We used GSEA in order to find biologically-relevant triplets in the 106 statistically significant triplets. GSEA was performed with *p*-value <0.05 and FDR <0.1 for X_3_ genes (35 individual genes) and all of the involved genes in triplets (241 individual genes). Enriched terms based on “biological process”, “cellular component” and “KEGG pathway” are reported in Fig 3. Since the terms in lower levels of gene ontology are more general, enriched terms for “biological process” and “cellular component” in levels lower than level 10 and level 9 are not reported. The results of GSEA for two gene groups, i.e., genes in X_3_ position and all of the involved genes in triplets, show that there are a large number of common terms in two groups. For verifying that the large number of common terms in group 1 (i.e., X_3_) and group 2 (i.e., X_1_, X_2_, X_3_) are significantly different from random, we extracted 30 random subsets from group 2, each subsets including 35 genes. After gene set enrichment analysis, very rare overlap was observed between group 2 and each of the random subsets. These results confirm that the large number of common terms in group 1 and 2 are not by chance. The results of this analysis are available in S3 Table.

The terms, "Regulation of ERK1 and ERK2 cascade", "Pattern recognition receptor signaling pathway", "T-cell proliferation", and "Neutrophil chemotaxis", based on biological process (Fig 3A) and the term "Lysosome" based on cellular component (Fig 3B) and a number of immunity-related terms, including "Cytokine-cytokine receptor interaction", "Rheumatoid Arthritis", and "Leishmaniasis" based on biological pathway (Fig 3C) are common in two groups. The complete list of enriched terms is available in S4 Table. Furthermore, the categorized biological processes based on the AD-related pathways are available in S5 Table.

**Fig 4:**
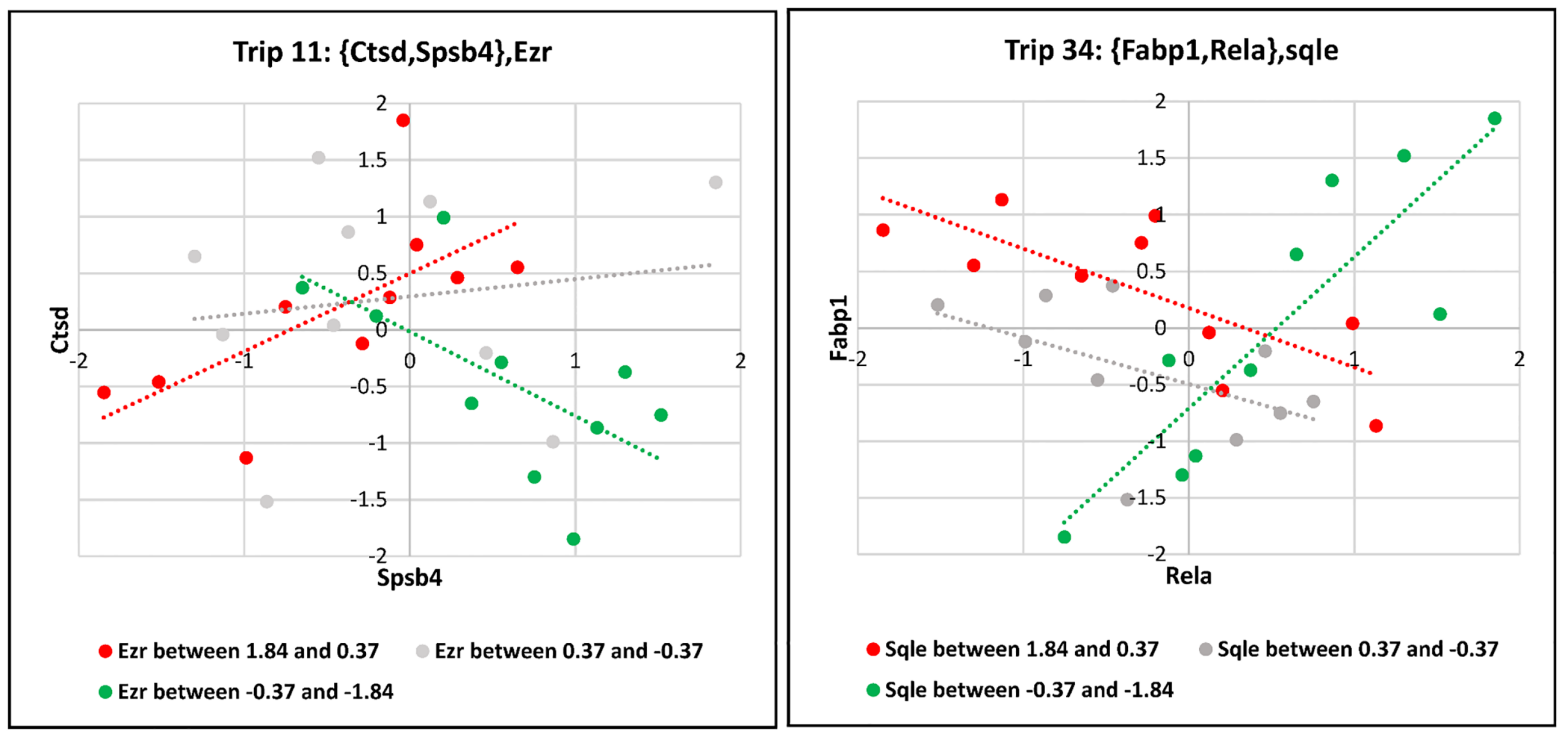
Examples of the statistically triplets. In each case, a considerable change in the correlation of X_1_ and X_2_ occurs as a result of change in X_3_.

Based on the proposed definition of three-way interactions of switching mechanism model, it is expected that in biologically-relevant triplets, X_1_ and X_2_ are in the same pathway or biological process. By tracing triplets in the enriched terms, 12 triplets in which X_1_ and X_2_ are involved in the same biological process or pathway were determined (Table 1). As it are reported in Table 1, regulatory and innate immunity processes are frequently repeated in these triplets. Fig 4 indicates two exemplary scatter plots of these triplets in three different ranges of associated X_3_ expression level which shows a considerable change in the correlation of X_1_ and X_2_ as a result of change in X_3_. Scatter plots of all of the 12 triplets are available in S4 Fig.

**Table 1:** Biologically relevant triplets. By tracing statistically significant triplets in the enriched terms, 12 triplets in which X_1_ and X_2_ are involved in the same biological process or pathway were determined.

	GO Term	GO Levels	*p*-value	FDR (BH)	Triplet Number
**Biological Process**	Regulation of immune system process	[2, 3]	1.13E-05	5.20E-03	34,72,90
	Defense response	[3]	2.15E-04	6.20E-03	34
	Response to other organism	[2, 4]	3.32E-04	9.01E-03	34
	Regulation of defense response	[4, 5]	9.20E-04	1.21E-02	34
	Cytokine production	[3]	7.46E-04	1.23E-02	78
	Negative regulation of apoptotic signaling pathway	[4, 5, 6, 7, 8]	1.24E-03	1.50E-02	22
	Regulation of response to stimulus	[2, 3]	1.28E-03	1.52E-02	22,29,34
	Positive regulation of cellular metabolic process	[3, 4, 5]	1.46E-03	1.64E-02	34,45
	Cellular response to chemical stimulus	[3]	1.91E-03	1.80E-02	34,38,22
	Regulation of response to external stimulus	[3, 4]	1.76E-03	1.81E-02	34
	Cellular response to hydrogen peroxide	[5, 6]	2.89E-03	2.30E-02	34
	Programmed cell death	[4]	3.27E-03	2.48E-02	34,22
	Negative regulation of apoptotic process	[5, 6, 7]	5.06E-03	3.16E-02	22,34
	Regulation of apoptotic signaling pathway	[4, 5, 6, 7]	5.58E-03	3.30E-02	22
	Positive regulation of biological process	[1, 2, 3]	7.43E-03	3.90E-02	34,45,60,90
	Positive regulation of metabolic process	[2, 3, 4]	8.37E-03	4.16E-02	34,45
	Positive regulation of macromolecule metabolic process	[3, 4, 5]	8.60E-03	4.23E-02	45
	Cellular response to oxygen-containing compound	[4]	9.06E-03	4.27E-02	34
	Cell surface receptor signaling pathway	[4, 5]	1.00E-02	4.64E-02	46
	Positive regulation of cellular process	[2, 3, 4]	1.15E-02	5.18E-02	34,45,90
	Regulation of apoptotic process	[5, 6]	1.35E-02	5.68E-02	22,34
	Oxidation-reduction process	[3]	1.53E-02	5.94E-02	38
	Negative regulation of cell proliferation	[3, 4, 5]	1.50E-02	5.98E-02	88
	Response to wounding	[3]	1.59E-02	6.11E-02	60
	Apoptotic signaling pathway	[4, 5, 6]	1.61E-02	6.11E-02	22
	Regulation of protein metabolic process	[4, 5]	1.82E-02	6.66E-02	34
	Regulation of response to stress	[3, 4]	2.04E-02	7.09E-02	34
	Regulation of signal transduction	[3, 4, 5]	2.52E-02	8.01E-02	22
	Response to oxygen-containing compound	[3]	2.67E-02	8.34E-02	34
	Single-organism transport	[3, 4]	3.02E-02	8.83E-02	72
	Transport	[3]	3.34E-02	9.24E-02	72
**Pathway**	Immune System		9.80E-04	3.82E-02	11,90
	Adaptive Immune System		8.02E-04	4.69E-02	11

#### Determining the position of triplets in gene regulatory network

**Fig 5:**
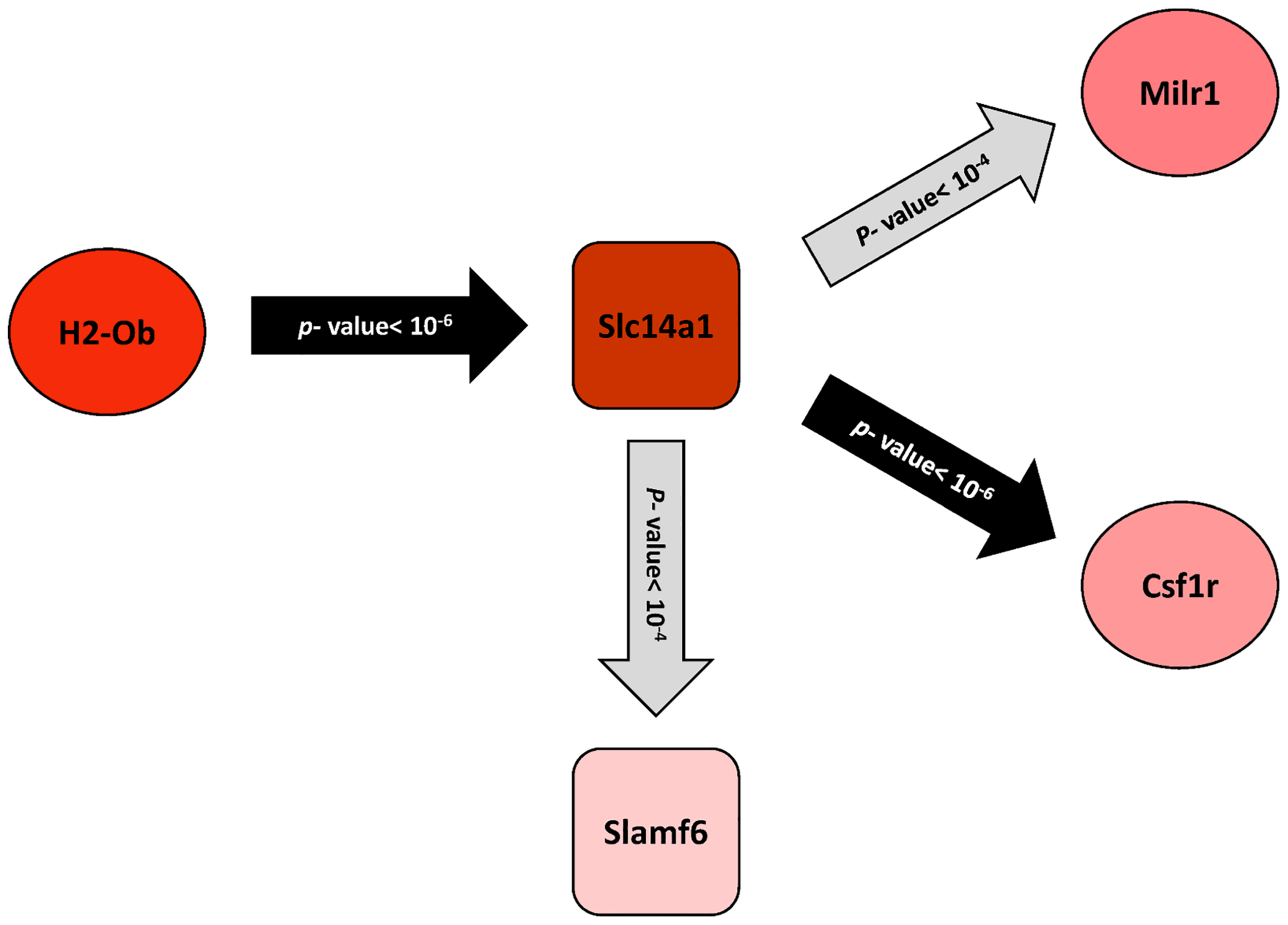
Regulatory relationships within triplets. The regulatory relationships of significant triplets obtained from liquid association were traced in the GRN. The more the intensity of the red color, the more the up-regulation of the gene in Alzheimer’ disease. As shown there are regulatory relationships between *Milr1*, *Csf1r* and *H2-Ob* in triplet 72. Additionally, regulatory relationships are observed between *Slc14a1* and *Slamf6* in the triplet 97.

As another attempt for analyzing the functional relevance of three-way interactions, we reconstructed a GRN based on ARACNE. By considering *p*-value <0.0001 as the threshold, the resulting network included 42 nodes and 82 edges. This network is available S1 File, Section A. The regulatory relationship of significant triplets obtained from liquid association method was traced in this network and the results are shown as a subnetwork in Fig 5. As it is observed, the regulatory relationship between X_3_ from 72^nd^ triplet (*H2-Ob*) and two other genes in this triplet (*Milr1* and *Csf1r*) can be seen with *Slc14a1* as an intermediate. In addition, a direct regulatory interaction between X_3_ (*Slc14a1*) and X_2_ (*Slamf6*) from 97^th^ triplet is observed.

The details of construction GRN and detection of significant triplets in this network is available in S1 File, Section B.

## Discussion

Although pairwise gene interactions have been widely investigated in AD [30–32], in the present study, for the first time, we investigated the switching mechanism in AD using a three-way interactions model. In two-way interaction model, the co-expression level of two genes, X_1_ and X_2_, is measured based on their correlation coefficient. As a result, highly correlated genes are identified as functionally related and their encoded proteins are assumed to be involved in the same biological pathway, to participate in the same structural complex or to be regulated by the same mechanism. Nevertheless, some of the functionally related genes may not be co-expressed. A reason for this fact is that gene expression is often sensitive to changes in cellular states such as the presence or absence of the hormones and metabolites or ionic homeostasis [33]. Since biological pathways are interwoven, and each protein may have several cellular roles, co-expression of all of the functionally-related genes might change depending on cellular states, which is often not well-known [13]. Therefore, degree and pattern of correlation of two gene expression profiles can be affected by changes in internal cellular states. In other words, if the condition changes, two functionally-related genes with direct correlation may become uncorrelated or even inversely correlated. Hence, two-way interaction model might be too simplistic to explain complex molecular relations. In contrast, three-way interaction model, which can capture more complicated behavior compared to the two-way interaction model, might be able to demystify more complex molecular relations [16].

The main difference of Aid-Pavlidis et al.’s study with the three-way interaction studies is that the former aims to trace the co-expression relations within a gene expression dataset, while three-way interaction studies trace differential co-expression relations. The common assertion in Wang et al.’s study and the three-way interaction studies is that the co-expression relationship between genes may be influenced by some other genes.

Three common methods exist for finding three-way interactions with switching mechanism: liquid association [13], differential correlation coefficient [34], and interaction test [35]. Here, we used fast Liquid Association (fastLA) algorithm [21]. The reason behind this selection is that liquid association is applicable to X_3_ with quantitative data such as gene expression data, while interaction test and differential correlation coefficient methods are only used for X_3_ with qualitative data such as genotype data or different classes of gene expression data. In addition, in liquid association method, the high computational load, which is the main challenge in investigating the genome-wide three-way interactions, is decreased. Therefore, this algorithm can be practically applied to high-throughput data like mouse microarray data.

The E-MTAB-2121 dataset includes gene expression data of APP/PS1 and WT mouse littermates. In S1 Text, Section A, we discussed about reasons for choosing the mouse rather than human dataset.

The low repetition at X_1_ and X_2_ genes in the obtained triplets from fastLA approves the critical role of X_3_ in the change of {X_1_, X_2_} expression correlation, because in the absence of X_3_ it is expected that in every run of fastLA the same {X_1_, X_2_} are reported as the significant switched gene pairs.

From the set of 35 “X_3_” genes, only 6 genes are reported in the Lopez-Gonzalez et al.’s study as the differentially expressed genes. In S1 Text, Section B, we discussed about this small similarity.

A high frequency of common terms is observed between the GSEA results of the two gene groups, i.e., the set of X_3_ genes and the set of all genes in the triplets (Fig 3A). This consistency suggests that the switching genes and the switched genes are associated to biologically related pathways. In addition, previous studies showed the importance of these common enriched terms in AD (see below) which confirm the validity of the results obtained in this study.

### Biological processes enrichment analysis

The common enriched “biological process” terms between both of the gene groups (i.e., genes in X_3_ position and all of the genes involved in triplets) are "Regulation of ERK1 and ERK2 cascade", "Pattern recognition receptor signaling pathway", "T-cell proliferation", and "Neutrophil chemotaxis" (Fig 3A). All of the four common enriched terms are also biologically relevant to AD. See below.

Up-regulation of ERK1/2 is associated to hyper-phosphorylation of tau protein, and consequently, production of neurofibrillary tangles [36,37]. This kinase is suggested as drug target for AD [38]. Pattern recognition receptors are the intermediate proteins to trigger the innate immune system in AD [39–41]. T cell proliferation is induced by interleukin 15, which is produced following the activation of macrophages in AD [42]. Furthermore, monocyte-derived dendritic cells in AD patients are able to interfere in induction of T cell proliferation [43]. Neutrophil chemotaxis is induced by β-amyloid peptides in AD [44, 45].

### KEGG pathways enrichment analysis

The three KEGG pathways that are commonly enriched in both groups are "Cytokine-cytokine receptor interaction", "Rheumatoid Arthritis", and "Leishmaniasis" (Fig 3C). The importance of cytokine/cytokine receptor interaction in AD is reported previously [44,45]. Both rheumatoid arthritis and AD are known to be associated with inflammation [46–48]. Moreover, studies such as Refs. [49–51] have reported the relationship between the two diseases. The common aspect of AD and Leishmaniasis is the activation of innate immune system. Activation of innate immune system in Leishmaniasis and AD is mediated through glycocalyx on the surface of Leishmania parasite [52] and Aβ Aggregates [53], respectively. Interestingly, glycogen synthase kinase-3 inhibitors, which are introduced as anti-parasite drugs for treating leishmaniasis in the first place, are also suggested as candidates for AD treatment [54,55].

### Cellular components enrichment analysis

"Lysosome" is the common enriched cellular component term in two group of genes (Fig 3B). Mutation of proteins involved in lysosomal/endosomal transports are identified in many neurological diseases and dysfunction of lysosomal system is a definite characteristic of AD [56–58]. Based on these facts, one can explain why lysosome has a significant contribution in the onset and progression of AD.

More particularly, the 12 triplets in which X_1_ and X_2_ genes are involved in the same pathway or biological process (Table 1) show that regulatory processes and innate immunity are important in the switching mechanisms involved in AD. According to the concept of switching mechanism, the presence of triplets in regulatory pathways is expectable.

The 72^nd^ triplet, which consists of genes *H2-Ob* and {*Csf1r*, *Milr1*}, is one of the most statistically significant triplets in our analysis. Furthermore, this triplet is biologically relevant, based on both gene set enrichment analysis (Table 1) and gene regulatory network analysis (Fig 5). As it is shown in Table 1, genes in this triplet are involved in regulatory of immune system processes. Innate immune system has a prominent role in the onset and progression of AD. Neuropathology of AD indicates a strong innate immunity response which is characterized by activated microglia [59], de novo expression or increase of various macrophage antigens [60], and inflammatory cytokine production [61]. Proliferation and activation of microglial cells is a noticeable feature of several neurodegenerative disorders such as AD[62]. This mechanism is regulated by activation of *Csf1r* (colony-stimulating factor1 receptor) [62]. Up-regulation of *Csf1r* is reported both in amyloid model of mouse [63] and post-mortem human samples [62]. Olmos-Alonso et al., suggest *Csf1r* gene as a drug target for prevention of AD progression and show that *Csf1r* inhibition in transgenic mice APP/PS1 using GW2580, a tyrosine kinase inhibitor, results in blockage of microglia proliferation, change of microglia inflammatory profile to non-inflammatory phenotype and also improvement in memory and behavioral tasks, and prevention of synapse destruction [64]. Spangenberg et al., showed that *Csf1r* inhibition in 5xfAD AD mouse model leads to clearance of active chronic microglia. This clearance not only is not harmful in the animal model, but also results in improvement of synapse disorders compared to the disease state and additionally lead to decrease in neuronal inflammation and prevents neuron destructions [65].

The protein encoded by *Milr1* gene (mast cell immunoglobulin-like receptor 1, equivalent to allergy inhibitory receptor 1), is an immunoglobulin-like receptor which is often expressed at the surface of mast cells. This protein has an immunoreceptor tyrosine-based inhibitory motif in cytoplasmic region and acts as a negative regulator of FcεRI-dependent signaling pathway in mast cells [66,67]. Mast cells are abundant cells in central nervous system and contain pro-inflammatory mediators such as histamine, cytokines, ATP, proteases and leukotrienes, which are stored in their internal granules. Anaphylaxis is an immediate hypersensitivity reaction that is started with antigen-carrying immunoglobulin E binding to FcεRI receptor and results in the degranulation of mast cells and secretion of pro-inflammatory mediators to extracellular space [68,69]. Some report suggest that amyloid peptides induce mast cell degranulation response [70,71]. The resulting release of different pro-inflammatory molecules from mast cells may be associated to the onset or even progress of AD [71].

*H2-Ob* is the switching gene in the 72^nd^ triplet. HLA-DOB is the orthologous of this gene in human, which is related to MHC class II. This protein is commonly expressed in professional antigen presenting cells such as macrophages, B cells and specific dendritic cells. The importance of HLA-DOB gene has been reported in a wide range of disease, including schizophrenia [72], type I diabetes, [73], rheumatoid arthritis [74], Kawasaki disease [75], multiple myeloma [76] and chronic lymphocytic leukemia [77]. Furthermore, two genes associated with immune system, namely HLA-DRB5 and HLA-DRB1, which are both related to MHC class II are suggested to be linked to AD [78]. Therefore, HLA-DOB might be involved in AD.

As shown in Fig 1, when normalized expression level of *H2-Ob* gene is between 0.37 and 1.84 (as in the most AD samples (see S1 Table)), there is a direct correlation between *Milr1* and *Csf1r* expression levels. Note that *Milr1* and *Csf1r* are associated with biological processes “negative regulation of FcεRI-dependent signaling pathway” and “proliferation and activation of microglial cells”, respectively. Therefore, the positive correlation between *Milr1* and *Csf1r* expression levels implies that the two above-mentioned biological processes are activated in concert with each other. In contrast, when normalized expression level of *H2-Ob* gene is between -1.84 and -0.37 (as in the most wild type samples (see S1 Table)), the two mentioned processes probably act in opposite directions, because of the inverse correlation between *Milr1* and *Csf1r* expression levels. In other words, changes in expression level of *H2-Ob* gene can act as the switching factor for altering the cellular behavior. The homeostasis-related link between mast cells and microglia is presumably controlled with *H2-Ob* as switching genes in AD. See below.

Mast cells have an important role in starting the inflammation of central nervous system and are the first responder cells to the brain injury [79]. Mast cells act as the inducer for microglial activation. It is previously reported that mast cell activation results in induction of microglia for releasing neurotrophin [80]. Moreover, it is reported that induction of microglia activation is done via histamine and tryptase, which are inflammation mediators released by mast cells. Moreover, they showed that activated mast cells directly cause the activation of microglia [81–83].

Mast cells and microglial cells have endogenous homeostatic molecules that can be over-produced as a result of tissue destruction or induction of inflammatory responses. Palmitoylethanolamide, which is produced and hydrolyzed by microglia [84], has a key role in maintaining cellular homeostasis through inhibition of mast cell function, especially when there is an exogenously induced stress such as inflammation [85,86].

Taking into account the mast cell-microglia communications and the importance of homeostasis for survival, one can conclude that the same direction of two biological processes “negative regulation of FcεRI-dependent signaling pathway” and “proliferation and activation of microglial cells”, which is observed in the 72^nd^ triplet in conditions related to AD, is indicative of a type of homeostatic mechanism in the cell that occurs due to changes in expression level of *H2-Ob* gene. Following the increase in activation of mast cells as the first responder cells to brain damages in AD, up-regulation of *H2-Ob* gene, either directly or through mediators, may cause change in the correlation of *Csf1r* and *Milr1* expression levels. As a result, the biological process of “proliferation and activation of microglial cells”, which happens in response to disease-related factors such as β-amyloid plaques, and the biological process of “negative regulation of FcεRI-dependent signaling pathway”, which is related to maintaining cellular homeostasis, may act in the same direction. In contrast, in normal condition, as mast cells are inactive and these cells are in steady condition. Therefore, an inverse correlation is expected between *Csf1r* and *Milr1* genes when the expression level of *H2-Ob* is low. Consequently it is expected that the biological processes related to these two genes act in the opposite directions probably.

### The possible link between *Csf1r* and neuronal loss

*Csf1r* has been previously suggested to be involved in microglia activation, and therefore, it was suggested to be a drug target for AD [87]. Interestingly, there are two recent studies in which inhibition of *Csf1r*, which in turn results in eliminating microglia, is investigated. In the first study, Spangenberg and coworkers (2016) found that *Csf1r* inhibition prevents neuronal loss in AD mice model [65]. On the other hand, Hilla and coworker (2017) have observed that *Csf1r* inhibition does not have a preventing effect on neuronal loss [88]. These seemingly contradictory observations should certainly be studied more carefully. A possible explanation for these observations might be that *Csf1r* inhibition may not be the only determinant of the neuronal loss. In other words, if we assume that there are other players involved in this process one would expect that the simultaneous activity of both proteins, and not *Csf1r* alone, may determine neuronal degeneration. Based on our results, we already know that the cell involves a switch to fine-tune the simultaneous activity of *Csf1r* and *Milr1*. This switch, or other similar switches might be involved in the process of neuronal degeneration, and explain why neuronal loss cannot simply be inferred from *Csf1r* activity alone.

## Conclusion and future work

A huge number of disease-related high-throughput “omics” datasets are freely available today. Such datasets contain valuable information about disease-related pathways and their corresponding gene interactions. In the present study, for the first time, we used the three-way interaction model for tracing the switching genes involved AD. The advantage of this approach compared to the pairwise co-expression analysis is that the three-way interaction model can cope with the dynamic nature of co-expression relations. Hence, the three-way interaction model can potentially shed light on some of the (other) causes of cellular alterations. More specifically, in the present study, we suggest that *H2-Ob* as the switching gene and the gene pair {*Csf1r*, *Milr1*} form a statistically significant and biologically relevant triplet in AD. The relationship among the pathways associated with these three genes reveals that two biological processes, namely “negative regulation of FcεRI-dependent signaling pathway” and “proliferation and activation of microglial cells” are in the same direction in conditions related to AD. This is indicative of a type of homeostatic mechanism in the cell that occurs when *H2-Ob* is up-regulated.

In the next step, we plan to experimentally validate the relationship between *H2-Ob* gene and the {*Csf1r*, *Milr1*} gene pair.

## Supporting information

S1 Table Differentially-expressed genes. (PDF)

S2 Table Statistically significant triplets. (PDF)

S3 Table Comparison of GSEA results in group1, 2 and random groups. (PDF)

S4 Table Gene set enrichment analysis results based on biological process, cellular component, and KEGG pathway. (PDF)

S5 Table The categorized biological processes based on the AD-related pathways. (PDF)

S1 Fig This file includes array images, MA plots, box plots and data histograms before and after of normalization. (PDF)

S2 Fig The *p*-value histogram of the top 300000 three-way interactions. (PDF)

S3 Fig Statistically significant triplets Network. (PDF)

S4 Fig Scatter plots of 12 triplets in which X_1_ and X_2_ are involved in the same biological process. (PDF)

S1 Text This file includes “the reasons for choosing the mouse rather than human dataset” (Section A) and “comparison of present study results with the Lopez-Gonzalez et al.’s study” (Section B). (PDF)

S1 File This file includes “figure of Gene regulatory network” (Section A) and “the steps of construction gene regulatory network” (Section B). (PDF)

## References

[1] Wirz K, Keitel S, Swaab DF, Verhaagen J, Bossers K (2014) Early molecular changes in Alzheimer disease: can we catch the disease in its presymptomatic phase. J Alzheimers Dis 38: 719–740. doi: 10.3233/JAD-130920 24072070 10.3233/JAD-130920

[2] Xu Z, Dong Y, Wang H, Culley DJ, Marcantonio ER, et al (2014) Age-dependent postoperative cognitive impairment and Alzheimer-related neuropathology in mice. Scientific reports 4: 3766 doi: 10.1038/srep03766 24441878 10.1038/srep03766 PMC3895908

[3] Korolainen MA, Nyman TA, Aittokallio T, Pirttila T (2010) An update on clinical proteomics in Alzheimer's research. J Neurochem 112: 1386–1414. doi: 10.1111/j.1471-4159.2009.06558.x 20050976 10.1111/j.1471-4159.2009.06558.x

[4] Geula C, Mesulam MM (1995) Cholinesterases and the pathology of Alzheimer disease. Alzheimer Dis Assoc Disord 9: 23–28. 8534419 10.1097/00002093-199501002-00005

[5] Raschetti R, Albanese E, Vanacore N, Maggini M (2007) Cholinesterase inhibitors in mild cognitive impairment: a systematic review of randomised trials. PLoS Med 4: e338 doi: 10.1371/journal.pmed.0040338 18044984 10.1371/journal.pmed.0040338 PMC2082649

[6] Eisen MB, Spellman PT, Brown PO, Botstein D (1998) Cluster analysis and display of genome-wide expression patterns. Proc Natl Acad Sci U S A 95: 14863–14868. 9843981 10.1073/pnas.95.25.14863 PMC24541

[7] Tamayo P, Slonim D, Mesirov J, Zhu Q, Kitareewan S, et al (1999) Interpreting patterns of gene expression with self-organizing maps: methods and application to hematopoietic differentiation. Proc Natl Acad Sci U S A 96: 2907–2912. 10077610 10.1073/pnas.96.6.2907 PMC15868

[8] Alter O, Brown PO, Botstein D (2000) Singular value decomposition for genome-wide expression data processing and modeling. Proc Natl Acad Sci U S A 97: 10101–10106. 10963673 10.1073/pnas.97.18.10101 PMC27718

[9] Marcotte EM, Pellegrini M, Thompson MJ, Yeates TO, Eisenberg D (1999) A combined algorithm for genome-wide prediction of protein function. Nature 402: 83–86. doi: 10.1038/47048 10573421 10.1038/47048

[10] Zhou XJ, Kao MC, Huang H, Wong A, Nunez-Iglesias J, et al (2005) Functional annotation and network reconstruction through cross-platform integration of microarray data. Nat Biotechnol 23: 238–243. doi: 10.1038/nbt1058 15654329 10.1038/nbt1058

[11] Stuart JM, Segal E, Koller D, Kim SK (2003) A gene-coexpression network for global discovery of conserved genetic modules. science 302: 249–255. doi: 10.1126/science.1087447 12934013 10.1126/science.1087447

[12] Lee HK, Hsu AK, Sajdak J, Qin J, Pavlidis P (2004) Coexpression analysis of human genes across many microarray data sets. Genome research 14: 1085–1094. doi: 10.1101/gr.1910904 15173114 10.1101/gr.1910904 PMC419787

[13] Li KC (2002) Genome-wide coexpression dynamics: theory and application. Proc Natl Acad Sci U S A 99: 16875–16880. doi: 10.1073/pnas.252466999 12486219 10.1073/pnas.252466999 PMC139237

[14] Choi JK, Yu U, Yoo OJ, Kim S (2005) Differential coexpression analysis using microarray data and its application to human cancer. Bioinformatics 21: 4348–4355. doi: 10.1093/bioinformatics/bti722 16234317 10.1093/bioinformatics/bti722

[15] Rao CV, Arkin AP (2001) Control motifs for intracellular regulatory networks. Annu Rev Biomed Eng 3: 391–419. doi: 10.1146/annurev.bioeng.3.1.391 11447069 10.1146/annurev.bioeng.3.1.391

[16] Bowers PM, Cokus SJ, Eisenberg D, Yeates TO (2004) Use of logic relationships to decipher protein network organization. Science 306: 2246–2249. doi: 10.1126/science.1103330 15618515 10.1126/science.1103330

[17] Lopez-Gonzalez I, Schluter A, Aso E, Garcia-Esparcia P, Ansoleaga B, et al (2015) Neuroinflammatory signals in Alzheimer disease and APP/PS1 transgenic mice: correlations with plaques, tangles, and oligomeric species. J Neuropathol Exp Neurol 74: 319–344. doi: 10.1097/NEN.0000000000000176 25756590 10.1097/NEN.0000000000000176

[18] Ritchie ME, Phipson B, Wu D, Hu Y, Law CW, et al (2015) limma powers differential expression analyses for RNA-sequencing and microarray studies. Nucleic Acids Res 43: e47 doi: 10.1093/nar/gkv007 25605792 10.1093/nar/gkv007 PMC4402510

[19] Gentleman R, Carey V, Huber W, Hahne F (2015) Genefilter: methods for filtering genes from high-throughput experiments. R package version 1.

[20] Benjamini Y, Drai D, Elmer G, Kafkafi N, Golani I (2001) Controlling the false discovery rate in behavior genetics research. Behavioural brain research 125: 279–284. 11682119 10.1016/s0166-4328(01)00297-2

[21] Gunderson T, Ho YY (2014) An efficient algorithm to explore liquid association on a genome-wide scale. BMC Bioinformatics 15: 371 doi: 10.1186/s12859-014-0371-5 25431229 10.1186/s12859-014-0371-5 PMC4255454

[22] Ho YY, Parmigiani G, Louis TA, Cope LM (2011) Modeling liquid association. Biometrics 67: 133–141. doi: 10.1111/j.1541-0420.2010.01440.x 20528865 10.1111/j.1541-0420.2010.01440.x

[23] Willse JT, Willse MJT (2014) Package ‘CTT’.

[24] Subramanian A, Tamayo P, Mootha VK, Mukherjee S, Ebert BL, et al (2005) Gene set enrichment analysis: a knowledge-based approach for interpreting genome-wide expression profiles. Proceedings of the National Academy of Sciences 102: 15545–15550. 10.1073/pnas.0506580102 PMC1239896 16199517

[25] Kanehisa M, Goto S, Sato Y, Furumichi M, Tanabe M (2012) KEGG for integration and interpretation of large-scale molecular data sets. Nucleic Acids Res 40: D109–114. doi: 10.1093/nar/gkr988 22080510 10.1093/nar/gkr988 PMC3245020

[26] Bindea G, Mlecnik B, Hackl H, Charoentong P, Tosolini M, et al (2009) ClueGO: a Cytoscape plug-in to decipher functionally grouped gene ontology and pathway annotation networks. Bioinformatics 25: 1091–1093. doi: 10.1093/bioinformatics/btp101 19237447 10.1093/bioinformatics/btp101 PMC2666812

[27] Shannon P, Markiel A, Ozier O, Baliga NS, Wang JT, et al (2003) Cytoscape: a software environment for integrated models of biomolecular interaction networks. Genome Res 13: 2498–2504. doi: 10.1101/gr.1239303 14597658 10.1101/gr.1239303 PMC403769

[28] Remo A, Simeone I, Pancione M, Parcesepe P, Finetti P, et al (2015) Systems biology analysis reveals NFAT5 as a novel biomarker and master regulator of inflammatory breast cancer. Journal of translational medicine 13: 138 doi: 10.1186/s12967-015-0492-2 25928084 10.1186/s12967-015-0492-2 PMC4438533

[29] Margolin AA, Nemenman I, Basso K, Wiggins C, Stolovitzky G, et al (2006) ARACNE: an algorithm for the reconstruction of gene regulatory networks in a mammalian cellular context. BMC Bioinformatics 7 S7. 10.1186/1471-2105-7-S1-S7 PMC1810318 16723010

[30] Ray M, Zhang W (2010) Analysis of Alzheimer's disease severity across brain regions by topological analysis of gene co-expression networks. BMC systems biology 4: 136 doi: 10.1186/1752-0509-4-136 20925940 10.1186/1752-0509-4-136 PMC2976747

[31] Zhang B, Gaiteri C, Bodea L-G, Wang Z, McElwee J, et al (2013) Integrated systems approach identifies genetic nodes and networks in late-onset Alzheimer’s disease. Cell 153: 707–720. doi: 10.1016/j.cell.2013.03.030 23622250 10.1016/j.cell.2013.03.030 PMC3677161

[32] Ray M, Ruan J, Zhang W (2008) Variations in the transcriptome of Alzheimer’s disease reveal molecular networks involved in cardiovascular diseases. Genome Biol 9: R148 doi: 10.1186/gb-2008-9-10-r148 18842138 10.1186/gb-2008-9-10-r148 PMC2760875

[33] Lazar MA (2003) Thyroid hormone action: a binding contract. J Clin Invest 112: 497–499. doi: 10.1172/JCI19479 12925689 10.1172/JCI19479 PMC171396

[34] Zhang J, Ji Y, Zhang L (2007) Extracting three-way gene interactions from microarray data. Bioinformatics 23: 2903–2909. doi: 10.1093/bioinformatics/btm482 17921496 10.1093/bioinformatics/btm482

[35] Kayano M, Takigawa I, Shiga M, Tsuda K, Mamitsuka H (2009) Efficiently finding genome-wide three-way gene interactions from transcript- and genotype-data. Bioinformatics 25: 2735–2743. doi: 10.1093/bioinformatics/btp531 19736252 10.1093/bioinformatics/btp531 PMC2781753

[36] Pei J-J, Braak H, An W-L, Winblad B, Cowburn RF, et al (2002) Up-regulation of mitogen-activated protein kinases ERK1/2 and MEK1/2 is associated with the progression of neurofibrillary degeneration in Alzheimer’s disease. Molecular brain research 109: 45–55. 12531514 10.1016/s0169-328x(02)00488-6

[37] Ferrer I, Blanco R, Carmona M, Ribera R, Goutan E, et al (2001) Phosphorylated map kinase (ERK1, ERK2) expression is associated with early tau deposition in neurones and glial cells, but not with increased nuclear DNA vulnerability and cell death, in Alzheimer disease, Pick's disease, progressive supranuclear palsy and corticobasal degeneration. Brain Pathol 11: 144–158. 11303790 10.1111/j.1750-3639.2001.tb00387.x PMC8098611

[38] Martin L, Latypova X, Wilson CM, Magnaudeix A, Perrin ML, et al (2013) Tau protein kinases: involvement in Alzheimer's disease. Ageing Res Rev 12: 289–309. doi: 10.1016/j.arr.2012.06.003 22742992 10.1016/j.arr.2012.06.003

[39] D'Andrea MR, Cole GM, Ard MD (2004) The microglial phagocytic role with specific plaque types in the Alzheimer disease brain. Neurobiol Aging 25: 675–683. doi: 10.1016/j.neurobiolaging.2003.12.026 15172747 10.1016/j.neurobiolaging.2003.12.026

[40] Rogers J, Strohmeyer R, Kovelowski CJ, Li R (2002) Microglia and inflammatory mechanisms in the clearance of amyloid beta peptide. Glia 40: 260–269. doi: 10.1002/glia.10153 12379913 10.1002/glia.10153

[41] Salminen A, Ojala J, Kauppinen A, Kaarniranta K, Suuronen T (2009) Inflammation in Alzheimer's disease: amyloid-beta oligomers trigger innate immunity defence via pattern recognition receptors. Prog Neurobiol 87: 181–194. 19388207 10.1016/j.pneurobio.2009.01.001

[42] Rentzos M, Rombos A (2012) The role of IL-15 in central nervous system disorders. Acta Neurol Scand 125: 77–82. doi: 10.1111/j.1600-0404.2011.01524.x 21615353 10.1111/j.1600-0404.2011.01524.x

[43] Ciaramella A, Bizzoni F, Salani F, Vanni D, Spalletta G, et al (2010) Increased pro-inflammatory response by dendritic cells from patients with Alzheimer's disease. J Alzheimers Dis 19: 559–572. doi: 10.3233/JAD-2010-1257 20110602 10.3233/JAD-2010-1257

[44] Mirza Z, Kamal MA, Al-Qahtani MH, Karim S (2014) Establishing genomic/transcriptomic links between Alzheimer’s disease and type 2 diabetes mellitus by meta-analysis approach. CNS Neurol Disord Drug Targets 13: 501–516. 24059308 10.2174/18715273113126660154

[45] Liu ZP, Wang Y, Zhang XS, Chen L (2010) Identifying dysfunctional crosstalk of pathways in various regions of Alzheimer's disease brains. BMC Syst Biol 4 S11 doi: 10.1186/1752-0509-4-S2-S11 20840725 10.1186/1752-0509-4-S2-S11 PMC2982685

[46] Heppner FL, Ransohoff RM, Becher B (2015) Immune attack: the role of inflammation in Alzheimer disease. Nature Reviews Neuroscience 16: 358–372. doi: 10.1038/nrn3880 25991443 10.1038/nrn3880

[47] Alcolea D, Martínez-Lage P, Sánchez-Juan P, Olazarán J, Antúnez C, et al (2015) Amyloid precursor protein metabolism and inflammation markers in preclinical Alzheimer disease. Neurology 85: 626–633. doi: 10.1212/WNL.0000000000001859 26180139 10.1212/WNL.0000000000001859

[48] Epstein FH, Choy EH, Panayi GS (2001) Cytokine pathways and joint inflammation in rheumatoid arthritis. New England Journal of Medicine 344: 907–916. doi: 10.1056/NEJM200103223441207 11259725 10.1056/NEJM200103223441207

[49] Sanmartin C, Plano D, Font M, Palop JA (2011) Selenium and clinical trials: new therapeutic evidence for multiple diseases. Curr Med Chem 18: 4635–4650. 21864284 10.2174/092986711797379249

[50] Myllykangas-Luosujärvi R, Isomäki H (1994) Alzheimer's disease and rheumatoid arthritis. Rheumatology 33: 501–502. 10.1093/rheumatology/33.5.501 8173862

[51] Boyd TD, Bennett SP, Mori T, Governatori N, Runfeldt M, et al (2010) GM-CSF upregulated in rheumatoid arthritis reverses cognitive impairment and amyloidosis in Alzheimer mice. Journal of Alzheimer's Disease 21: 507–518. doi: 10.3233/JAD-2010-091471 20555144 10.3233/JAD-2010-091471 PMC5588158

[52] Fernandez-Flores A, Rodriguez-Peralto JL (2016) Morphological and immunohistochemical clues for the diagnosis of cutaneous leishmaniasis and the interpretation of CD1a status. J Am Acad Dermatol 74: 536–543. doi: 10.1016/j.jaad.2015.09.038 26577511 10.1016/j.jaad.2015.09.038

[53] Heneka MT, Golenbock DT, Latz E (2015) Innate immunity in Alzheimer's disease. Nat Immunol 16: 229–236. doi: 10.1038/ni.3102 25689443 10.1038/ni.3102

[54] Takashima A (2006) GSK-3 is essential in the pathogenesis of Alzheimer's disease. Journal of Alzheimer's Disease 9: 309–317. 16914869 10.3233/jad-2006-9s335

[55] Garcia I, Fall Y, Gomez G, Gonzalez-Diaz H (2011) First computational chemistry multi-target model for anti-Alzheimer, anti-parasitic, anti-fungi, and anti-bacterial activity of GSK-3 inhibitors in vitro, in vivo, and in different cellular lines. Mol Divers 15: 561–567. doi: 10.1007/s11030-010-9280-3 20931280 10.1007/s11030-010-9280-3

[56] Adamec E, Mohan PS, Cataldo AM, Vonsattel J, Nixon R (2000) Up-regulation of the lysosomal system in experimental models of neuronal injury: implications for Alzheimer’s disease. Neuroscience 100: 663–675. 11098128 10.1016/s0306-4522(00)00281-5

[57] Nixon RA, Cataldo AM (2006) Lysosomal system pathways: genes to neurodegeneration in Alzheimer's disease. J Alzheimers Dis 9: 277–289. 16914867 10.3233/jad-2006-9s331

[58] Goetzl EJ, Boxer A, Schwartz JB, Abner EL, Petersen RC, et al (2015) Altered lysosomal proteins in neural-derived plasma exosomes in preclinical Alzheimer disease. Neurology 85: 40–47. doi: 10.1212/WNL.0000000000001702 26062630 10.1212/WNL.0000000000001702 PMC4501943

[59] Akiyama H, Barger S, Barnum S, Bradt B, Bauer J, et al (2000) Inflammation and Alzheimer's disease. Neurobiol Aging 21: 383–421. 10858586 10.1016/s0197-4580(00)00124-x PMC3887148

[60] Edison P, Archer HA, Gerhard A, Hinz R, Pavese N, et al (2008) Microglia, amyloid, and cognition in Alzheimer's disease: An [11C](R)PK11195-PET and [11C]PIB-PET study. Neurobiol Dis 32: 412–419. doi: 10.1016/j.nbd.2008.08.001 18786637 10.1016/j.nbd.2008.08.001

[61] Fernandez-Botran R, Ahmed Z, Crespo FA, Gatenbee C, Gonzalez J, et al (2011) Cytokine expression and microglial activation in progressive supranuclear palsy. Parkinsonism Relat Disord 17: 683–688. doi: 10.1016/j.parkreldis.2011.06.007 21741294 10.1016/j.parkreldis.2011.06.007 PMC3196843

[62] Akiyama H, Nishimura T, Kondo H, Ikeda K, Hayashi Y, et al (1994) Expression of the receptor for macrophage colony stimulating factor by brain microglia and its upregulation in brains of patients with Alzheimer's disease and amyotrophic lateral sclerosis. Brain Res 639: 171–174. 7514086 10.1016/0006-8993(94)91779-5

[63] Murphy GM Jr., Zhao F, Yang L, Cordell B (2000) Expression of macrophage colony-stimulating factor receptor is increased in the AbetaPP(V717F) transgenic mouse model of Alzheimer's disease. Am J Pathol 157: 895–904. 10980129 10.1016/s0002-9440(10)64603-2 PMC1885684

[64] Olmos-Alonso A, Schetters ST, Sri S, Askew K, Mancuso R, et al (2016) Pharmacological targeting of CSF1R inhibits microglial proliferation and prevents the progression of Alzheimer's-like pathology. Brain 139: 891–907. doi: 10.1093/brain/awv379 26747862 10.1093/brain/awv379 PMC4766375

[65] Spangenberg EE, Lee RJ, Najafi AR, Rice RA, Elmore MR, et al (2016) Eliminating microglia in Alzheimer's mice prevents neuronal loss without modulating amyloid-beta pathology. Brain 139: 1265–1281. doi: 10.1093/brain/aww016 26921617 10.1093/brain/aww016 PMC5006229

[66] Hitomi K, Tahara-Hanaoka S, Someya S, Fujiki A, Tada H, et al (2010) An immunoglobulin-like receptor, Allergin-1, inhibits immunoglobulin E-mediated immediate hypersensitivity reactions. Nat Immunol 11: 601–607. doi: 10.1038/ni.1886 20526344 10.1038/ni.1886

[67] Nagai K, Tahara-Hanaoka S, Morishima Y, Tokunaga T, Imoto Y, et al (2013) Expression and function of Allergin-1 on human primary mast cells. PLoS One 8: e76160 doi: 10.1371/journal.pone.0076160 24116093 10.1371/journal.pone.0076160 PMC3792105

[68] Metcalfe DD, Baram D, Mekori YA (1997) Mast cells. Physiol Rev 77: 1033–1079. 9354811 10.1152/physrev.1997.77.4.1033

[69] Metcalfe DD, Peavy RD, Gilfillan AM (2009) Mechanisms of mast cell signaling in anaphylaxis. Journal of Allergy and Clinical Immunology 124: 639–646. doi: 10.1016/j.jaci.2009.08.035 19815110 10.1016/j.jaci.2009.08.035 PMC2788154

[70] Niederhoffer N, Levy R, Sick E, Andre P, Coupin G, et al (2009) Amyloid beta peptides trigger CD47-dependent mast cell secretory and phagocytic responses. Int J Immunopathol Pharmacol 22: 473–483. doi: 10.1177/039463200902200224 19505377 10.1177/039463200902200224

[71] Harcha PA, Vargas A, Yi C, Koulakoff AA, Giaume C, et al (2015) Hemichannels Are Required for Amyloid beta-Peptide-Induced Degranulation and Are Activated in Brain Mast Cells of APPswe/PS1dE9 Mice. J Neurosci 35: 9526–9538. doi: 10.1523/JNEUROSCI.3686-14.2015 26109673 10.1523/JNEUROSCI.3686-14.2015 PMC6605189

[72] Zhang Z, Yu H, Jiang S, Liao J, Lu T, et al (2015) Evidence for Association of Cell Adhesion Molecules Pathway and NLGN1 Polymorphisms with Schizophrenia in Chinese Han Population. PLoS One 10: e0144719 doi: 10.1371/journal.pone.0144719 26674772 10.1371/journal.pone.0144719 PMC4682938

[73] Qiu YH, Deng FY, Tang ZX, Jiang ZH, Lei SF (2015) Functional relevance for type 1 diabetes mellitus-associated genetic variants by using integrative analyses. Hum Immunol 76: 753–758. doi: 10.1016/j.humimm.2015.09.033 26429317 10.1016/j.humimm.2015.09.033

[74] Jiang X, Kallberg H, Chen Z, Arlestig L, Rantapaa-Dahlqvist S, et al (2016) An Immunochip-based interaction study of contrasting interaction effects with smoking in ACPA-positive versus ACPA-negative rheumatoid arthritis. Rheumatology (Oxford) 55: 149–155. 26272072 10.1093/rheumatology/kev285

[75] Shendre A, Wiener HW, Zhi D, Vazquez AI, Portman MA, et al (2014) High-density genotyping of immune loci in Kawasaki disease and IVIG treatment response in European-American case-parent trio study. Genes Immun 15: 534–542. doi: 10.1038/gene.2014.47 25101798 10.1038/gene.2014.47 PMC4257866

[76] Kang YJ, Zeng W, Song W, Reinhold B, Choi J, et al (2013) Identification of human leucocyte antigen (HLA)-A*0201-restricted cytotoxic T lymphocyte epitopes derived from HLA-DObeta as a novel target for multiple myeloma. Br J Haematol 163: 343–351. doi: 10.1111/bjh.12544 24032635 10.1111/bjh.12544 PMC4137325

[77] Souwer Y, Chamuleau ME, van de Loosdrecht AA, Tolosa E, Jorritsma T, et al (2009) Detection of aberrant transcription of major histocompatibility complex class II antigen presentation genes in chronic lymphocytic leukaemia identifies HLA-DOA mRNA as a prognostic factor for survival. Br J Haematol 145: 334–343. doi: 10.1111/j.1365-2141.2009.07625.x 19245431 10.1111/j.1365-2141.2009.07625.x

[78] Lambert JC, Ibrahim-Verbaas CA, Harold D, Naj AC, Sims R, et al (2013) Meta-analysis of 74,046 individuals identifies 11 new susceptibility loci for Alzheimer's disease. Nat Genet 45: 1452–1458. doi: 10.1038/ng.2802 24162737 10.1038/ng.2802 PMC3896259

[79] Lindsberg PJ, Strbian D, Karjalainen-Lindsberg ML (2010) Mast cells as early responders in the regulation of acute blood-brain barrier changes after cerebral ischemia and hemorrhage. J Cereb Blood Flow Metab 30: 689–702. doi: 10.1038/jcbfm.2009.282 20087366 10.1038/jcbfm.2009.282 PMC2949160

[80] Yuan H, Zhu X, Zhou S, Chen Q, Zhu X, et al (2010) Role of mast cell activation in inducing microglial cells to release neurotrophin. J Neurosci Res 88: 1348–1354. doi: 10.1002/jnr.22304 20025063 10.1002/jnr.22304

[81] Zhang S, Zeng X, Yang H, Hu G, He S (2012) Mast cell tryptase induces microglia activation via protease-activated receptor 2 signaling. Cell Physiol Biochem 29: 931–940. doi: 10.1159/000171029 22613992 10.1159/000171029

[82] Zhang X, Wang Y, Dong H, Xu Y, Zhang S (2016) Induction of Microglial Activation by Mediators Released from Mast Cells. Cell Physiol Biochem 38: 1520–1531. doi: 10.1159/000443093 27050634 10.1159/000443093

[83] Dong H, Zhang W, Zeng X, Hu G, Zhang H, et al (2014) Histamine induces upregulated expression of histamine receptors and increases release of inflammatory mediators from microglia. Mol Neurobiol 49: 1487–1500. doi: 10.1007/s12035-014-8697-6 24752587 10.1007/s12035-014-8697-6

[84] Muccioli GG, Stella N (2008) Microglia produce and hydrolyze palmitoylethanolamide. Neuropharmacology 54: 16–22. doi: 10.1016/j.neuropharm.2007.05.015 17631917 10.1016/j.neuropharm.2007.05.015 PMC2254322

[85] Facci L, Dal Toso R, Romanello S, Buriani A, Skaper SD, et al (1995) Mast cells express a peripheral cannabinoid receptor with differential sensitivity to anandamide and palmitoylethanolamide. Proc Natl Acad Sci U S A 92: 3376–3380. 7724569 10.1073/pnas.92.8.3376 PMC42169

[86] Cerrato S, Brazis P, della Valle MF, Miolo A, Puigdemont A (2010) Effects of palmitoylethanolamide on immunologically induced histamine, PGD2 and TNFalpha release from canine skin mast cells. Vet Immunol Immunopathol 133: 9–15. doi: 10.1016/j.vetimm.2009.06.011 19625089 10.1016/j.vetimm.2009.06.011

[87] Wes PD, Sayed FA, Bard F, Gan L (2016) Targeting microglia for the treatment of Alzheimer's Disease. Glia 64: 1710–1732. doi: 10.1002/glia.22988 27100611 10.1002/glia.22988

[88] Hilla AM, Diekmann H, Fischer D (2017) Microglia Are Irrelevant for Neuronal Degeneration and Axon Regeneration after Acute Injury. J Neurosci 37: 6113–6124. doi: 10.1523/JNEUROSCI.0584-17.2017 28539419 10.1523/JNEUROSCI.0584-17.2017 PMC6596505

