# Supplementary material for: Three-way interaction model to trace the mechanisms involved in Alzheimer’s disease transgenic mice
Source: PLoS One. 2017 Sep 21;12(9):e0184697. doi: 10.1371/journal.pone.0184697 (PMC5608283; doi:10.1371/journal.pone.0184697)
Supplement: S1 Table — (PDF) [file pone.0184697.s001.pdf]

S1 Table

Gene ID	logFC	AveExpr	t	P.Value	adj.P.Val	B
10476314	-0.856701	11.71713	-25.86958	9.93E-23	2.04E-18	28.80812
10440491	-0.357787	12.22343	-14.99706	7.35E-16	7.55E-12	21.08195
10462140	-0.295892	5.642927	-6.413136	3.60E-07	0.0024666	6.139441
10597323	0.29213	10.57244	6.098439	8.83E-07	0.0045329	5.376427
10476945	-3.092731	5.391644	-5.893587	1.59E-06	0.0065205	4.874309
10389222	-1.202551	5.550457	-5.743021	2.45E-06	0.0083746	4.502922
10508074	-0.590065	5.306639	-5.428505	6.06E-06	0.0177627	3.722035
10461721	-0.760083	7.08012	-5.310745	8.51E-06	0.0215702	3.428318
10424349	-0.268076	9.074391	-5.25934	9.87E-06	0.0215702	3.299942
10447602	0.219145	8.581799	5.237547	1.05E-05	0.0215702	3.245493
10389231	-2.264374	6.525541	-5.142362	1.38E-05	0.023655	3.007538
10440393	-0.440652	5.488662	-5.144896	1.37E-05	0.023655	3.013876
10397645	-0.357986	3.392303	-5.063509	1.74E-05	0.02741	2.810291
10548375	-1.215191	4.189705	-4.982532	2.19E-05	0.0284404	2.60768
10557895	-1.023737	4.222646	-4.957605	2.36E-05	0.0284404	2.54531
10360040	-0.471475	7.491854	-4.983547	2.19E-05	0.0284404	2.610221
10441003	-0.382196	5.27729	-4.95879	2.35E-05	0.0284404	2.548276
10425866	-0.372952	6.52628	-4.86068	3.11E-05	0.0355032	2.302839
10347335	-0.711768	5.56916	-4.759682	4.16E-05	0.0367281	2.050349
10459866	-0.470627	7.939773	-4.78171	3.91E-05	0.0367281	2.105397
10545101	-0.413176	6.529246	-4.748766	4.29E-05	0.0367281	2.023075
10519951	-0.230227	6.371864	-4.760256	4.15E-05	0.0367281	2.051784
10505517	-0.222519	3.985566	-4.798915	3.72E-05	0.0367281	2.1484
10445753	0.163737	3.690067	4.80479	3.66E-05	0.0367281	2.163088
10461558	-0.483561	5.09931	-4.724831	4.60E-05	0.0377663	1.963287
10445781	-0.727762	7.843383	-4.690719	5.07E-05	0.0395611	1.878114
10404606	-0.645737	9.288288	-4.656874	5.59E-05	0.0395611	1.793653
10422496	-0.357961	5.018882	-4.658394	5.56E-05	0.0395611	1.797444
10532744	-0.277113	8.501885	-4.678997	5.25E-05	0.0395611	1.848856
10351658	-0.422659	4.132633	-4.626162	6.10E-05	0.0404115	1.717052
10444284	-0.368471	4.540117	-4.637457	5.91E-05	0.0404115	1.74522
10406928	-0.899604	6.544058	-4.604809	6.49E-05	0.0416159	1.663822
10496975	-0.297887	6.91831	-4.553565	7.51E-05	0.046723	1.536179
10412123	-0.326693	5.641624	-4.537848	7.86E-05	0.0474313	1.497059
10439312	-0.373165	5.036889	-4.516521	8.35E-05	0.0477534	1.444
10360227	-0.123109	10.94676	-4.515459	8.37E-05	0.0477534	1.441359
10416437	-0.371058	6.610778	-4.489276	9.02E-05	0.0487491	1.376262
10593646	-0.173563	7.551304	-4.498131	8.80E-05	0.0487491	1.398273
