# Supplementary material for: Three-way interaction model to trace the mechanisms involved in Alzheimer’s disease transgenic mice
Source: PLoS One. 2017 Sep 21;12(9):e0184697. doi: 10.1371/journal.pone.0184697 (PMC5608283; doi:10.1371/journal.pone.0184697)
Supplement: S2 Table — (PDF) [file pone.0184697.s002.pdf]

S2 Table

Trip	X1 or X2	X2 or X1	X3	rhodiff	MLA value	estimates	san.se	wald	pv	model	FDR(BH)
1	Bcl2a1c	Syngr2	Csf3r	1.4356	0.5195	1.2065	0.1938	38.746	4.83E-10	F	1.56E-04
2	Art2a-ps	Evc2	Tnfaip8l3	1.4687	0.5078	2.3485	0.3801	38.176	6.46E-10	F	1.56E-04
3	Nanp	Tceb1	Cd180	-1.5803	-0.5427	-2.7804	0.456	37.172	1.08E-09	F	1.56E-04
4	Cops7b	Cldn9	Prnp	-1.5663	-0.5146	-1.6122	0.2654	36.902	1.24E-09	F	1.56E-04
5	Sdsl	Apom	Prnp	1.5933	0.5466	1.9744	0.3277	36.304	1.69E-09	F	1.56E-04
6	Cfap20	Slc45a1	Slc14a1	-1.5898	-0.5718	-2.5907	0.4301	36.288	1.70E-09	F	1.56E-04
7	Aldoc	Cadm4	Lcp1	1.2552	0.5257	1.1067	0.1837	36.278	1.71E-09	F	1.56E-04
8	Zfp930	Zfp507	Dock8	-1.224	-0.5142	-1.4478	0.2404	36.254	1.73E-09	F	1.56E-04
9	Alox12e	Rbm33	Gpr183	1.8119	0.553	2.9552	0.4932	35.901	2.08E-09	F	1.66E-04
10	Cebpz	Zfp408	Parvg	-1.4396	-0.5444	-2.0229	0.3391	35.594	2.43E-09	F	1.75E-04
11	Ctsd	Spsb4	Ezr	1.2899	0.5343	1.6016	0.2707	35.01	3.28E-09	F	2.15E-04
12	Ndp	Vps51	Slc14a1	-1.3078	-0.5508	-1.996	0.3406	34.338	4.63E-09	F	2.78E-04
13	H2-Ob	Irf8	App	1.661	0.5744	2.7596	0.4722	34.152	5.10E-09	F	2.82E-04
14	Gm10620	Eif5	Pea15a	-1.3896	-0.5893	-1.2601	0.2172	33.649	6.60E-09	F	3.31E-04
15	E330034G19Rik	Sned1	Ccl6	-0.9592	-0.5399	-1.0875	0.1877	33.565	6.89E-09	F	3.31E-04
16	Il15	Letm1	Dock8	-1.5282	-0.5473	-2.5142	0.4396	32.716	1.07E-08	F	4.57E-04
17	Klf12	Mir21a	Parvg	1.5124	0.5308	2.6641	0.4659	32.694	1.08E-08	F	4.57E-04
18	Zbtb7c	Itpripl2	Tlr4	1.7239	0.5482	2.5822	0.4532	32.469	1.21E-08	F	4.85E-04
19	chr16:91712003-91712125	Ninl	Prnp	1.453	0.5209	2.1692	0.3823	32.196	1.39E-08	F	5.28E-04
20	Acvr1b	1500011B03Rik	Slc11a1	-1.6539	-0.5527	-2.8312	0.4997	32.099	1.47E-08	F	5.28E-04
21	Atp9a	D030056L22Rik	Gsap	1.6195	0.5524	1.8597	0.33	31.761	1.74E-08	F	5.83E-04
22	Hyou1	Tgfbr1	Ezr	1.3439	0.5311	1.8944	0.3373	31.547	1.95E-08	F	5.83E-04
23	Eya3	Psg20	Trem3	-1.5519	-0.5055	-2.127	0.3787	31.545	1.95E-08	F	5.83E-04
24	Scnn1g	Mir141	Slc11a1	1.5077	0.5181	1.6791	0.2995	31.438	2.06E-08	F	5.83E-04
25	Ncoa4	Bbs5	Lcp1	-1.3258	-0.5536	-2.0901	0.3728	31.427	2.07E-08	F	5.83E-04
26	Tm6sf2	Htr1a	Tnfaip8l3	-1.3284	-0.525	-1.6779	0.2998	31.331	2.18E-08	F	5.83E-04
27	4930426L09Rik	Osbpl6	Cd48	1.3116	0.5255	1.2269	0.2192	31.323	2.18E-08	F	5.83E-04
28	Olfr304	Pde3a	Cst7	-1.5216	-0.5544	-2.3102	0.4146	31.055	2.51E-08	F	6.37E-04
29	Nckap1l	Rdh11	Clec7a	1.402	0.5451	1.7974	0.3236	30.849	2.79E-08	F	6.37E-04
30	Serf1	Psd3	Dock8	1.5495	0.5112	1.7866	0.322	30.779	2.89E-08	F	6.37E-04
31	Olfr292	Mir144	Tnfaip8l3	-1.4088	-0.5012	-1.8151	0.3273	30.759	2.92E-08	F	6.37E-04

32	E230016K23Rik	Tmem26	Gpr65	-1.6394	-0.5692	-3.4107	0.6154	30.715	2.99E-08	F	6.37E-04
33	Inpp5b	Ap2s1	Slc14a1	-1.3555	-0.5506	-1.8495	0.3337	30.714	2.99E-08	F	6.37E-04
34	Fabp1	Rela	Sqle	-1.6218	-0.5132	-2.594	0.4684	30.667	3.06E-08	F	6.37E-04
35	Slc35e4	Fopnl	Gpr183	-1.1649	-0.5387	-1.313	0.2372	30.645	3.10E-08	F	6.37E-04
36	Olfr555	1700080E11Rik	Gsap	1.5273	0.5675	2.4172	0.4381	30.436	3.45E-08	F	6.60E-04
37	Tmprss11g	Fam175a	Ly86	-1.1073	-0.5339	-1.4578	0.2644	30.395	3.52E-08	F	6.60E-04
38	Cyp2j13	Txndc8	Slc15a3	-1.5847	-0.5234	-2.3436	0.4251	30.394	3.53E-08	F	6.60E-04
39	Tram1l1	9230110C19Rik	Ncf2	-1.3471	-0.523	-1.807	0.3281	30.338	3.63E-08	F	6.60E-04
40	Nptx1	Tyrp1	Clec7a	1.4792	0.5176	2.6795	0.4866	30.318	3.67E-08	F	6.60E-04
41	Mmp8	Gm839	Slc11a1	1.4019	0.5162	1.8343	0.3339	30.176	3.95E-08	F	6.72E-04
42	Olfr172	Igfbpl1	Ncf2	-1.6657	-0.6412	-2.7298	0.4982	30.02	4.28E-08	F	6.72E-04
43	Fam217a	Slc39a4	Gsap	1.4427	0.5559	1.986	0.3625	30.017	4.28E-08	F	6.72E-04
44	Sh3pxd2b	Nup98	Ncf2	-1.5155	-0.5373	-2.3694	0.4326	29.994	4.33E-08	F	6.72E-04
45	Klf12	Rasd2	Parvg	1.5408	0.5256	2.702	0.4937	29.955	4.42E-08	F	6.72E-04
46	Nsdhl	Ccl9	App	1.5275	0.5406	2.1232	0.3881	29.928	4.48E-08	F	6.72E-04
47	Abcf2	Sec24a	Dock8	-1.5758	-0.5307	-2.2136	0.4048	29.899	4.55E-08	F	6.72E-04
48	Ldlr	Fyb	H2-Ob	1.0407	0.5684	1.499	0.2742	29.894	4.56E-08	F	6.72E-04
49	Trpm6	Spink2	Parvg	-1.6765	-0.5448	-2.356	0.4309	29.889	4.58E-08	F	6.72E-04
50	Park7	Fam120b	Slc14a1	1.6956	0.564	2.3449	0.43	29.736	4.95E-08	F	7.13E-04
51	Olfr346	Dcaf8	Csf3r	-1.601	-0.5902	-2.1458	0.3941	29.648	5.18E-08	F	7.20E-04
52	Mroh4	Alpk1	Ly86	-1.5616	-0.5399	-1.6435	0.3019	29.64	5.20E-08	F	7.20E-04
53	Olfr146	Acbd6	Mpeg1	-1.4778	-0.5688	-2.2532	0.4149	29.49	5.62E-08	F	7.51E-04
54	Vmn2r-ps57	Prokr1	Cst7	1.4603	0.5641	3.1555	0.5811	29.485	5.63E-08	F	7.51E-04
55	Hrct1	Mir448	Ccl6	1.4167	0.5663	2.0305	0.3744	29.41	5.86E-08	F	7.60E-04
56	Flcn	Tjp3	App	1.6352	0.5181	2.4226	0.4468	29.393	5.91E-08	S	7.60E-04
57	Ccr1l1	Cox8c	App	1.3424	0.553	1.4076	0.2601	29.275	6.28E-08	F	7.73E-04
58	Blmh	Mknk2	Csf3r	-1.4651	-0.513	-2.0841	0.3852	29.273	6.29E-08	F	7.73E-04
59	Hirip3	Tmem79	Arpp21	1.4509	0.5304	2.0352	0.3769	29.164	6.65E-08	F	7.73E-04
60	Pglyrp1	Klf6	Runx1	-1.581	-0.5814	-1.9486	0.3609	29.159	6.67E-08	S	7.73E-04
61	Smek3	2900060B14Rik	Lcp1	1.6397	0.5261	2.0033	0.3711	29.147	6.71E-08	F	7.73E-04
62	Pgc	5430411C19Rik	Slc11a1	-1.3229	-0.5374	-1.9758	0.3662	29.118	6.81E-08	F	7.73E-04
63	Ccdc108	Qtrt1	Pea15a	0.9842	0.5458	1.4812	0.2748	29.048	7.06E-08	F	7.73E-04
64	Nxnl1	Kcnq4	Mpeg1	-1.3653	-0.5174	-1.894	0.3514	29.044	7.07E-08	F	7.73E-04

65	Lamb1	Tgfbi	Mpeg1	-1.647	-0.5326	-2.3672	0.4394	29.019	7.17E-08	F	7.73E-04
66	Klf14	Pop4	Dock8	-1.5122	-0.5168	-1.5847	0.2943	29.002	7.23E-08	F	7.73E-04
67	Lgi2	Spata31d1a	Arpp21	1.5064	0.5019	2.5012	0.4645	28.996	7.25E-08	F	7.73E-04
68	Lce3a	Rnf123	Slc44a5	-1.3278	-0.526	-1.4156	0.263	28.977	7.33E-08	F	7.73E-04
69	Olfr339	Lrrc61	Ncf2	-1.6347	-0.5304	-2.0293	0.3771	28.956	7.41E-08	F	7.73E-04
70	Def6	Fam3c	Cd86	1.3811	0.5104	2.2839	0.4247	28.919	7.55E-08	F	7.76E-04
71	Tjp3	Polr2i	Trem2	1.5891	0.5692	2.1291	0.3966	28.816	7.96E-08	F	7.93E-04
72	Csf1r	Milr1	H2-Ob	1.4035	0.5577	1.282	0.239	28.777	8.12E-08	F	7.93E-04
73	Mdn1	Cacna1s	Dock8	1.3427	0.5058	1.7017	0.3173	28.766	8.17E-08	F	7.93E-04
74	Akap10	Gm7337	Parvg	-1.347	-0.5126	-1.5573	0.2904	28.76	8.19E-08	F	7.93E-04
75	Snora26	Cblc	Prnp	-1.6031	-0.5355	-2.1009	0.3919	28.745	8.26E-08	F	7.93E-04
76	Mir181d	9130019O22Rik	Slc14a1	-1.5798	-0.5805	-2.08	0.3886	28.645	8.70E-08	F	8.19E-04
77	Clec4a2	Espn	Selplg	-1.6043	-0.5417	-1.8935	0.354	28.606	8.87E-08	F	8.19E-04
78	Azi2	Park7	Slc14a1	1.5767	0.5756	2.7397	0.5124	28.589	8.95E-08	F	8.19E-04
79	Psme3	Alkbh6	Slc14a1	-1.5742	-0.6181	-2.2853	0.4275	28.581	8.98E-08	F	8.19E-04
80	Ptcra	Prss50	Parvg	-1.4589	-0.5192	-2.1557	0.4037	28.522	9.26E-08	F	8.34E-04
81	Rabgap1	Mir335	Selplg	-1.4897	-0.5462	-2.0929	0.3926	28.426	9.73E-08	F	8.65E-04
82	Cbfa2t3	Sfxn5	Ezr	1.2484	0.5242	1.595	0.2999	28.279	1.05E-07	F	9.18E-04
83	Fgfrl1	Dok3	Cd180	-1.7521	-0.5488	-1.7505	0.3293	28.253	1.06E-07	S	9.18E-04
84	Aph1a	Zfp451	Lcp1	1.4926	0.5534	1.8556	0.3492	28.241	1.07E-07	F	9.18E-04
85	Higd1a	Eif1b	Cd180	1.4772	0.5521	1.991	0.375	28.186	1.10E-07	F	9.34E-04
86	Mpp4	Spata5	Selplg	-1.4602	-0.5272	-2.0123	0.3794	28.136	1.13E-07	F	9.47E-04
87	Anxa5	Tjp3	Ccl6	-1.5402	-0.5592	-1.8927	0.3572	28.075	1.17E-07	F	9.64E-04
88	Pim2	Tes	Slc15a3	1.383	0.5251	2.4624	0.4649	28.048	1.18E-07	F	9.64E-04
89	Olfr117	Defb20	Cst7	-1.3486	-0.5326	-2.0797	0.3928	28.035	1.19E-07	F	9.64E-04
90	Pdcd1	Tec	Dock8	1.3736	0.5757	1.8156	0.343	28.013	1.20E-07	F	9.64E-04
91	Olfr1044	Cyp2c68	Ccl3	1.6541	0.5302	3.5176	0.6651	27.976	1.23E-07	F	9.67E-04
92	Gck	4930583I09Rik	Slc11a1	-1.5431	-0.5435	-2.6901	0.5087	27.965	1.24E-07	F	9.67E-04
93	Slc38a11	Gpr125	Slc14a1	1.6169	0.556	2.1402	0.4054	27.871	1.30E-07	F	9.78E-04
94	Zfp367	4833413E03Rik	Gpr183	1.0974	0.5642	1.4361	0.272	27.869	1.30E-07	F	9.78E-04
95	D130046C19Rik	Rbm15b	Hpgds	1.3598	0.5228	1.4779	0.28	27.854	1.31E-07	F	9.78E-04
96	Chrna6	Ms4a8a	Gpr65	1.4842	0.5402	1.9936	0.3778	27.846	1.31E-07	F	9.78E-04
97	Hexb	Slamf6	Slc14a1	1.5072	0.5483	1.786	0.3385	27.833	1.32E-07	F	9.78E-04

98	Olfr39	Olig2	Slc44a5	-1.3821	-0.5226	-2.0618	0.3909	27.821	1.33E-07	F	9.78E-04
99	Nanog	Zcchc4	Slc14a1	-1.5406	-0.55	-2.8231	0.5355	27.796	1.35E-07	F	9.78E-04
100	MARCH7	:hr11:57877333-5788643	Trem3	1.5826	0.5093	2.1428	0.4066	27.774	1.36E-07	F	9.78E-04
101	Gpbar1	Pdcd5	Gsap	-1.6387	-0.6108	-2.6192	0.4972	27.756	1.38E-07	F	9.78E-04
102	Manba	Mlxip	Gpr65	-1.4611	-0.5281	-2.3495	0.4463	27.719	1.40E-07	F	9.78E-04
103	Pex12	Mir683-2	Slc11a1	1.1168	0.5084	1.1654	0.2214	27.698	1.42E-07	F	9.78E-04
104	Mat2b	Mrpl18	Gpr183	-1.6011	-0.534	-2.4947	0.4741	27.692	1.42E-07	F	9.78E-04
105	Clcn7	1810011O10Rik	Csf3r	1.4212	0.5557	2.0219	0.3844	27.673	1.44E-07	F	9.78E-04
106	Zbtb7c	Mill1	Gpr65	-1.3709	-0.5449	-1.9675	0.374	27.669	1.44E-07	F	9.78E-04
