# Supplementary material for: Three-way interaction model to trace the mechanisms involved in Alzheimer’s disease transgenic mice
Source: PLoS One. 2017 Sep 21;12(9):e0184697. doi: 10.1371/journal.pone.0184697 (PMC5608283; doi:10.1371/journal.pone.0184697)
Supplement: S3 Table — (PDF) [file pone.0184697.s003.pdf]

S3 Tables: Comparison of gene set enrichment analysis results in group1,2 and random groups.

For verifying that the large number of common terms in group 1(X3) and group2(X1, X2, X3) are not random, we extracted 30 random subsets from group 2, each subsets including 35 genes. After gene set enrichment analysis, very rare overlap was observed between group 2 and each of the random subsets. These results confirm that the large number of common terms in group 1 and 2 are not random.

R indicates Random groups; biological process, cellular component and pathway enrichment analysis results are available in Table 1,2 and 3 respectively.

S3 Tables: Comparison of gene set enrichment analysis results in group1,2 and random groups.

Table1: results of biological process enrichment analysis.

	GOID	GOTerm	p-value	fdr(BH)
	**Biological Process Enrichment Analysis for X1,X2,X3**			
	GO:0002286	T cell activation involved in immune response	3.71E-02	9.75E-02
	GO:1902106	negative regulation of leukocyte differentiation	3.09E-02	8.80E-02
	GO:0002763	positive regulation of myeloid leukocyte differentiation	1.09E-02	4.94E-02
	GO:0043409	negative regulation of MAPK cascade	3.57E-02	9.65E-02
	GO:0043410	positive regulation of MAPK cascade	2.73E-02	8.36E-02
	GO:0030593	neutrophil chemotaxis	1.08E-04	4.53E-03
	GO:0048245	eosinophil chemotaxis	4.87E-04	1.13E-02
**X1,X2,X3**	GO:0042098	T cell proliferation	6.81E-03	3.70E-02
	GO:0001779	natural killer cell differentiation	1.74E-03	1.83E-02
	GO:0002221	pattern recognition receptor signaling pathway	3.12E-03	2.44E-02
	GO:0045944	positive regulation of transcription from RNA polymerase II promoter	3.73E-02	9.75E-02
	GO:0070372	regulation of ERK1 and ERK2 cascade	6.61E-05	3.82E-03
	GO:0061097	regulation of protein tyrosine kinase activity	1.65E-02	6.20E-02
	GO:0050730	regulation of peptidyl-tyrosine phosphorylation	1.47E-02	5.94E-02
	GO:0070232	regulation of T cell apoptotic process	4.45E-03	2.94E-02
	**Biological Process Enrichment Analysis for X3**			
	GO:0070372	regulation of ERK1 and ERK2 cascade	2.6082E-08	2.55604E-07
**X3**	GO:0030593	neutrophil chemotaxis	4.17E-06	1.07E-05
	GO:0042098	T cell proliferation	9.53E-05	1.37E-04
	GO:0002221	pattern recognition receptor signaling pathway	3.48E-04	4.26E-04

S3 Tables: Comparison of gene set enrichment analysis results in group1,2 and random groups.

Table1: results of biological process enrichment analysis.

	**Biological Process Enrichment Analysis for Random Subsets**			
**R1**	GO:0070371	ERK1 and ERK2 cascade	3.03E-03	3.36E-03
	GO:0070372	regulation of ERK1 and ERK2 cascade	2.54E-03	3.17E-03
	GO:0070374	positive regulation of ERK1 and ERK2 cascade	9.15E-04	1.83E-03
**R2**	GO:0050730	regulation of peptidyl-tyrosine phosphorylation	2.49E-03	5.81E-03
	GO:0070371	ERK1 and ERK2 cascade	3.63E-03	3.63E-03
	GO:0070372	regulation of ERK1 and ERK2 cascade	3.04E-03	3.55E-03
**R3**		Without Enriched Biological Process		
**R4**		Without Enriched Biological Process		
**R5**		Without Enriched Biological Process		
**R6**		Without Enriched Biological Process		
**R7**		Without Enriched Biological Process		
**R8**		Without Enriched Biological Process		
**R9**		Without Enriched Biological Process		
**R10**		Without Enriched Biological Process		
**R11**		Without Enriched Biological Process		
**R12**		Without Enriched Biological Process		
	GO:0002221	pattern recognition receptor signaling pathway	3.10E-04	6.58E-04

S3 Tables: Comparison of gene set enrichment analysis results in group1,2 and random groups.

Table1: results of biological process enrichment analysis.

**R13**	GO:0002286	T cell activation involved in immune response	1.75E-04	5.95E-04
	GO:0070371	ERK1 and ERK2 cascade	2.39E-04	5.81E-04
	GO:0070372	regulation of ERK1 and ERK2 cascade	1.89E-04	5.35E-04
	GO:0070374	positive regulation of ERK1 and ERK2 cascade	4.79E-05	2.71E-04
	GO:0071902	positive regulation of protein serine/threonine kinase activity	3.42E-03	3.75E-03
**R14**		Without Enriched Biological Process		
**R15**	GO:0070588	calcium ion transmembrane transport	0.003736718	0.004670897
**R16**		Without Enriched Biological Process		
**R17**		Without Enriched Biological Process		
**R18**		Without Enriched Biological Process		
**R19**	GO:0070371	ERK1 and ERK2 cascade	3.32E-03	4.15E-03
	GO:0070372	regulation of ERK1 and ERK2 cascade	2.78E-03	4.64E-03
**R20**	GO:0070371	ERK1 and ERK2 cascade	3.63E-03	5.02E-03
	GO:0070372	regulation of ERK1 and ERK2 cascade	3.04E-03	4.57E-03
**R21**	GO:0045639	positive regulation of myeloid cell differentiation	2.08E-04	2.70E-03
**R22**	GO:0045619	regulation of lymphocyte differentiation	7.93E-04	1.47E-03
	GO:0001779	natural killer cell differentiation	5.49E-06	4.76E-05
	GO:0030217	T cell differentiation	2.92E-03	3.62E-03
	GO:0046631	alpha-beta T cell activation	4.69E-04	1.22E-03
**R23**		Without Enriched Biological Process		

S3 Tables: Comparison of gene set enrichment analysis results in group1,2 and random groups.

Table1: results of biological process enrichment analysis.

**R24**		Without Enriched Biological Process		
**R25**		Without Enriched Biological Process		
**R26**		Without Enriched Biological Process		
**R27**		Without Enriched Biological Process		
**R28**		Without Enriched Biological Process		
**R29**	GO:0070371	ERK1 and ERK2 cascade	3.63E-03	4.35E-03
	GO:0070372	regulation of ERK1 and ERK2 cascade	3.04E-03	4.06E-03
	GO:0070374	positive regulation of ERK1 and ERK2 cascade	1.10E-03	3.31E-03
**R30**		Without Enriched Biological Process		

S3 Tables: Comparison of gene set enrichment analysis results in group1,2 and random groups.

Table4: Random datasets

**R1**	Zbtb7c	Tnfaip8l3	Tmem26	Tgfbi	Syngr2	Snora26	Slc45a1	Sec24a	Sdsl	Pea15a	Olfr39	Olfr292
**R2**	Tyrp1	Trpm6	Trem3	Tnfaip8l3	Tmem26	Tgfbr1	Tgfbi	Sfxn5	Sdsl	Scnn1g	Rdh11	Olig2
**R3**	Zbtb7c	Txndc8	Tnfaip8l3	Tjp3	Spink2	Sned1	Slc39a4	Slc15a3	Sdsl	Rbm33	Olfr117	Ninl
**R4**	Tram1l1	Tceb1	Sqle	Spata31d1a	Smek3	Slc45a1	Rdh11	Prss50	Prokr1	Polr2i	Pglyrp1	Olfr292
**R5**	Zfp451	Tyrp1	Tmprss11g	Tgfbi	Syngr2	Spsb4	Slc45a1	Slc38a11	Slc11a1	Sh3pxd2b	Selplg	Rbm15b
**R6**	Zfp930	Tyrp1	Trem2	Tram1l1	Tmem79	Tmem26	Tlr4	Slc39a4	Slc11a1	Sdsl	Runx1	Ptcra
**R7**	Trem3	Tmprss11g	Tjp3	Sh3pxd2b	Qtrt1	Polr2i	Pdcd1	Parvg	Olfr1044	Nxnl1	Ncf2	Mknk2
**R8**	Zfp451	Zfp367	Vps51	Tram1l1	Tnfaip8l3	Tgfbr1	Tgfbi	Spink2	Selplg	Sdsl	Ptcra	Psme3
**R9**	Zfp507	Zfp451	Zfp367	Serf1	Sec24a	Rela	Rbm15b	Rabgap1	Ptcra	Pop4	Pdcd1	Olig2
**R10**	Zfp507	Zfp408	Vps51	Vmn2r-ps57	Spata31d1a	Slc14a1	Serf1	Rbm33	Psg20	Prokr1	Prnp	Pdcd1
**R11**	Zfp408	Zfp367	Zcchc4	Tram1l1	Tgfbi	Snora26	Sec24a	Rbm33	Pglyrp1	Pde3a	Olfr292	Nxnl1
**R12**	Tyrp1	Trem3	Tram1l1	Tnfaip8l3	Tgfbi	Slc38a11	Sfxn5	Pop4	Pim2	Pex12	Parvg	Olfr39
**R13**	Zfp408	Tmem79	Tlr4	Syngr2	Spsb4	Smek3	Slc44a5	Slamf6	Sh3pxd2b	Rbm33	Ptcra	Pglyrp1
**R14**	Zcchc4	Zbtb7c	Tmprss11g	Tlr4	Tes	Sqle	Slc44a5	Slc35e4	Slc15a3	Sh3pxd2b	Selplg	Prss50
**R15**	Zfp408	Trpm6	Trem2	Tnfaip8l3	Tjp3	Sqle	Slc45a1	Slc44a5	Runx1	Prokr1	Pgc	Pde3a
**R16**	Zfp930	Zfp451	Trem2	Tmprss11g	Smek3	Slc45a1	Slc35e4	Sdsl	Rdh11	Rbm15b	Qtrt1	Psme3
**R17**	Zfp930	Zfp367	Zbtb7c	Tes	Spata31d1a	Slc39a4	Slamf6	Sh3pxd2b	Sfxn5	Sec24a	Runx1	Pglyrp1
**R18**	Zbtb7c	Tmem79	Spata31d1a	Slc35e4	Sh3pxd2b	Sdsl	Rasd2	Rabgap1	Psme3	Park7	Olfr346	Olfr1044
**R19**	Zfp408	Zfp367	Vps51	Tm6sf2	Syngr2	Smek3	Sfxn5	Serf1	Rnf123	Rdh11	Rabgap1	Ptcra
**R20**	Zfp451	Trpm6	Tram1l1	Slc44a5	Rela	Pglyrp1	Pgc	Olfr339	Olfr1044	Ninl	Mrpl18	Mir141
**R21**	Zfp408	Tjp3	Spata5	Slamf6	Rela	Rbm15b	Rabgap1	Pim2	Olfr304	Nxnl1	Nckap1l	Nanp
**R22**	Vps51	Tnfaip8l3	Tmem79	Tgfbi	Tes	Tec	Sqle	Spink2	Snora26	Slc15a3	Slamf6	Rdh11
**R23**	Tnfaip8l3	Tmprss11g	Tm6sf2	Tgfbi	Tceb1	Spink2	Sned1	Smek3	Slc11a1	Rnf123	Qtrt1	Polr2i
**R24**	Zfp930	Tram1l1	Slamf6	Sh3pxd2b	Sfxn5	Sdsl	Scnn1g	Psme3	Psd3	Prnp	Pex12	Olfr339
**R25**	Vmn2r-ps57	Tyrp1	Tram1l1	Tjp3	Slc14a1	Slamf6	Serf1	Rnf123	Psg20	Psd3	Prnp	Polr2i
**R26**	Zfp367	Txndc8	Tmprss11g	Tmem79	Slc45a1	Slamf6	Sh3pxd2b	Serf1	Scnn1g	Runx1	Rbm15b	Pdcd1
**R27**	Zfp930	Tec	Tceb1	Smek3	Slc35e4	Slc14a1	Sdsl	Polr2i	Pim2	Pex12	Pdcd5	Park7
**R28**	Zfp930	Zfp507	Zfp408	Vmn2r-ps57	Tram1l1	Tjp3	Tes	Syngr2	Slc44a5	Slc35e4	Ptcra	Prokr1
**R29**	Zfp930	Zfp367	Tyrp1	Trem2	Tjp3	Syngr2	Slc11a1	Sfxn5	Psg20	Pop4	Polr2i	Pea15a
**R30**	Zfp451	Zfp367	Vmn2r-ps57	Tmprss11g	Tm6sf2	Sned1	Slc45a1	Runx1	Rnf123	Qtrt1	Psg20	Pglyrp1

S3 Tables: Comparison of gene set enrichment analysis results in group1,2 and random groups.

Table4: Random datasets

**R1**	Ncoa4	Nanog	Mroh4	Mmp8	Mir144	Milr1	Kcnq4	Itpripl2	Igfbpl1	Fopnl	Fam3c	Eif5	E330034G19Rik
**R2**	Olfr292	Olfr117	Nanp	Ms4a8a	Mrpl18	Lcp1	Lamb1	Klf6	Kcnq4	Il15	Htr1a	Fam3c	Ezr
**R3**	Nanp	Mmp8	Mlxip	Mknk2	Mir683-2	Mir21a	Mir181d	Mir144	Mir141	Kcnq4	Irf8	Hrct1	H2-Ob
**R4**	Olfr172	Olfr146	Nsdhl	Nanog	Mrpl18	Mpp4	Mir141	Milr1	Manba	Kcnq4	Gpr65	Gpr125	Fam120b
**R5**	Olfr339	Olfr1044	Nup98	Ncf2	Mrpl18	Mir683-2	Mir335	Mat2b	Manba	Lce3a	Itpripl2	Irf8	Inpp5b
**R6**	Prnp	Pgc	Pdcd1	Parvg	Olfr146	Ncoa4	Ms4a8a	Mir683-2	Ldlr	Kcnq4	Irf8	Hirip3	Gm7337
**R7**	Mir683-2	Mir144	Mir141	Milr1	Mill1	Inpp5b	Hpgds	Hexb	H2-Ob	Gsap	Gpr125	Gpbar1	Gm839
**R8**	Pea15a	Nup98	Ncf2	Mknk2	Mdn1	Manba	Gsap	Gm839	Flcn	Eif5	Ctsd	Cst7	Cldn9
**R9**	Olfr39	Olfr346	Nckap1l	Ncf2	Nanog	Ms4a8a	Mir683-2	Milr1	Mat2b	Hyou1	H2-Ob	Fyb	Fam217a
**R10**	Olig2	Ncoa4	Nanp	Mknk2	Mir335	Mdn1	MARCH7	Hyou1	Hrct1	H2-Ob	Gpr125	Gpbar1	Fyb
**R11**	Ninl	Nanp	Mlxip	Mir335	Mir181d	Mir144	Mdn1	Letm1	Kcnq4	H2-Ob	Gpr183	Gm839	Fam217a
**R12**	Olfr292	Nxnl1	Ncf2	Ms4a8a	Mroh4	Mpeg1	Letm1	Ldlr	Lamb1	Inpp5b	Hyou1	Flcn	Eif5
**R13**	Nsdhl	Ncoa4	Mmp8	Mir683-2	Mir21a	Lrrc61	Lcp1	Klf12	Hpgds	Hirip3	Higd1a	Gpr125	Gm10620
**R14**	Pop4	Pim2	Pex12	Pdcd1	Park7	Olfr555	Mroh4	Mir181d	Klf6	Igfbpl1	Hexb	Gsap	Gm839
**R15**	Pdcd1	Osbpl6	Olfr346	Olfr117	Nptx1	Mroh4	Mlxip	Mknk2	Mill1	Ly86	Fabp1	Eif1b	Dok3
**R16**	Osbpl6	Olfr292	Nsdhl	Ms4a8a	Mrpl18	Mroh4	Mpp4	Mir335	Ldlr	Inpp5b	Higd1a	Gm7337	Fyb
**R17**	Park7	Nxnl1	Nsdhl	Ninl	Nanp	Mir683-2	Mir141	MARCH7	Klf6	Irf8	Hrct1	Fam120b	Evc2
**R18**	Mmp8	Mir181d	Mir144	Ly86	Klf12	Il15	Hrct1	Hexb	Gm10620	Flcn	Eya3	Evc2	E230016K23Rik
**R19**	Prss50	Pop4	Pdcd1	Parvg	Olfr555	Olfr346	Olfr146	Mpp4	Mir335	Mir21a	Lce3a	Lamb1	Inpp5b
**R20**	Lrrc61	Letm1	Itpripl2	Irf8	Higd1a	Hexb	Gpr125	Gpbar1	Gm10620	Fopnl	Flcn	Fam120b	E230016K23Rik
**R21**	Nanog	Mrpl18	Letm1	Ldlr	Klf6	Klf14	Klf12	Irf8	Htr1a	Higd1a	Hexb	Flcn	Fam3c
**R22**	Rasd2	Ptcra	Pglyrp1	Pea15a	Nsdhl	Nckap1l	Mir144	Ldlr	Il15	Higd1a	Gpr65	Gm7337	Eya3
**R23**	Pdcd1	Olfr346	Olfr339	Mroh4	Mpp4	MARCH7	Manba	Letm1	Klf14	Kcnq4	Irf8	Fabp1	Eif5
**R24**	Olfr172	Mir683-2	Mir21a	Mir181d	MARCH7	Letm1	Lcp1	Igfbpl1	Hpgds	Hexb	H2-Ob	Gpr65	Gpr125
**R25**	Olfr39	Olfr146	Nanp	MARCH7	Ldlr	Hirip3	Higd1a	Hexb	Gpbar1	Eif5	Defb20	D130046C19Rik	D030056L22Rik
**R26**	Park7	Olfr292	Olfr172	Nptx1	Ncf2	Nanog	Ms4a8a	Mrpl18	Lgi2	Lce3a	Inpp5b	Gck	Fyb
**R27**	Olfr117	Milr1	Mdn1	Manba	Lgi2	Klf6	Klf12	Hyou1	Gpr65	Gpr183	Gpbar1	Def6	Clcn7
**R28**	Prnp	Pop4	Pglyrp1	Pex12	Pde3a	Olfr339	Olfr117	Olfr1044	Ndp	Mpp4	Mmp8	Mir448	Lamb1
**R29**	Olfr346	Mir448	Mir21a	Mdn1	Manba	Kcnq4	Itpripl2	Hirip3	Hexb	Gck	Fopnl	Fabp1	Eif1b
**R30**	Pex12	Pdcd1	Olfr555	Olfr146	Ndp	Mroh4	Mpeg1	Mir448	Mir335	Mir144	Milr1	Mill1	Klf12

S3 Tables: Comparison of gene set enrichment analysis results in group1,2 and random groups.

Table4: Random datasets

**R1**	Dock8	chr16:91712003-91712125	chr11:57877333-57886439	Cebpz	Ccr1l1
**R2**	Eya3	Eif1b	E330034G19Rik	Def6	Csf1r
**R3**	Fam175a	Fabp1	D030056L22Rik	Cacna1s	Aph1a
**R4**	Eif1b	Cyp2c68	Csf1r	chr16:91712003-91712125	Atp9a
**R5**	Gpr65	Gpr125	Evc2	Def6	Ccr1l1
**R6**	Espn	Eif1b	E330034G19Rik	Cst7	Clcn7
**R7**	Fgfrl1	Fam175a	E330034G19Rik	D030056L22Rik	chr11:57877333-57886439
**R8**	chr11:57877333-57886439	Cd86	Ccr1l1	Cacna1s	Azi2
**R9**	Eif5	E230016K23Rik	Cyp2c68	Chrna6	Ccl3
**R10**	Espn	Dock8	Cyp2j13	Cadm4	Cacna1s
**R11**	Defb20	chr16:91712003-91712125	Ccl6	Cadm4	Atp9a
**R12**	E330034G19Rik	Clec7a	Cldn9	chr16:91712003-91712125	Cd48
**R13**	D130046C19Rik	Csf1r	Cops7b	Cd86	Ccl6
**R14**	Gm7337	Dock8	Clec7a	chr16:91712003-91712125	Bbs5
**R15**	Cyp2j13	Ctsd	Clcn7	chr16:91712003-91712125	Cacna1s
**R16**	Flcn	Eif5	Eif1b	E230016K23Rik	Ctsd
**R17**	Defb20	Clec4a2	Cldn9	Clcn7	Ccdc108
**R18**	Dcaf8	D130046C19Rik	Clec7a	Cldn9	Chrna6
**R19**	Gck	Fyb	Ezr	Cyp2j13	Csf1r
**R20**	Cyp2j13	Csf1r	Clec4a2	Clcn7	Ccl3
**R21**	E230016K23Rik	Defb20	Dcaf8	Csf1r	Clcn7
**R22**	Evc2	Defb20	Cox8c	Cd180	Bbs5
**R23**	Eif1b	Dok3	Clec7a	Cd86	Cd48
**R24**	Gck	Cops7b	Chrna6	Cfap20	Ap2s1
**R25**	Ctsd	Clec7a	Cebpz	Aph1a	Anxa5
**R26**	E230016K23Rik	Dok3	Cst7	Cops7b	Ccdc108
**R27**	Cebpz	Ccr1l1	Art2a-ps	App	Ap2s1
**R28**	Irf8	Flcn	Fabp1	D130046C19Rik	Csf3r
**R29**	E230016K23Rik	Cyp2j13	Cops7b	Chrna6	Cfap20
**R30**	H2-Ob	Gsap	Gpr125	Gm10620	Eif5

S3 Tables: Comparison of gene set enrichment analysis results in group1,2 and random groups.

Table4: Random datasets

**R1**	Ccl9	Ccl6	Azi2	Alkbh6	Abcf2
**R2**	Cox8c	Cblc	Abcf2	9230110C19Rik	5430411C19Rik
**R3**	Alpk1	Acbd6	9130019O22Rik	2900060B14Rik	1700080E11Rik
**R4**	Arpp21	Aph1a	Acvr1b	9130019O22Rik	4930583I09Rik
**R5**	Ccl9	Cadm4	App	Abcf2	4833413E03Rik
**R6**	Alkbh6	9230110C19Rik	5430411C19Rik	4833413E03Rik	1810011O10Rik
**R7**	Ccdc108	App	Ap2s1	Akap10	4833413E03Rik
**R8**	Aph1a	Aldoc	Abcf2	4930426L09Rik	1500011B03Rik
**R9**	Cblc	Anxa5	Alkbh6	4833413E03Rik	2900060B14Rik
**R10**	Bbs5	Azi2	Atp9a	App	Akap10
**R11**	Arpp21	Alox12e	9130019O22Rik	4930583I09Rik	4833413E03Rik
**R12**	Ccr1l1	Bbs5	Aph1a	Alkbh6	4930583I09Rik
**R13**	Ccl3	Ccdc108	Atp9a	9130019O22Rik	4930426L09Rik
**R14**	Art2a-ps	Alox12e	Akap10	5430411C19Rik	1500011B03Rik
**R15**	Anxa5	Alox12e	Acbd6	9230110C19Rik	1500011B03Rik
**R16**	Cd180	Ccr1l1	Cbfa2t3	Bbs5	Alpk1
**R17**	Aph1a	Alox12e	Acbd6	Abcf2	1500011B03Rik
**R18**	chr16:91712003-91712125	Cebpz	Bcl2a1c	Ap2s1	4930426L09Rik
**R19**	Cops7b	Cldn9	Chrna6	Ccl9	Bbs5
**R20**	Cblc	Cbfa2t3	Blmh	Ap2s1	Abcf2
**R21**	Cebpz	Cd48	Cadm4	Acvr1b	4833413E03Rik
**R22**	Arpp21	Alpk1	9130019O22Rik	5430411C19Rik	1500011B03Rik
**R23**	Ccdc108	Blmh	Alox12e	Akap10	9130019O22Rik
**R24**	Alkbh6	4930583I09Rik	4833413E03Rik	1700080E11Rik	1500011B03Rik
**R25**	Alpk1	Akap10	Abcf2	4930583I09Rik	4930426L09Rik
**R26**	Bbs5	Apom	Akap10	Acbd6	4833413E03Rik
**R27**	Acvr1b	9230110C19Rik	4930583I09Rik	4833413E03Rik	1700080E11Rik
**R28**	Ccdc108	Ap2s1	Alox12e	Aldoc	2900060B14Rik
**R29**	Ccl9	Ccl3	Cadm4	Bbs5	Alox12e
**R30**	Dock8	Cops7b	Ccr1l1	App	9230110C19Rik
