# Supplementary material for: Three-way interaction model to trace the mechanisms involved in Alzheimer’s disease transgenic mice
Source: PLoS One. 2017 Sep 21;12(9):e0184697. doi: 10.1371/journal.pone.0184697 (PMC5608283; doi:10.1371/journal.pone.0184697)
Supplement: S4 Table — (PDF) [file pone.0184697.s004.pdf]

S4 Tables

Table 1. Gene set enrichment analysis results based on biological process

Biological Process Enrichment Analysis for X3				
**GOID**	**GOTerm**	**GOLevels**	***p*-value**	**FDR (BH)**
GO:0000165	MAPK cascade	[4, 5, 6, 8, 9]	5.66E-06	1.32E-05
GO:0001816	cytokine production	[3]	1.65E-05	3.36E-05
GO:0001818	negative regulation of cytokine production	[3, 4, 5]	1.06E-04	1.44E-04
GO:0001819	positive regulation of cytokine production	[3, 4, 5]	0.001171	0.001247
GO:0002221	pattern recognition receptor signaling pathway	[5, 7, 8, 9, 10]	3.48E-04	4.26E-04
GO:0002274	myeloid leukocyte activation	[3, 5]	2.67E-06	8.19E-06
GO:0002285	lymphocyte activation involved in immune response	[3, 4, 5, 6]	6.11E-08	3.74E-07
GO:0002286	T cell activation involved in immune response	[3, 4, 5, 6, 7, 9, 10]	1.84E-04	2.31E-04
GO:0002366	leukocyte activation involved in immune response	[3, 4, 5]	4.90E-10	1.20E-08
GO:0002367	cytokine production involved in immune response	[3, 4]	9.60E-05	1.34E-04
GO:0002521	leukocyte differentiation	[5, 6, 7, 8]	6.08E-04	6.77E-04
GO:0002577	regulation of antigen processing and presentation	[3, 4]	2.80E-06	8.06E-06
GO:0002682	regulation of immune system process	[2, 3]	4.54E-08	3.71E-07
GO:0002683	negative regulation of immune system process	[2, 3, 4]	1.22E-05	2.59E-05
GO:0002684	positive regulation of immune system process	[2, 3, 4]	6.67E-08	3.63E-07
GO:0002757	immune response-activating signal transduction	[3, 5, 6, 7]	2.30E-05	4.34E-05
GO:0006935	chemotaxis	[3, 4]	5.44E-04	6.20E-04
GO:0006954	inflammatory response	[4]	1.76E-05	3.45E-05
GO:0007159	leukocyte cell-cell adhesion	[4, 5]	2.69E-07	1.10E-06
GO:0009620	response to fungus	[3, 5]	2.53E-05	4.43E-05
GO:0019221	cytokine-mediated signaling pathway	[5, 6]	0.001158	0.001261
GO:0030099	myeloid cell differentiation	[5, 6, 7, 8]	0.001209	0.00126
GO:0030593	neutrophil chemotaxis	[5, 6, 7, 8, 9, 10]	4.17E-06	1.07E-05
GO:0031349	positive regulation of defense response	[3, 4, 5, 6]	2.44E-05	4.42E-05
GO:0032623	interleukin-2 production	[4]	9.39E-07	3.29E-06
GO:0032649	regulation of interferon-gamma production	[4, 5]	7.24E-06	1.61E-05
GO:0033993	response to lipid	[4]	3.13E-06	8.53E-06
GO:0034097	response to cytokine	[4]	3.07E-05	5.01E-05
GO:0034341	response to interferon-gamma	[4, 5]	4.38E-06	1.07E-05
GO:0042098	T cell proliferation	[5, 6, 7, 9, 10]	9.53E-05	1.37E-04
GO:0042110	T cell activation	[4, 6, 8, 9]	2.85E-05	4.82E-05
GO:0042113	B cell activation	[4, 6]	3.50E-04	4.18E-04
GO:0045087	innate immune response	[3, 4]	6.70E-09	8.20E-08
GO:0045321	leukocyte activation	[2, 4]	3.42E-10	1.68E-08
GO:0046649	lymphocyte activation	[3, 5]	2.14E-07	9.53E-07
GO:0046651	lymphocyte proliferation	[4, 5, 6]	1.13E-07	5.54E-07
GO:0046916	cellular transition metal ion homeostasis	[8, 9]	1.54E-04	1.98E-04
GO:0050663	cytokine secretion	[4, 5, 6, 7, 8]	5.86E-05	8.70E-05
GO:0050670	regulation of lymphocyte proliferation	[5, 6, 7]	1.33E-04	1.76E-04
GO:0050727	regulation of inflammatory response	[4, 5, 6]	0.003642	0.003642
GO:0050778	positive regulation of immune response	[3, 4, 5]	3.83E-05	5.86E-05
GO:0050829	defense response to Gram-negative bacterium	[5, 6, 7]	3.51E-07	1.32E-06
GO:0050848	regulation of calcium-mediated signaling	[5, 6, 7, 8]	3.08E-05	4.87E-05
GO:0050900	leukocyte migration	[2, 4, 5, 6]	1.45E-06	4.74E-06
GO:0051017	actin filament bundle assembly	[4, 7, 8]	4.41E-04	5.14E-04
GO:0051402	neuron apoptotic process	[5, 7]	0.003233	0.003301
GO:0051707	response to other organism	[2, 4]	4.64E-08	3.25E-07
GO:0070372	regulation of ERK1 and ERK2 cascade	[6, 7, 8, 9, 10, 11]	2.61E-08	2.56E-07
GO:0071216	cellular response to biotic stimulus	[3]	4.64E-09	7.58E-08
GO:0071222	cellular response to lipopolysaccharide	[5, 6, 8]	6.11E-08	3.74E-07
**Biological Process Enrichment Analysis for X1,X2, X3**				
**GOID**	**GOTerm**	**GOLevels**	***p*-value**	**FDR (BH)**
GO:0001775	cell activation	[3]	1.37E-04	0.005283
GO:0001779	natural killer cell differentiation	[5, 7, 8, 9, 10]	0.001744	0.018312
GO:0001816	cytokine production	[3]	7.46E-04	0.012309

S4 Tables

Table 1. Gene set enrichment analysis results based on biological process

GO:0001817	regulation of cytokine production	[3, 4]	0.001893	0.01822
GO:0001818	negative regulation of cytokine production	[3, 4, 5]	0.007768	0.039438
GO:0001819	positive regulation of cytokine production	[3, 4, 5]	0.006784	0.03731
GO:0001942	hair follicle development	[3, 5, 6, 7, 8]	0.001774	0.017819
GO:0002221	pattern recognition receptor signaling pathway	[5, 7, 8, 9, 10]	0.003121	0.02444
GO:0002274	myeloid leukocyte activation	[3, 5]	8.41E-04	0.012529
GO:0002285	lymphocyte activation involved in immune response	[3, 4, 5, 6]	5.50E-04	0.011545
GO:0002286	T cell activation involved in immune response	[3, 4, 5, 6, 7, 9, 10]	0.037126	0.097456
GO:0002312	B cell activation involved in immune response	[3, 4, 5, 6, 7]	0.015155	0.059845
GO:0002366	leukocyte activation involved in immune response	[3, 4, 5]	3.51E-05	0.004053
GO:0002367	cytokine production involved in immune response	[3, 4]	0.021015	0.071917
GO:0002444	myeloid leukocyte mediated immunity	[4]	0.026131	0.082687
GO:0002520	immune system development	[2, 4, 5]	0.001934	0.017872
GO:0002521	leukocyte differentiation	[5, 6, 7, 8]	0.004144	0.028156
GO:0002548	monocyte chemotaxis	[4, 6, 7, 8]	7.02E-04	0.012472
GO:0002573	myeloid leukocyte differentiation	[6, 7, 8, 9]	0.036275	0.096873
GO:0002577	regulation of antigen processing and presentation	[3, 4]	3.65E-05	0.003373
GO:0002643	regulation of tolerance induction	[3, 4, 5, 6, 7]	3.45E-04	0.008868
GO:0002682	regulation of immune system process	[2, 3]	1.13E-05	0.0052
GO:0002683	negative regulation of immune system process	[2, 3, 4]	0.003465	0.025823
GO:0002684	positive regulation of immune system process	[2, 3, 4]	1.44E-04	0.005134
GO:0002690	positive regulation of leukocyte chemotaxis	[4, 5, 6, 7, 8, 9]	0.027974	0.084472
GO:0002697	regulation of immune effector process	[3, 4]	0.030833	0.088478
GO:0002757	immune response-activating signal transduction	[3, 5, 6, 7]	7.35E-04	0.012583
GO:0002763	positive regulation of myeloid leukocyte differentiation	[5, 6, 7, 8, 9, 10, 11]	0.010914	0.049432
GO:0002831	regulation of response to biotic stimulus	[3, 4]	0.002634	0.021728
GO:0002833	positive regulation of response to biotic stimulus	[3, 4, 5]	0.005472	0.03283
GO:0006066	alcohol metabolic process	[4]	0.027144	0.083604
GO:0006468	protein phosphorylation	[6, 7]	0.035933	0.096519
GO:0006690	icosanoid metabolic process	[4, 6, 7]	0.014673	0.058948
GO:0006810	transport	[3]	0.033409	0.092425
GO:0006811	ion transport	[4, 5]	0.035011	0.09571
GO:0006820	anion transport	[5, 6]	0.014241	0.058746
GO:0006909	phagocytosis	[4, 5, 6]	0.017691	0.065386
GO:0006952	defense response	[3]	2.15E-04	0.006197
GO:0006954	inflammatory response	[4]	0.002275	0.020209
GO:0007166	cell surface receptor signaling pathway	[4, 5]	0.010048	0.046423
GO:0007586	digestion	[3]	0.004409	0.029524
GO:0008203	cholesterol metabolic process	[6]	0.019857	0.070031
GO:0008285	negative regulation of cell proliferation	[3, 4, 5]	0.015026	0.059844
GO:0008347	glial cell migration	[4, 5, 6, 7, 8]	0.004777	0.03065
GO:0008637	apoptotic mitochondrial changes	[5, 6]	0.014673	0.058948
GO:0009119	ribonucleoside metabolic process	[5, 6, 7]	0.008653	0.042081
GO:0009166	nucleotide catabolic process	[5, 6, 7, 8]	0.005751	0.03363
GO:0009611	response to wounding	[3]	0.015874	0.061116
GO:0009617	response to bacterium	[3, 5]	6.95E-04	0.012848
GO:0009620	response to fungus	[3, 5]	0.006225	0.03595
GO:0009893	positive regulation of metabolic process	[2, 3, 4]	0.008366	0.041561
GO:0009966	regulation of signal transduction	[3, 4, 5]	0.025154	0.080146
GO:0010604	positive regulation of macromolecule metabolic process	[3, 4, 5]	0.008599	0.042264
GO:0010647	positive regulation of cell communication	[3, 4, 5]	0.032778	0.091779
GO:0010876	lipid localization	[3]	0.016054	0.061298
GO:0012501	programmed cell death	[4]	0.003271	0.024772
GO:0015696	ammonium transport	[5, 7, 8]	0.026131	0.082687
GO:0015844	monoamine transport	[4, 5]	0.022651	0.075286
GO:0015914	phospholipid transport	[5, 6, 7, 8]	0.009849	0.045964
GO:0016052	carbohydrate catabolic process	[4]	0.007112	0.038204

S4 Tables

Table 1. Gene set enrichment analysis results based on biological process

GO:0016310	phosphorylation	[5]	0.038464	0.099277
GO:0019220	regulation of phosphate metabolic process	[5, 6]	0.026869	0.083312
GO:0019221	cytokine-mediated signaling pathway	[5, 6]	0.00666	0.037074
GO:0019932	second-messenger-mediated signaling	[5, 6]	0.033636	0.0925
GO:0023014	signal transduction by protein phosphorylation	[3, 4, 5, 7, 8]	0.013415	0.057384
GO:0023056	positive regulation of signaling	[2, 3, 4]	0.029033	0.085433
GO:0030099	myeloid cell differentiation	[5, 6, 7, 8]	0.007164	0.038041
GO:0030193	regulation of blood coagulation	[4, 5, 6, 7]	0.026131	0.082687
GO:0030593	neutrophil chemotaxis	[5, 6, 7, 8, 9, 10]	1.08E-04	0.004529
GO:0030595	leukocyte chemotaxis	[3, 5, 6, 7]	0.00116	0.014881
GO:0031325	positive regulation of cellular metabolic process	[3, 4, 5]	0.001457	0.016422
GO:0031331	positive regulation of cellular catabolic process	[4, 5, 6]	0.026363	0.082856
GO:0031347	regulation of defense response	[4, 5]	9.20E-04	0.012143
GO:0031349	positive regulation of defense response	[3, 4, 5, 6]	8.01E-04	0.012759
GO:0031401	positive regulation of protein modification process	[5, 6, 7, 8]	0.013471	0.057097
GO:0031532	actin cytoskeleton reorganization	[5, 6]	0.005751	0.03363
GO:0031663	lipopolysaccharide-mediated signaling pathway	[5, 6, 7, 9]	7.24E-05	0.003715
GO:0032101	regulation of response to external stimulus	[3, 4]	0.001764	0.018112
GO:0032103	positive regulation of response to external stimulus	[3, 4, 5]	0.003147	0.024231
GO:0032270	positive regulation of cellular protein metabolic process	[4, 5, 6, 7]	0.002528	0.021237
GO:0032496	response to lipopolysaccharide	[4, 5, 7]	0.001222	0.015257
GO:0032609	interferon-gamma production	[4]	5.87E-05	0.003873
GO:0032623	interleukin-2 production	[4]	0.001484	0.016328
GO:0032660	regulation of interleukin-17 production	[4, 5]	2.45E-05	0.003769
GO:0032689	negative regulation of interferon-gamma production	[4, 5, 6]	0.001411	0.016294
GO:0032729	positive regulation of interferon-gamma production	[4, 5, 6]	0.002736	0.02218
GO:0032735	positive regulation of interleukin-12 production	[4, 5, 6]	0.00233	0.020314
GO:0033238	regulation of cellular amine metabolic process	[4, 5, 6]	0.004139	0.028537
GO:0033559	unsaturated fatty acid metabolic process	[5, 6, 8, 9]	0.018741	0.067117
GO:0034097	response to cytokine	[4]	0.011598	0.051524
GO:0034341	response to interferon-gamma	[4, 5]	9.14E-04	0.012414
GO:0035987	endodermal cell differentiation	[5, 6, 7, 8, 9]	0.006624	0.037322
GO:0042063	gliogenesis	[6, 7]	0.030311	0.088074
GO:0042098	T cell proliferation	[5, 6, 7, 9, 10]	0.006814	0.037036
GO:0042110	T cell activation	[4, 6, 8, 9]	0.024641	0.079055
GO:0042325	regulation of phosphorylation	[6, 7]	0.013823	0.057535
GO:0042742	defense response to bacterium	[4, 5, 6]	0.005433	0.033029
GO:0042981	regulation of apoptotic process	[5, 6]	0.013521	0.056787
GO:0043065	positive regulation of apoptotic process	[5, 6, 7]	0.033073	0.092047
GO:0043066	negative regulation of apoptotic process	[5, 6, 7]	0.005061	0.031594
GO:0043085	positive regulation of catalytic activity	[3, 4, 5]	0.030942	0.087701
GO:0043409	negative regulation of MAPK cascade	[5, 6, 7, 8, 9, 10, 11]	0.035716	0.096497
GO:0043410	positive regulation of MAPK cascade	[5, 6, 7, 8, 9, 10, 11]	0.027337	0.08364
GO:0043467	regulation of generation of precursor metabolites and energy	[4, 5]	0.01045	0.047799
GO:0043491	protein kinase B signaling	[5, 6]	0.021304	0.07237
GO:0043900	regulation of multi-organism process	[2, 3]	5.22E-04	0.01148
GO:0043902	positive regulation of multi-organism process	[2, 3, 4]	0.004409	0.029524
GO:0044765	single-organism transport	[3, 4]	0.030207	0.088326
GO:0045087	innate immune response	[3, 4]	1.90E-05	0.004392
GO:0045321	leukocyte activation	[2, 4]	1.52E-04	0.005026
GO:0045576	mast cell activation	[4, 6]	0.001805	0.017745
GO:0045637	regulation of myeloid cell differentiation	[4, 5, 6, 7, 8, 9]	0.006457	0.036831
GO:0045937	positive regulation of phosphate metabolic process	[5, 6, 7]	0.013102	0.05657
GO:0045944	positive regulation of transcription from RNA polymerase II p	[6, 7, 8, 9, 10, 11, 12]	0.037344	0.097473
GO:0046434	organophosphate catabolic process	[4, 5]	0.01207	0.053108
GO:0046496	nicotinamide nucleotide metabolic process	[6, 7, 8, 9]	0.007575	0.038885
GO:0046649	lymphocyte activation	[3, 5]	0.003938	0.02799

S4 Tables

Table 1. Gene set enrichment analysis results based on biological process

GO:0046651	lymphocyte proliferation	[4, 5, 6]	8.84E-05	0.004083
GO:0046847	filopodium assembly	[5]	0.007913	0.039737
GO:0048245	eosinophil chemotaxis	[5, 6, 7, 8, 9, 10]	4.87E-04	0.011255
GO:0048247	lymphocyte chemotaxis	[4, 6, 7, 8]	0.005117	0.03152
GO:0048469	cell maturation	[4, 5, 6]	0.018994	0.067501
GO:0048518	positive regulation of biological process	[1, 2, 3]	0.007425	0.038982
GO:0048522	positive regulation of cellular process	[2, 3, 4]	0.011539	0.051757
GO:0048583	regulation of response to stimulus	[2, 3]	0.001281	0.015173
GO:0048584	positive regulation of response to stimulus	[2, 3, 4]	1.71E-04	0.00527
GO:0050663	cytokine secretion	[4, 5, 6, 7, 8]	0.015268	0.059778
GO:0050691	regulation of defense response to virus by host	[4, 5, 6, 7, 8]	0.022828	0.075334
GO:0050727	regulation of inflammatory response	[4, 5, 6]	0.00871	0.041485
GO:0050730	regulation of peptidyl-tyrosine phosphorylation	[7, 8, 9, 10]	0.014653	0.059382
GO:0050776	regulation of immune response	[3, 4]	0.004075	0.028524
GO:0050829	defense response to Gram-negative bacterium	[5, 6, 7]	5.91E-04	0.01138
GO:0050848	regulation of calcium-mediated signaling	[5, 6, 7, 8]	0.007468	0.038767
GO:0050851	antigen receptor-mediated signaling pathway	[5, 7, 8, 9]	0.028127	0.083836
GO:0050865	regulation of cell activation	[3, 4]	0.038415	0.099707
GO:0050900	leukocyte migration	[2, 4, 5, 6]	8.71E-04	0.012578
GO:0051017	actin filament bundle assembly	[4, 7, 8]	0.020431	0.070441
GO:0051241	negative regulation of multicellular organismal process	[2, 3, 4]	0.022296	0.074642
GO:0051246	regulation of protein metabolic process	[4, 5]	0.018171	0.066627
GO:0051247	positive regulation of protein metabolic process	[4, 5, 6]	0.001595	0.017133
GO:0051272	positive regulation of cellular component movement	[3, 4, 5]	0.035188	0.095629
GO:0051707	response to other organism	[2, 4]	3.32E-04	0.009011
GO:0055076	transition metal ion homeostasis	[8]	8.36E-04	0.01287
GO:0055085	transmembrane transport	[3, 4, 5]	0.018468	0.067182
GO:0055086	nucleobase-containing small molecule metabolic process	[3, 4, 5]	0.027476	0.083513
GO:0055114	oxidation-reduction process	[3]	0.015304	0.059415
GO:0061097	regulation of protein tyrosine kinase activity	[7, 8, 9, 10, 11]	0.016518	0.062043
GO:0070098	chemokine-mediated signaling pathway	[6, 7]	3.84E-04	0.009342
GO:0070232	regulation of T cell apoptotic process	[8, 9, 10]	0.004451	0.029373
GO:0070301	cellular response to hydrogen peroxide	[5, 6]	0.002892	0.023037
GO:0070372	regulation of ERK1 and ERK2 cascade	[6, 7, 8, 9, 10, 11]	6.61E-05	0.003817
GO:0070887	cellular response to chemical stimulus	[3]	0.001906	0.017974
GO:0071216	cellular response to biotic stimulus	[3]	4.79E-05	0.003689
GO:0071310	cellular response to organic substance	[4]	0.023844	0.078127
GO:0071346	cellular response to interferon-gamma	[5, 6]	0.0145	0.059281
GO:0071347	cellular response to interleukin-1	[6]	0.005024	0.031796
GO:0071356	cellular response to tumor necrosis factor	[6]	0.036535	0.097008
GO:0071396	cellular response to lipid	[5]	0.020389	0.071362
GO:0071560	cellular response to transforming growth factor beta stimulu	[4, 6]	0.030513	0.088107
GO:0071702	organic substance transport	[4]	0.017614	0.065625
GO:0080134	regulation of response to stress	[3, 4]	0.020413	0.070909
GO:0097028	dendritic cell differentiation	[6, 7, 8, 9]	0.00384	0.027723
GO:0097190	apoptotic signaling pathway	[4, 5, 6]	0.016143	0.06113
GO:0097191	extrinsic apoptotic signaling pathway	[5, 6, 7]	0.002517	0.021538
GO:0097529	myeloid leukocyte migration	[3, 5, 6, 7]	5.72E-04	0.011496
GO:0098542	defense response to other organism	[3, 4, 5]	0.003838	0.028143
GO:0098581	detection of external biotic stimulus	[4]	8.72E-04	0.012211
GO:0098609	cell-cell adhesion	[3]	0.027978	0.083933
GO:1901136	carbohydrate derivative catabolic process	[4]	0.02409	0.078376
GO:1901565	organonitrogen compound catabolic process	[4]	0.012114	0.052799
GO:1901615	organic hydroxy compound metabolic process	[3]	0.037101	0.097946
GO:1901700	response to oxygen-containing compound	[3]	0.026724	0.083421
GO:1901701	cellular response to oxygen-containing compound	[4]	0.009064	0.042731
GO:1902105	regulation of leukocyte differentiation	[4, 5, 6, 7, 8, 9]	0.028788	0.085256

S4 Tables

Table 1. Gene set enrichment analysis results based on biological process

GO:1902106	negative regulation of leukocyte differentiation	[4, 5, 6, 7, 8, 9, 10]	0.03087	0.088036
GO:1902532	negative regulation of intracellular signal transduction	[4, 5, 6, 7]	0.018476	0.066687
GO:1902533	positive regulation of intracellular signal transduction	[4, 5, 6, 7]	0.021672	0.073084
GO:1903034	regulation of response to wounding	[4, 5]	0.008698	0.041861
GO:1903036	positive regulation of response to wounding	[3, 4, 5, 6]	0.031786	0.089543
GO:1903706	regulation of hemopoiesis	[3, 4, 5, 6, 7, 8]	0.004632	0.030138
GO:1903708	positive regulation of hemopoiesis	[3, 4, 5, 6, 7, 8, 9]	0.006814	0.037036
GO:2001233	regulation of apoptotic signaling pathway	[4, 5, 6, 7]	0.005579	0.033047
GO:2001234	negative regulation of apoptotic signaling pathway	[4, 5, 6, 7, 8]	0.001237	0.015034
GO:2001235	positive regulation of apoptotic signaling pathway	[4, 5, 6, 7, 8]	0.024311	0.078542
GO:2001237	negative regulation of extrinsic apoptotic signaling pathway	[5, 6, 7, 8, 9]	0.002131	0.019301

S4 Tables

Table 2. Gene set enrichment analysis results based on cellular component.

**Cellular Component Enrichment Analysis for X3**				
**GOID**	**GOTerm**	**GOLevels**	***p* - value**	**FDR (BH)**
GO:0005764	lysosome	[6, 7, 8, 9]	2.05E-02	3.33E-02
GO:0005886	plasma membrane	[2, 3, 4]	6.56E-04	4.26E-03
GO:0005925	focal adhesion	[4, 5]	1.44E-02	2.68E-02
GO:0009986	cell surface	[2, 3]	1.07E-03	4.65E-03
GO:0016021	integral component of membrane	[3, 4]	1.73E-03	5.63E-03
GO:0044459	plasma membrane part	[2, 3, 4, 5]	4.94E-02	7.13E-02
GO:0045121	membrane raft	[4, 5]	6.15E-04	7.99E-03
GO:0098552	side of membrane	[2, 3]	4.52E-03	1.18E-02
GO:0098589	membrane region	[2, 3]	4.94E-03	1.07E-02
**Cellular Component Enrichment Analysis for X1,X2,X3**				
**GOID**	**GOTerm**	**GOLevels**	***p* - value**	**FDR (BH)**
GO:0005764	lysosome	[6, 7, 8, 9]	1.29E-02	9.74E-02
GO:0005905	coated pit	[2, 3, 4]	1.59E-02	9.83E-02
GO:0009897	external side of plasma membrane	[3, 4, 5, 6]	1.05E-03	3.57E-02
GO:0009986	cell surface	[2, 3]	6.53E-04	4.44E-02
GO:0031224	intrinsic component of membrane	[2, 3]	1.16E-02	9.83E-02
GO:0036064	ciliary basal body	[3, 4, 5, 6, 7, 8, 9, 10]	1.01E-02	9.85E-02
GO:0044459	plasma membrane part	[2, 3, 4, 5]	1.80E-03	4.08E-02
GO:0060170	ciliary membrane	[3, 4, 5, 6, 7, 8]	1.33E-02	9.05E-02
GO:0071944	cell periphery	[2, 3]	5.57E-03	6.32E-02
GO:0098552	side of membrane	[2, 3]	3.70E-03	5.04E-02
GO:0098589	membrane region	[2, 3]	2.00E-03	3.39E-02

S4 Tables

Table 3. Gene set enrichment analysis results based on pathway

**Pathway Enrichment Analysis for X3**			
**GOID**	**GOTerm**	***p* - value**	**FDR(BH)**
KEGG:04060	Cytokine-cytokine receptor interaction	1.81E-02	2.02E-02
KEGG:04145	Phagosome	5.48E-04	1.74E-03
KEGG:04514	Cell adhesion molecules (CAMs)	4.70E-03	8.11E-03
KEGG:04620	Toll-like receptor signaling pathway	1.19E-03	2.83E-03
KEGG:05140	Leishmaniasis	3.26E-04	1.24E-03
KEGG:05152	Tuberculosis	5.83E-03	8.52E-03
KEGG:05323	Rheumatoid arthritis	3.09E-05	5.88E-04
**Pathway Enrichment Analysis for X1,X2,X3**			
**GOID**	**GOTerm**	***p* - value**	**FDR(BH)**
KEGG:04060	Cytokine-cytokine receptor interaction	4.66E-03	6.81E-02
KEGG:04380	Osteoclast differentiation	3.35E-03	7.83E-02
KEGG:04672	Intestinal immune network for IgA production	9.17E-03	8.94E-02
KEGG:04726	Serotonergic synapse	4.38E-03	7.32E-02
KEGG:04978	Mineral absorption	1.18E-02	9.88E-02
KEGG:05140	Leishmaniasis	5.47E-03	7.12E-02
KEGG:05145	Toxoplasmosis	9.08E-03	9.66E-02
KEGG:05220	Chronic myeloid leukemia	8.28E-03	9.68E-02
KEGG:05323	Rheumatoid arthritis	2.42E-03	7.08E-02
