# Supplementary material for: Three-way interaction model to trace the mechanisms involved in Alzheimer’s disease transgenic mice
Source: PLoS One. 2017 Sep 21;12(9):e0184697. doi: 10.1371/journal.pone.0184697 (PMC5608283; doi:10.1371/journal.pone.0184697)
Supplement: S5 Table — (PDF) [file pone.0184697.s005.pdf]

S5 Table: The categorized biological processes based on the AD-related pathways

Alzheimer's disease-related pathways

GO ID	GO Term	GO Level
	**Inflammation and Immune System Activation**	
GO:0002763	Positive regulation of myeloid leukocyte differentiation	11
GO:0001779	Natural killer cell differentiation	10
GO:0002221	Pattern recognition receptor signaling pathway	10
GO:0002286	T cell activation involved in immune response	10
GO:0030593	Neutrophil chemotaxis	10
GO:0042098	T cell proliferation	10
GO:0048245	Eosinophil chemotaxis	10
GO:1902106	Negative regulation of leukocyte differentiation	10
GO:0002573	Myeloid leukocyte differentiation	9
GO:0002690	Positive regulation of leukocyte chemotaxis	9
GO:0033559	Unsaturated fatty acid metabolic process	9
GO:0042110	T cell activation	9
GO:0045637	Regulation of myeloid cell differentiation	9
GO:0050851	Antigen receptor-mediated signaling pathway	9
GO:0097028	Dendritic cell differentiation	9
GO:1902105	Regulation of leukocyte differentiation	9
GO:0002521	Leukocyte differentiation	8
GO:0002548	Monocyte chemotaxis	8
GO:0030099	Myeloid cell differentiation	8
GO:0048247	Lymphocyte chemotaxis	8
GO:0050663	Cytokine secretion	8
GO:0002312	B cell activation involved in immune response	7
GO:0002643	Regulation of tolerance induction	7
GO:0002757	Immune response-activating signal transduction	7
GO:0030595	Leukocyte chemotaxis	7
GO:0070098	Chemokine-mediated signaling pathway	7
GO:0097529	Myeloid leukocyte migration	7
GO:0002285	Lymphocyte activation involved in immune response	6
GO:0006909	Phagocytosis	6
GO:0019221	Cytokine-mediated signaling pathway	6
GO:0031349	Positive regulation of defense response	6
GO:0032689	Negative regulation of interferon-gamma production	6
GO:0032729	Positive regulation of interferon-gamma production	6
GO:0032735	Positive regulation of interleukin-12 production	6
GO:0045576	Mast cell activation	6
GO:0046651	Lymphocyte proliferation	6
GO:0048469	Cell maturation	6
GO:0050727	Regulation of inflammatory response	6
GO:0050900	Leukocyte migration	6

S5 Table: The categorized biological processes based on the AD-related pathways

GO:0071346	Cellular response to interferon-gamma	6
GO:0071347	Cellular response to interleukin-1	6
GO:0071356	Cellular response to tumor necrosis factor	6
GO:0071560	Cellular response to transforming growth factor beta stimulus	6
GO:0001818	Negative regulation of cytokine production	5
GO:0001819	Positive regulation of cytokine production	5
GO:0002274	Myeloid leukocyte activation	5
GO:0002366	Leukocyte activation involved in immune response	5
GO:0002520	Immune system development	5
GO:0008285	Negative regulation of cell proliferation	5
GO:0031347	Regulation of defense response	5
GO:0032660	Regulation of interleukin-17 production	5
GO:0034341	Response to interferon-gamma	5
GO:0046649	Lymphocyte activation	5
GO:0001817	Regulation of cytokine production	4
GO:0002367	Cytokine production involved in immune response	4
GO:0002444	Myeloid leukocyte mediated immunity	4
GO:0002577	Regulation of antigen processing and presentation	4
GO:0002683	Negative regulation of immune system process	4
GO:0002684	Positive regulation of immune system process	4
GO:0002697	Regulation of immune effector process	4
GO:0006954	Inflammatory response	4
GO:0032101	Regulation of response to external stimulus	4
GO:0032609	Interferon-gamma production	4
GO:0032623	Interleukin-2 production	4
GO:0034097	Response to cytokine	4
GO:0045087	Innate immune response	4
GO:0045321	Leukocyte activation	4
GO:0050776	Regulation of immune response	4
GO:0080134	Regulation of response to stress	4
GO:0001816	Cytokine production	3
GO:0002682	Regulation of immune system process	3
GO:0006952	Defense response	3
**Apoptosis**		
GO:0043409	Negative regulation of MAPK cascade	11
GO:0043410	Positive regulation of MAPK cascade	11
GO:0070372	Regulation of ERK1 and ERK2 cascade	11
GO:0070232	Regulation of T cell apoptotic process	10
GO:0031663	Lipopolysaccharide-mediated signaling pathway	9
GO:2001237	Negative regulation of extrinsic apoptotic signaling pathway	9
GO:2001234	Negative regulation of apoptotic signaling pathway	8
GO:2001235	Positive regulation of apoptotic signaling pathway	8
GO:0043065	Positive regulation of apoptotic process	7

S5 Table: The categorized biological processes based on the AD-related pathways

GO:0043066	Negative regulation of apoptotic process	7
GO:0097191	Extrinsic apoptotic signaling pathway	7
GO:2001233	Regulation of apoptotic signaling pathway	7
GO:0008637	Apoptotic mitochondrial changes	6
GO:0042981	Regulation of apoptotic process	6
GO:0043491	Protein kinase B signaling	6
GO:0097190	Apoptotic signaling pathway	6
GO:0031325	Positive regulation of cellular metabolic process	5
GO:0071396	Cellular response to lipid	5
GO:0012501	Programmed cell death	4
GO:0071310	Cellular response to organic substance	4
GO:1901701	Cellular response to oxygen-containing compound	4
GO:0007586	Digestion	3
GO:1901700	Response to oxygen-containing compound	3
**Oxidative Stress**		
GO:0061097	Regulation of protein tyrosine kinase activity	11
GO:0050730	Regulation of peptidyl-tyrosine phosphorylation	10
GO:0046496	Nicotinamide nucleotide metabolic process	9
GO:0009166	Nucleotide catabolic process	8
GO:0023014	Signal transduction by protein phosphorylation	8
GO:0009119	Ribonucleoside metabolic process	7
GO:0006690	Icosanoid metabolic process	7
GO:0031331	Positive regulation of cellular catabolic process	6
GO:0033238	Regulation of cellular amine metabolic process	6
GO:0070301	Cellular response to hydrogen peroxide	6
GO:0010604	Positive regulation of macromolecule metabolic process	5
GO:0043085	Positive regulation of catalytic activity	5
GO:0043467	Regulation of generation of precursor metabolites and energy	5
GO:0046434	Organophosphate catabolic process	5
GO:0055086	Nucleobase-containing small molecule metabolic process	5
GO:0009893	Positive regulation of metabolic process	4
GO:0016052	Carbohydrate catabolic process	4
GO:1901136	Carbohydrate derivative catabolic process	4
GO:1901565	Organonitrogen compound catabolic process	4
GO:0006066	Alcohol metabolic process	4
GO:0055114	Oxidation-reduction process	3
GO:1901615	Organic hydroxy compound metabolic process	3
**Microglia Activation and Migration**		
GO:0008347	Glial cell migration	8
GO:0032496	Response to lipopolysaccharide	7
GO:0042063	Gliogenesis	7
GO:0002833	Positive regulation of response to biotic stimulus	5
GO:0032103	Positive regulation of response to external stimulus	5

S5 Table: The categorized biological processes based on the AD-related pathways

GO:0051272	Positive regulation of cellular component movement	5
GO:0002831	Regulation of response to biotic stimulus	4
GO:0043902	Positive regulation of multi-organism process	4
GO:0048584	Positive regulation of response to stimulus	4
GO:0050865	Regulation of cell activation	4
GO:0051241	Negative regulation of multicellular organismal process	4
GO:0048522	Positive regulation of cellular process	4
GO:0098581	Detection of external biotic stimulus	4
GO:0001775	Cell activation	3
GO:0043900	Regulation of multi-organism process	3
GO:0048583	Regulation of response to stimulus	3
GO:0071216	Cellular response to biotic stimulus	3
GO:0098609	Cell-cell adhesion	3
**Calcium Signaling Pathway**		
GO:0050848	Regulation of calcium-mediated signaling	8
GO:1902532	Negative regulation of intracellular signal transduction	7
GO:1902533	Positive regulation of intracellular signal transduction	7
GO:0019932	Second-messenger-mediated signaling	6
GO:0007166	Cell surface receptor signaling pathway	5
GO:0009966	Regulation of signal transduction	5
GO:0010647	Positive regulation of cell communication	5
GO:0055085	Transmembrane transport	5
GO:0023056	Positive regulation of signaling	4
GO:0006810	Transport	3
GO:0048518	Positive regulation of biological process	3
**Apolipoprotein E and Phospholipid Transport Dysfunction [1]**		
GO:0015696	Ammonium transport	8
GO:0015914	Phospholipid transport	8
GO:0006820	Anion transport	6
GO:0008203	Cholesterol metabolic process	6
GO:0006811	Ion transport	5
GO:0015844	Monoamine transport	5
GO:0044765	Single-organism transport	4
GO:0071702	Organic substance transport	4
GO:0010876	Lipid localization	3
**Neurofibrillary Tangles Formation**		
GO:0031401	Positive regulation of protein modification process	8
GO:0051017	Actin filament bundle assembly	8
GO:0006468	Protein phosphorylation	7
GO:0042325	Regulation of phosphorylation	7
GO:0045937	Positive regulation of phosphate metabolic process	7
GO:0019220	Regulation of phosphate metabolic process	6

S5 Table: The categorized biological processes based on the AD-related pathways

GO:0031532	Actin cytoskeleton reorganization	6
GO:0071560	Cellular response to transforming growth factor beta stimulus	6
GO:0016310	Phosphorylation	5
**DNA Damage**		
GO:0045944	Positive regulation of transcription from RNA polymerase II promoter	12
GO:1903036	Positive regulation of response to wounding	6
GO:0002833	Positive regulation of response to biotic stimulus	5
GO:1903034	Regulation of response to wounding	5
GO:0002831	Regulation of response to biotic stimulus	4
GO:0009611	Response to wounding	3
GO:0070887	Cellular response to chemical stimulus	3
GO:0071216	Cellular response to biotic stimulus	3
**Hematoposis [2,3]**		
GO:1903708	Positive regulation of hemopoiesis	9
GO:0055076	Transition metal ion homeostasis	8
GO:1903706	Regulation of hemopoiesis	8
GO:0030193	Regulation of blood coagulation	7
**Protein Oxidation**		
GO:0032270	Positive regulation of cellular protein metabolic process	7
GO:0051247	Positive regulation of protein metabolic process	6
GO:0051246	Regulation of protein metabolic process	5

pathways with unclear Alzheimer's disease relation

GO ID	GO Term	GO Level
GO:0035987	Endodermal cell differentiation	9
GO:0001942	Hair follicle development	8
GO:0050691	Regulation of defense response to virus by host	8
GO:0050829	Defense response to Gram-negative bacterium	7
GO:0042742	Defense response to bacterium	6
GO:0009617	Response to bacterium	5
GO:0009620	Response to fungus	5
GO:0046847	Filopodium assembly	5
GO:0098542	Defense response to other organism	5
GO:0051707	Response to other organism	4

S5 Table: The categorized biological processes based on the AD-related pathways

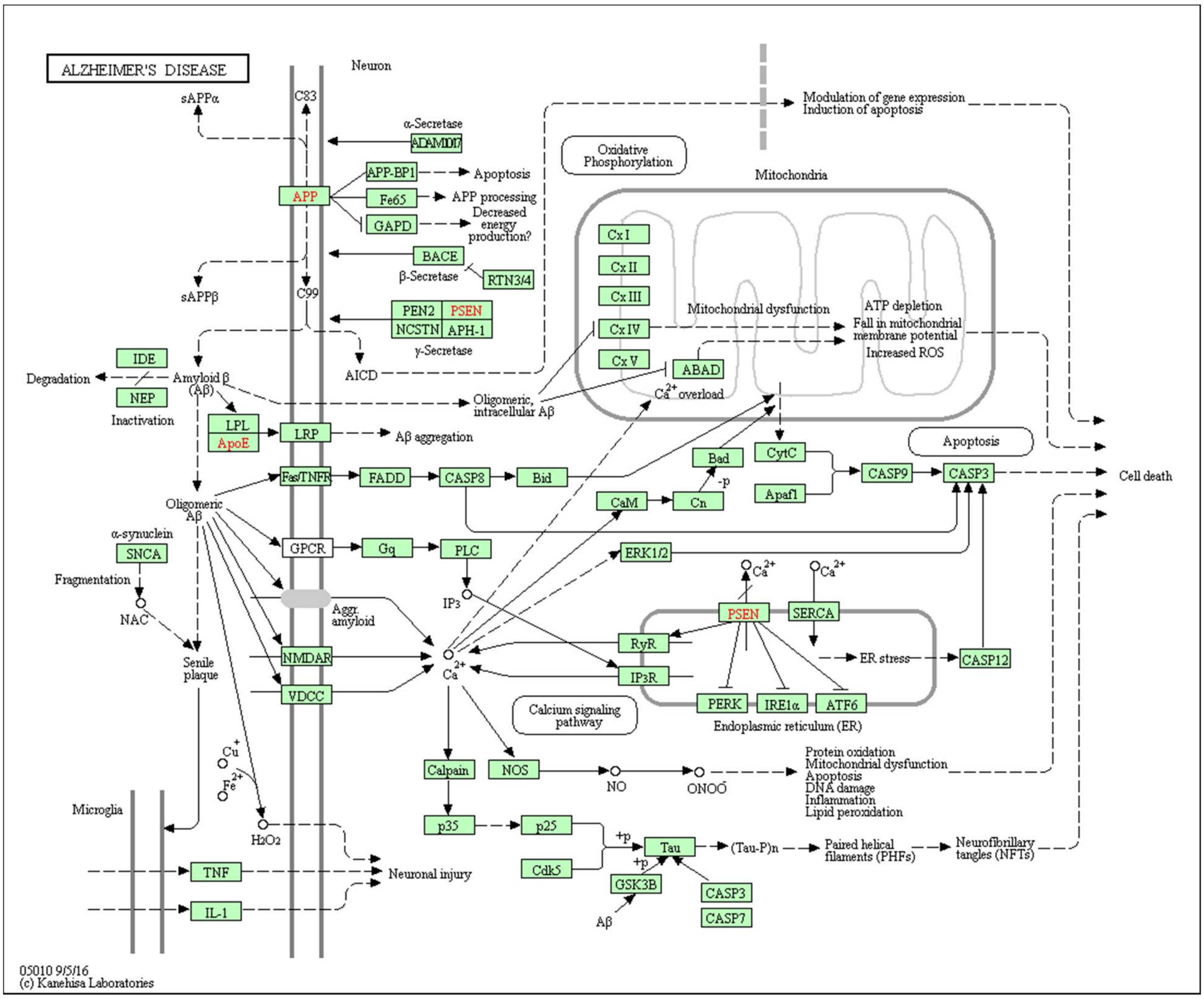

Based on the KEGG database [4], oxidative stress, calcium signaling pathway, apoptosis, inflammation, microglia activation and migration, apolipoprotein E and phospholipid transport dysfunction, neurofibrillary tangle formation, DNA damage and protein oxidation are well-known Alzheimer’s disease related pathways.

References:

1. Liu C-C, Kanekiyo T, Xu H, Bu G (2013) Apolipoprotein E and Alzheimer disease: risk, mechanisms and therapy. Nature Reviews Neurology 9: 106-118.
2. Maler JM, Spitzer P, Lewczuk P, Kornhuber J, Herrmann M, et al. (2006) Decreased circulating CD34+ stem cells in early Alzheimer's disease: Evidence for a deficient hematopoietic brain support? Mol Psychiatry 11: 1113-1115.

S5 Table: The categorized biological processes based on the AD-related pathways

3. Ray S, Britschgi M, Herbert C, Takeda-Uchimura Y, Boxer A, et al. (2007) Classification and prediction of clinical Alzheimer's diagnosis based on plasma signaling proteins. Nature medicine 13: 1359-1362.
4. Kanehisa M, Furumichi M, Tanabe M, Sato Y, Morishima K (2017) KEGG: new perspectives on genomes, pathways, diseases and drugs. Nucleic Acids Res 45: D353-D361.
