# Supplementary material for: Three-way interaction model to trace the mechanisms involved in Alzheimer’s disease transgenic mice
Source: PLoS One. 2017 Sep 21;12(9):e0184697. doi: 10.1371/journal.pone.0184697 (PMC5608283; doi:10.1371/journal.pone.0184697)

S1 Figs. This file includes array images, MA plots, box plots and data histograms before and after of normalization

Array images. Two arrays (15.w.3 and 26.w.12) were removed from dataset, because the images of these arrays haven't an appropriate quality.

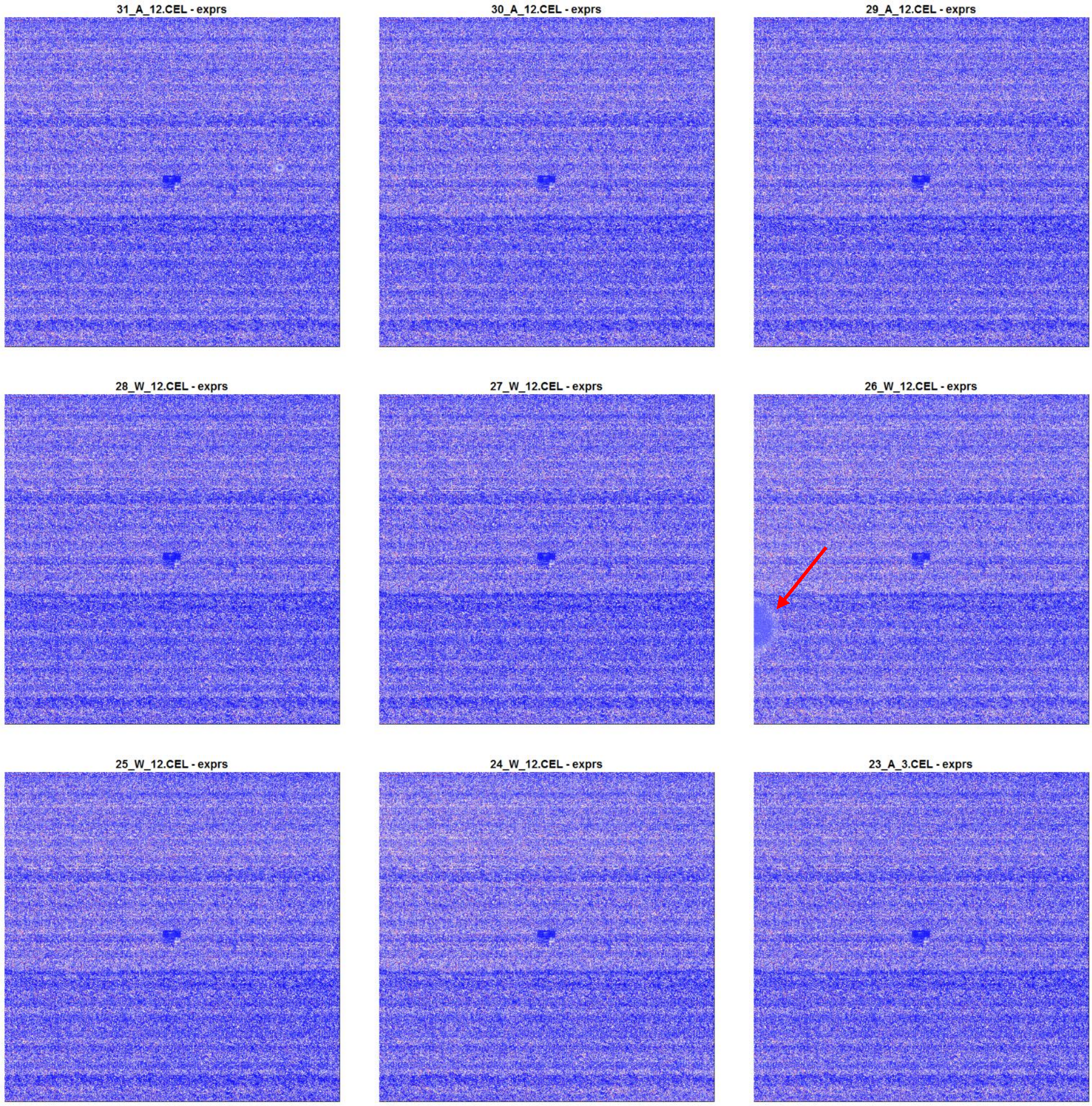

S1 Figs. This file includes array images, MA plots, box plots and data histograms before and after of normalization

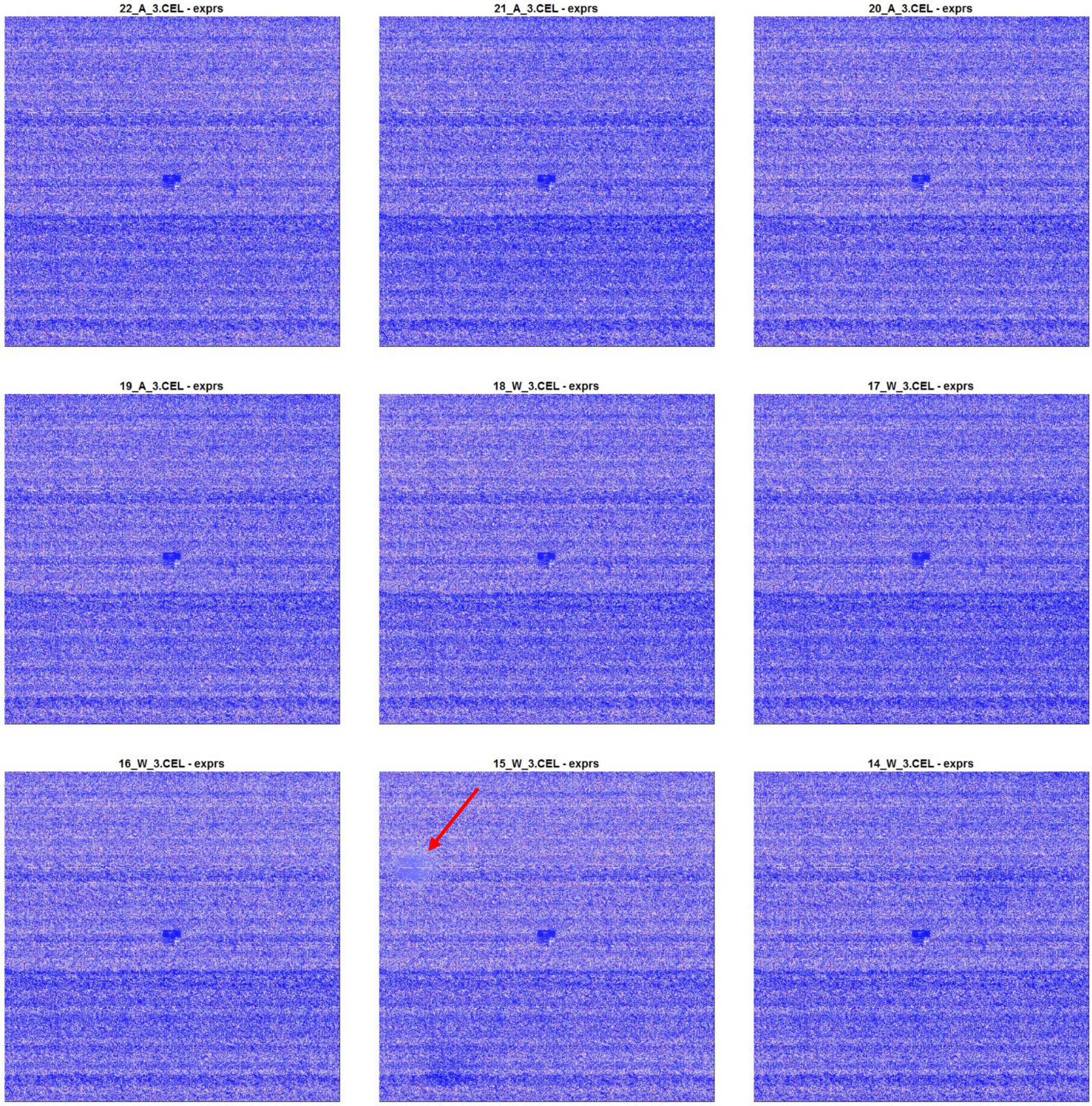

S1 Figs. This file includes array images, MA plots, box plots and data histograms before and after of normalization

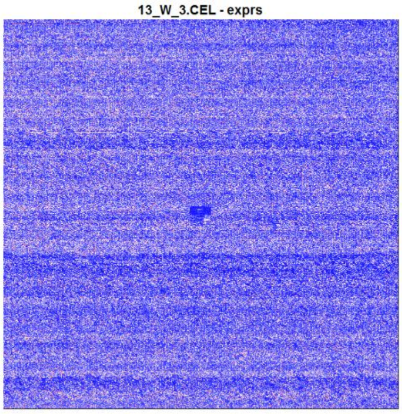

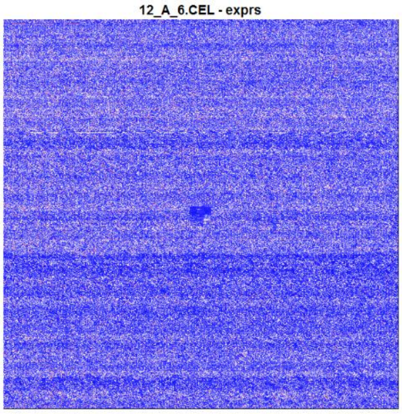

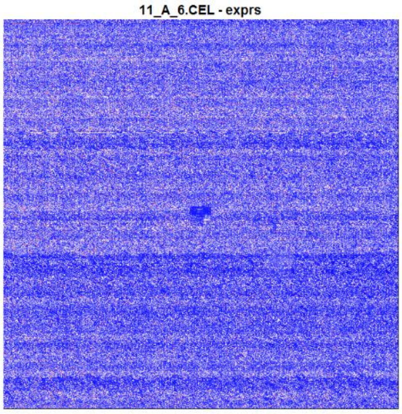

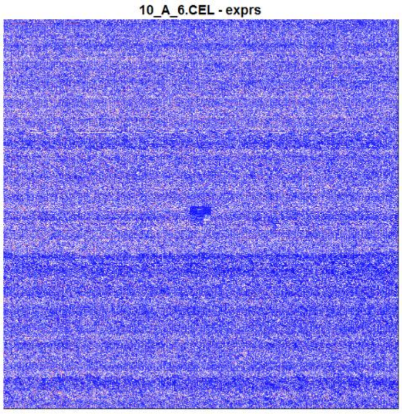

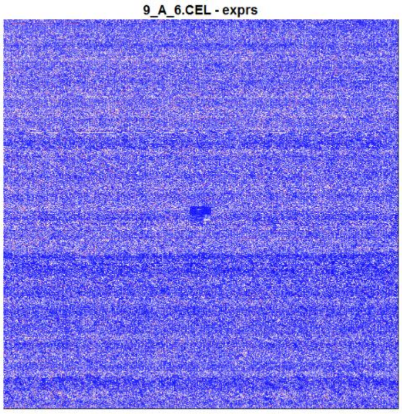

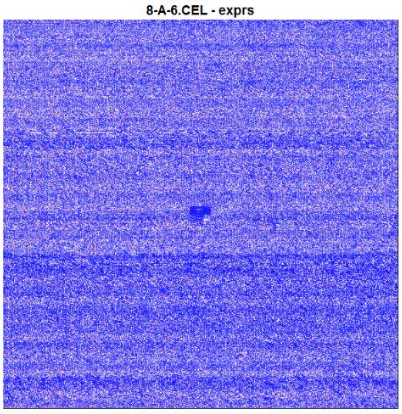

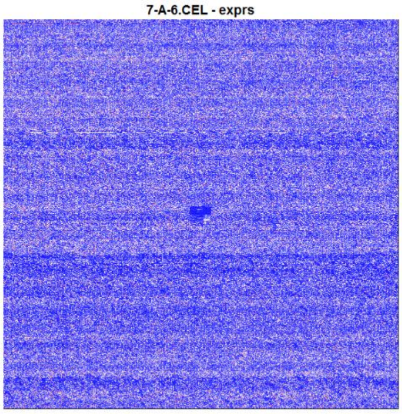

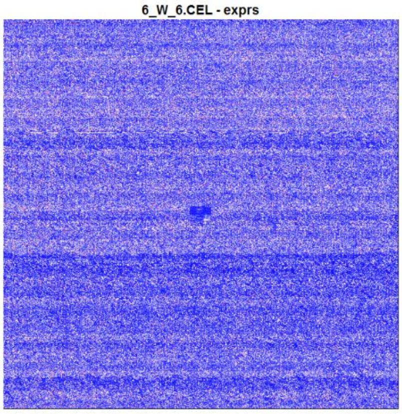

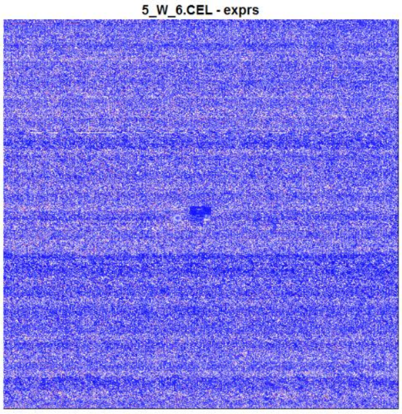

S1 Figs. This file includes array images, MA plots, box plots and data histograms before and after of normalization

4_W_6.CEL - exprs

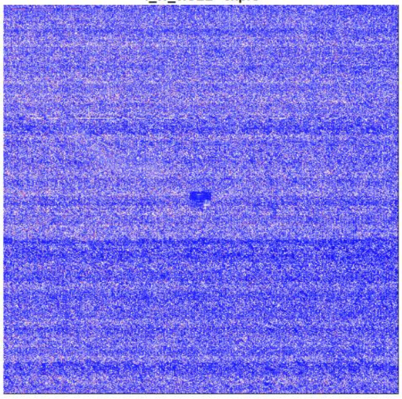

3_W_6.CEL - exprs

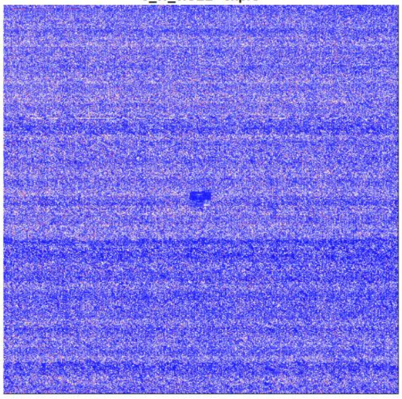

2_W_6.CEL - exprs

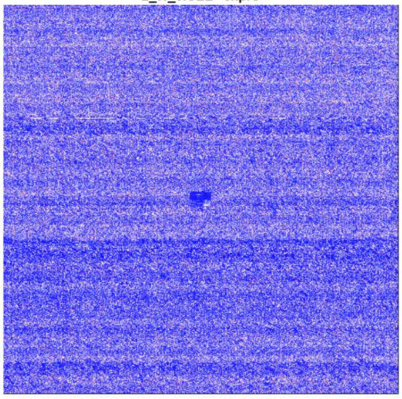

1_W_6.CEL - exprs

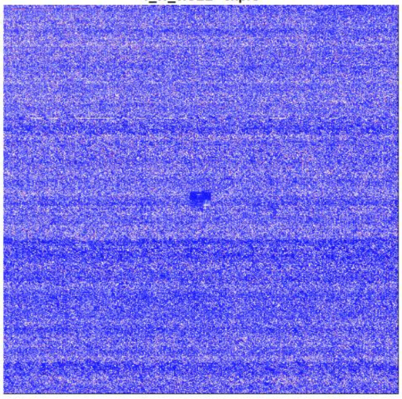

32_A_12.CEL - exprs

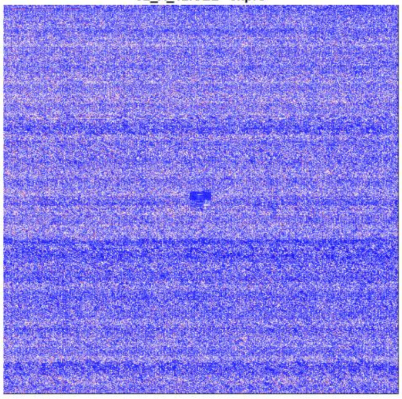

S1 Figs. This file includes array images, MA plots, box plots and data histograms before and after of normalization

MA plots. MA plot of each array versus pseudo median reference chip.

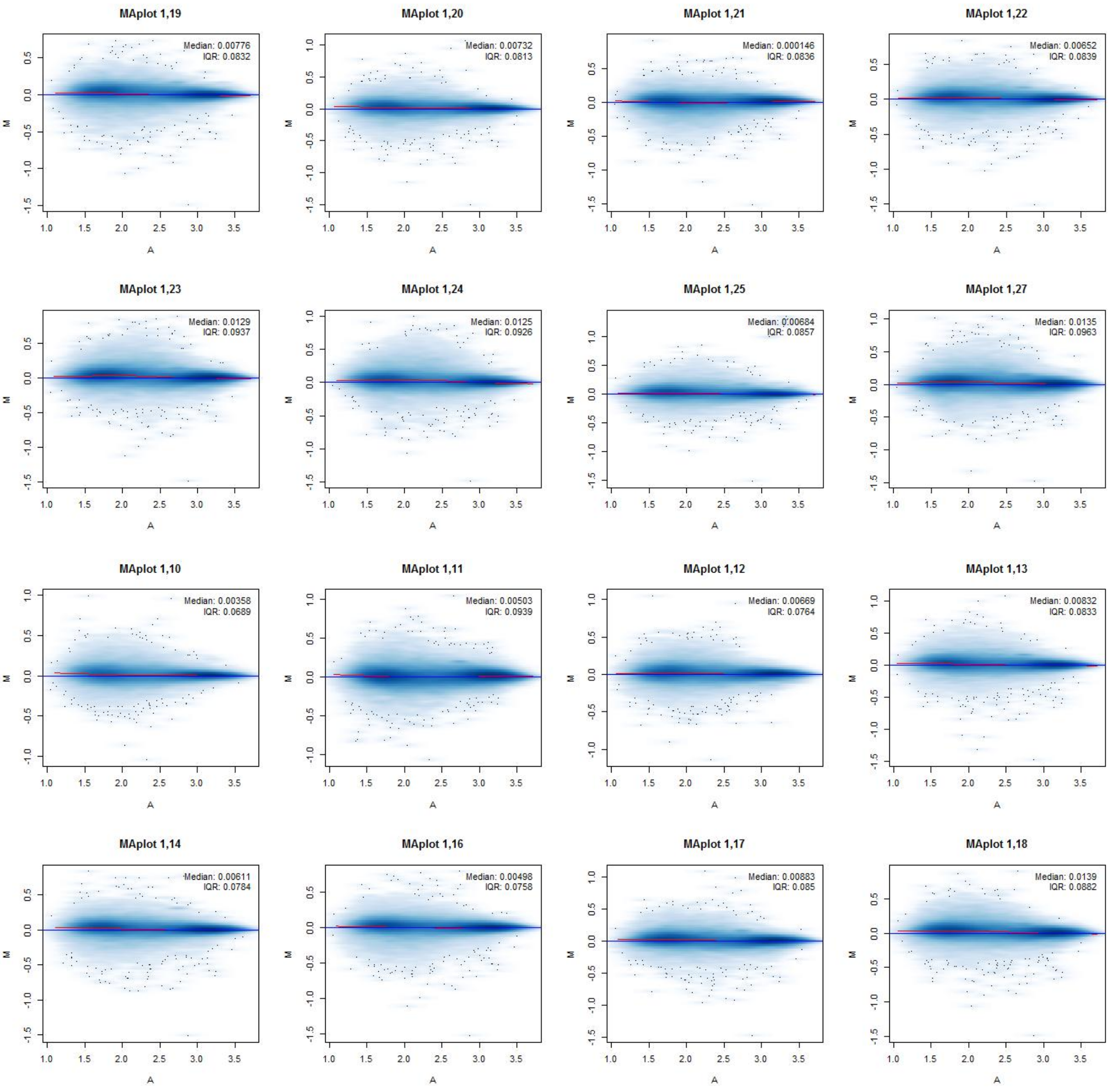

S1 Figs. This file includes array images, MA plots, box plots and data histograms before and after of normalization

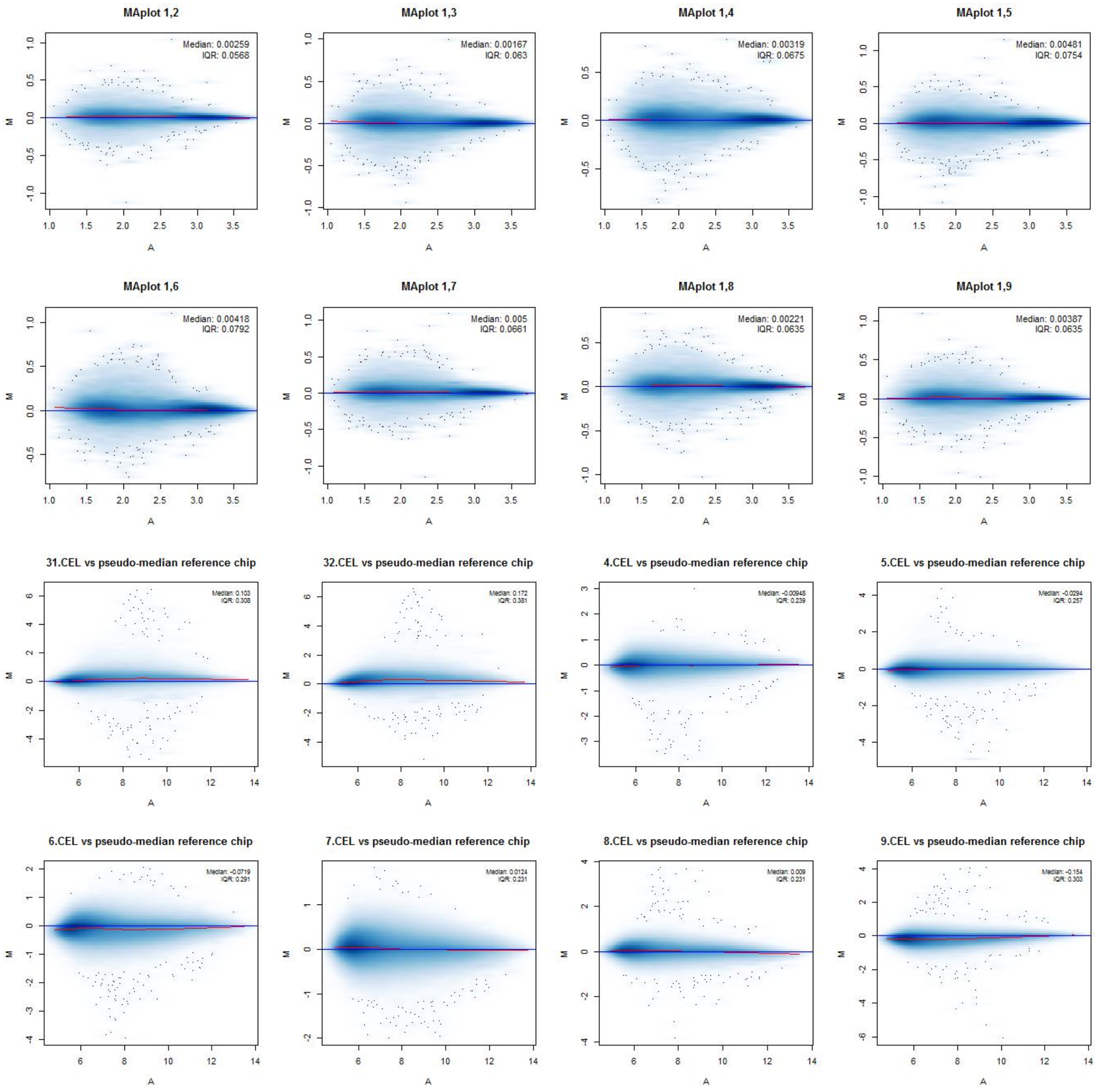

S1 Figs. This file includes array images, MA plots, box plots and data histograms before and after of normalization

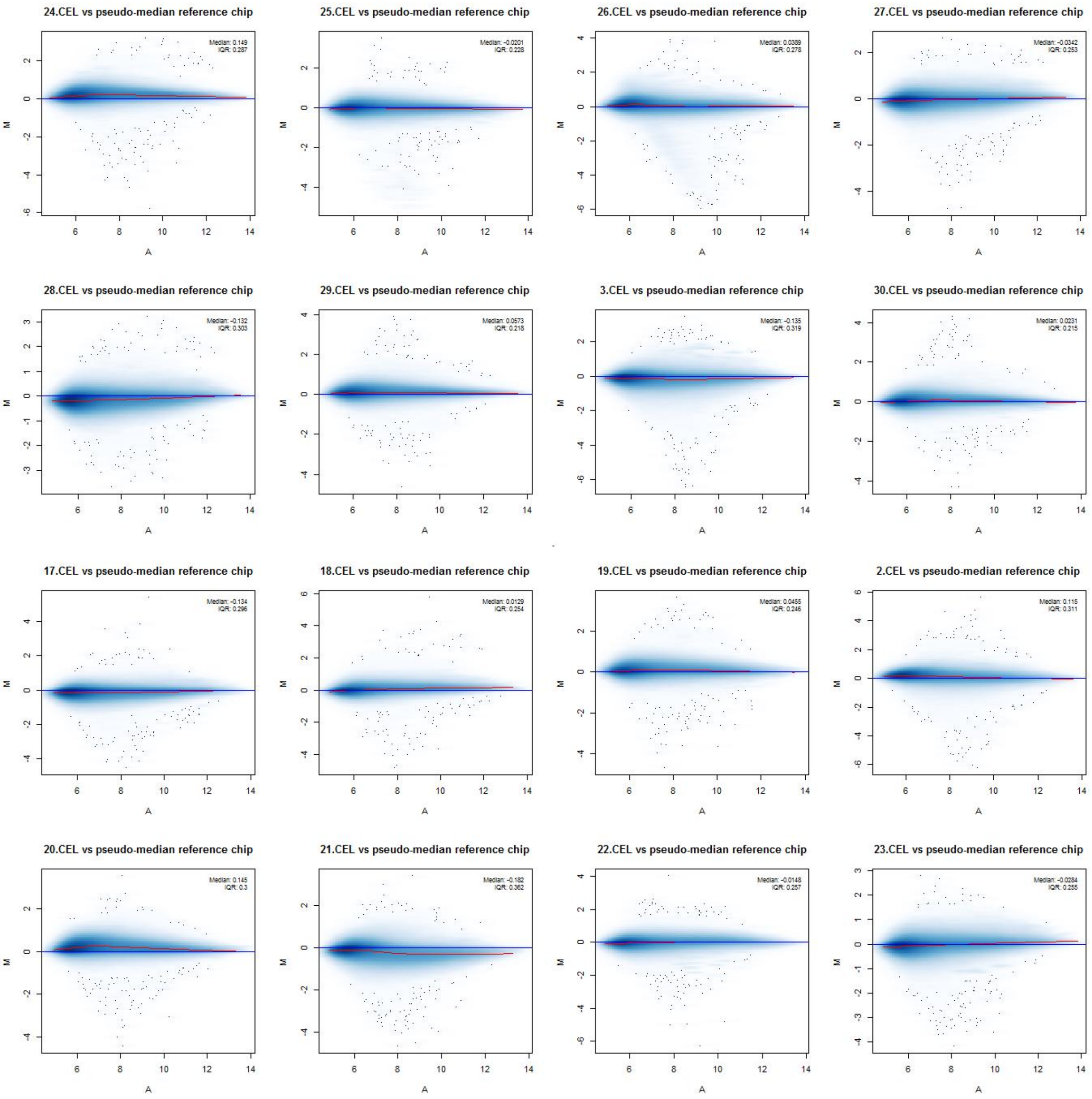

S1 Figs. This file includes array images, MA plots, box plots and data histograms before and after of normalization

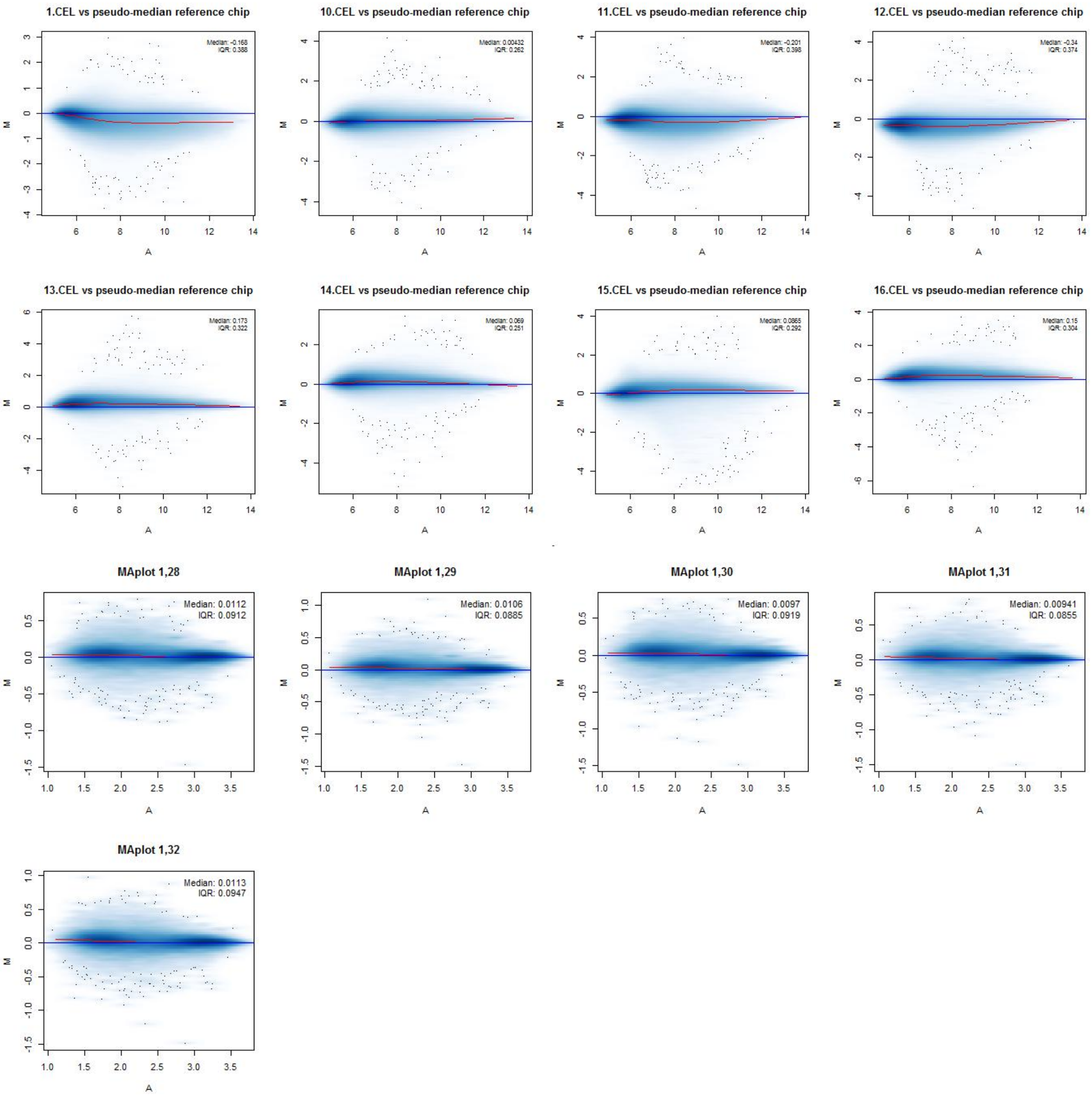

S1 Figs. This file includes array images, MA plots, box plots and data histograms before and after of normalization

Box plots. Box plot before normalization (A); Box plot after normalization (B).

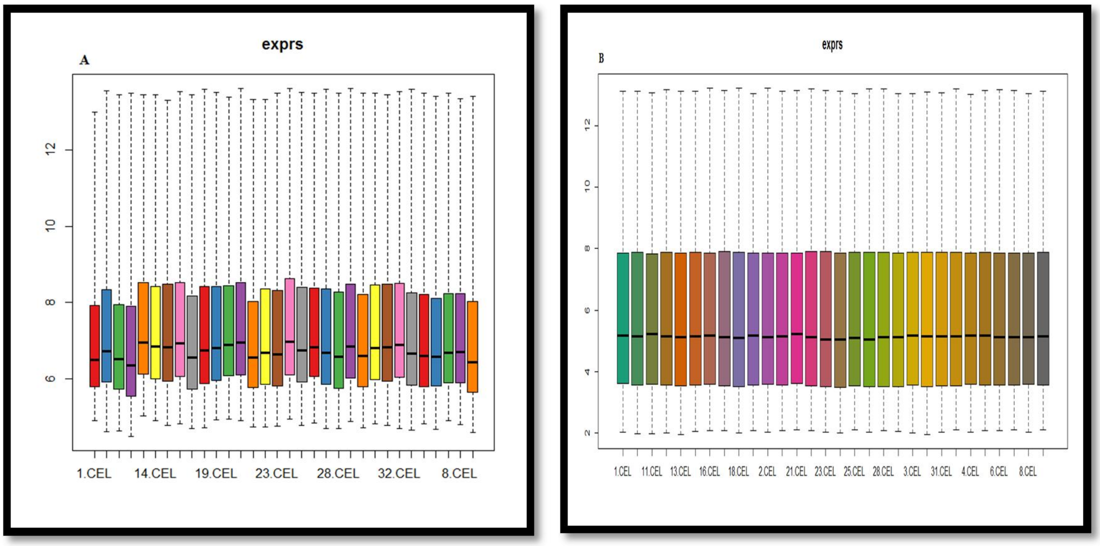

Histograms. Histogram before normalization (A); Histogram after normalization (B).

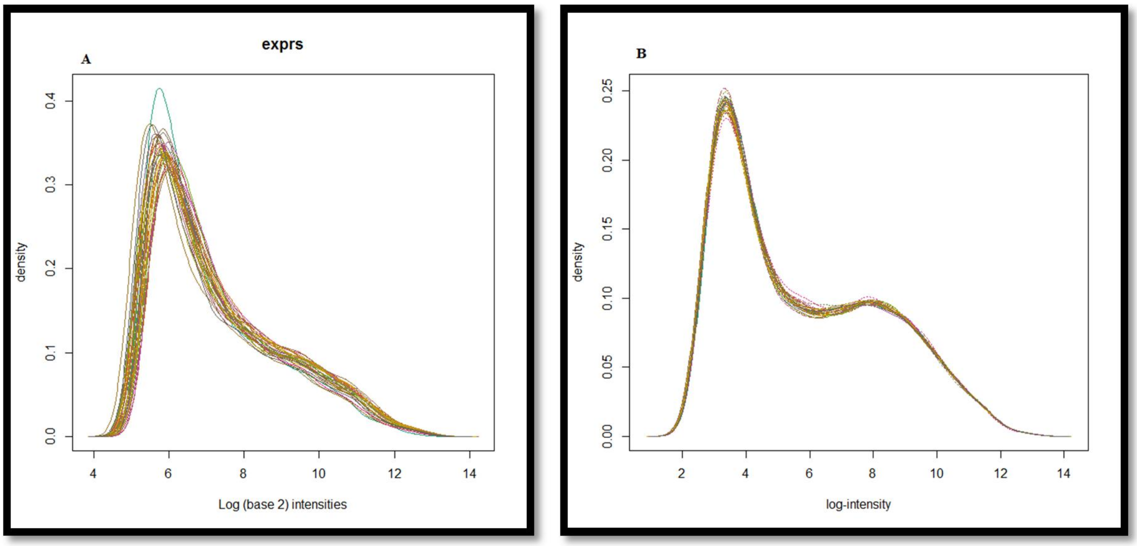

Supplement: S1 Fig — (PDF) [file pone.0184697.s006.pdf]
