# Supplementary figures and images for: Three-way interaction model to trace the mechanisms involved in Alzheimer’s disease transgenic mice
Source: PLoS One. 2017 Sep 21;12(9):e0184697. doi: 10.1371/journal.pone.0184697 (PMC5608283; doi:10.1371/journal.pone.0184697)

**S 2 Fig.** The  $p$ -value histogram of the top 300000 three-way interactions.

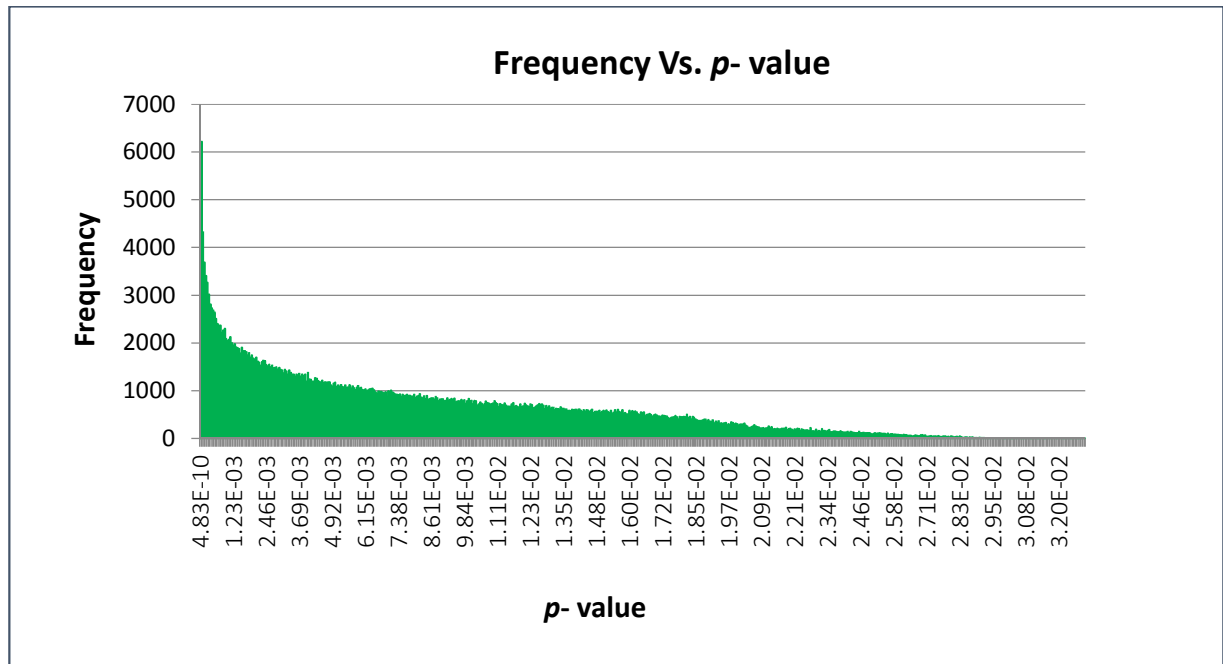

Supplement: S2 Fig — (PDF) [file pone.0184697.s007.pdf]
