# Supplementary material for: Three-way interaction model to trace the mechanisms involved in Alzheimer’s disease transgenic mice
Source: PLoS One. 2017 Sep 21;12(9):e0184697. doi: 10.1371/journal.pone.0184697 (PMC5608283; doi:10.1371/journal.pone.0184697)

S3 Fig. Statistically significant triplets Network. This network shows that Slc14a1, Dock8, Slc11a1 and Parvg with 10, 7, 7 and 6 connections respectively have most contribution in the statistically significant triplets rather than other $X_{3.}$

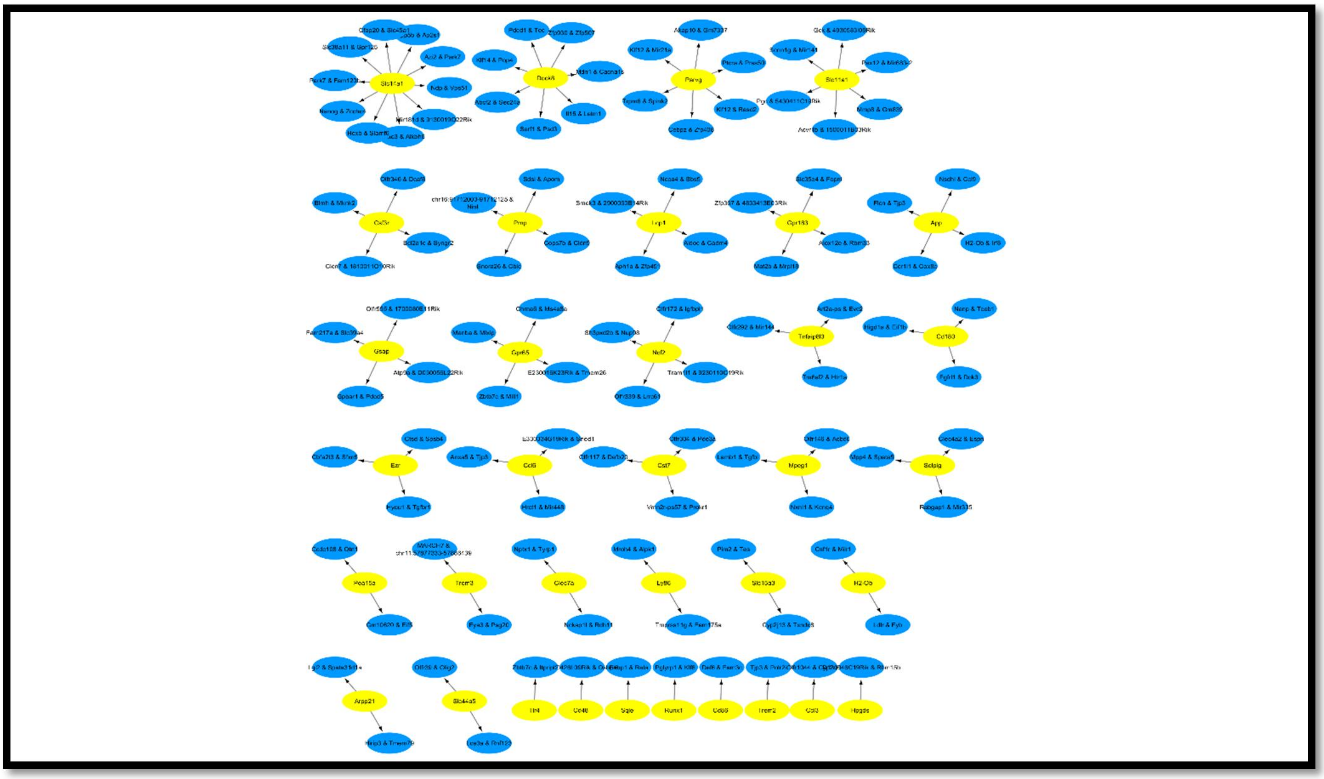

Supplement: S3 Fig — (PDF) [file pone.0184697.s008.pdf]
