# Supplementary material for: Three-way interaction model to trace the mechanisms involved in Alzheimer’s disease transgenic mice
Source: PLoS One. 2017 Sep 21;12(9):e0184697. doi: 10.1371/journal.pone.0184697 (PMC5608283; doi:10.1371/journal.pone.0184697)

S4 Figs. Scatter plots of 12 triplets in which X_1 and X_2 are involved in the same biological process.

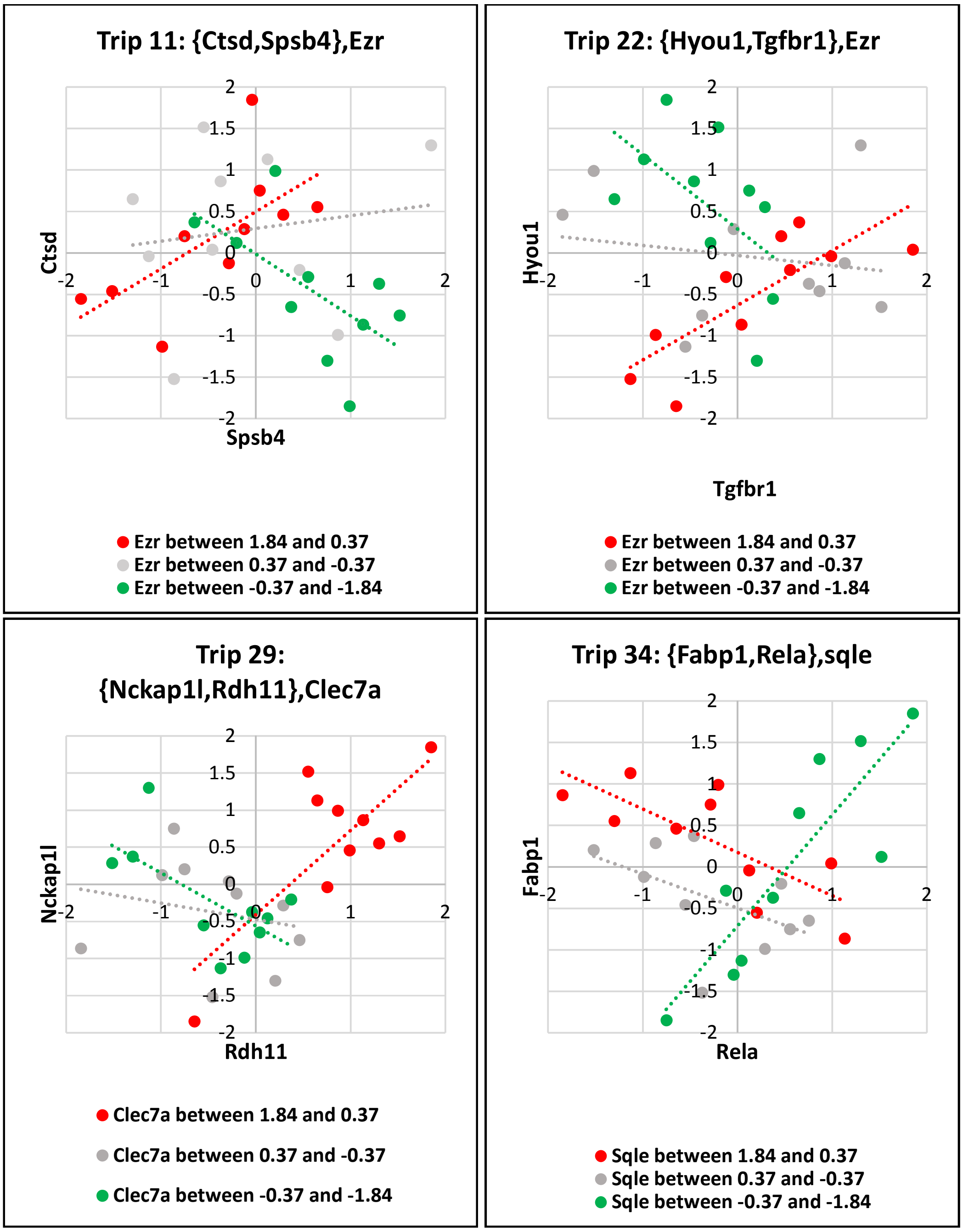

S4 Figs. Scatter plots of 12 triplets in which X_1 and X_2 are involved in the same biological process.

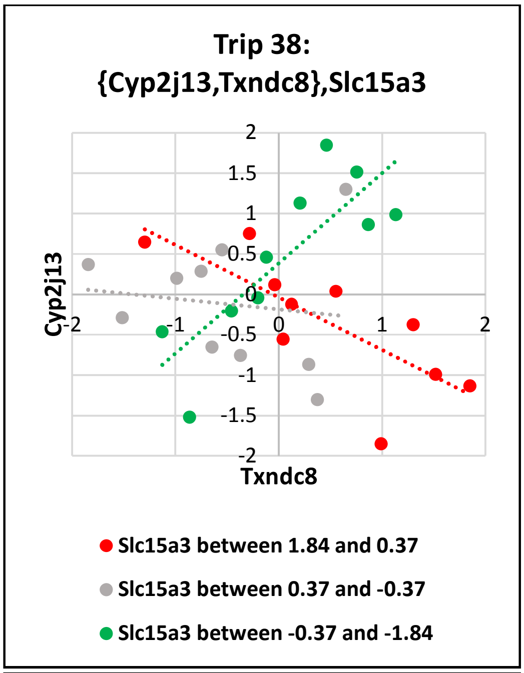

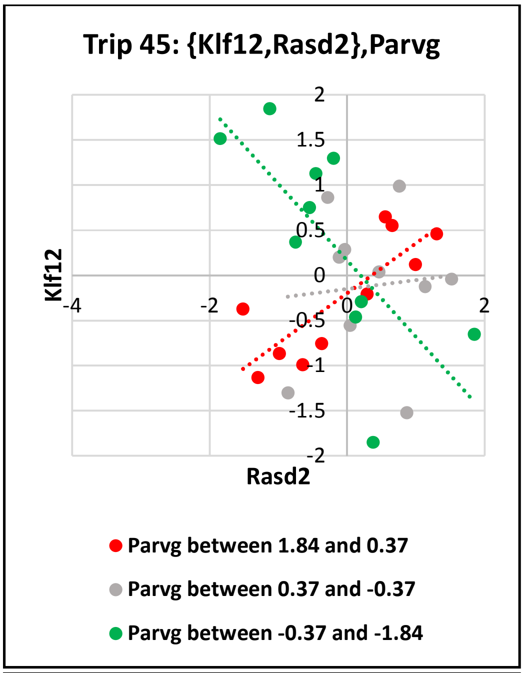

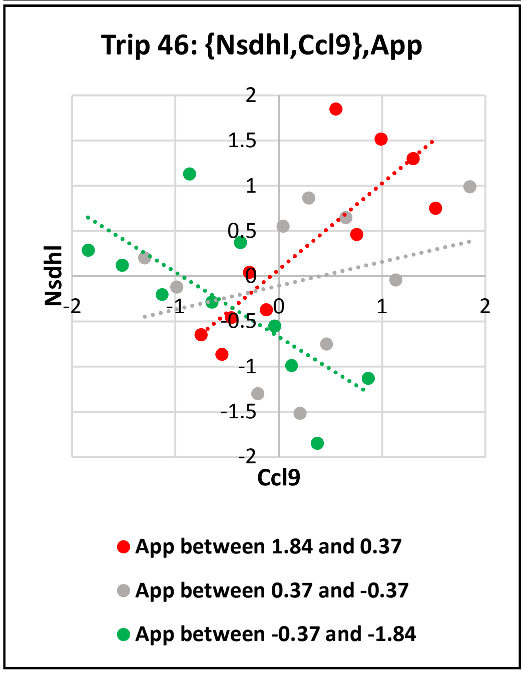

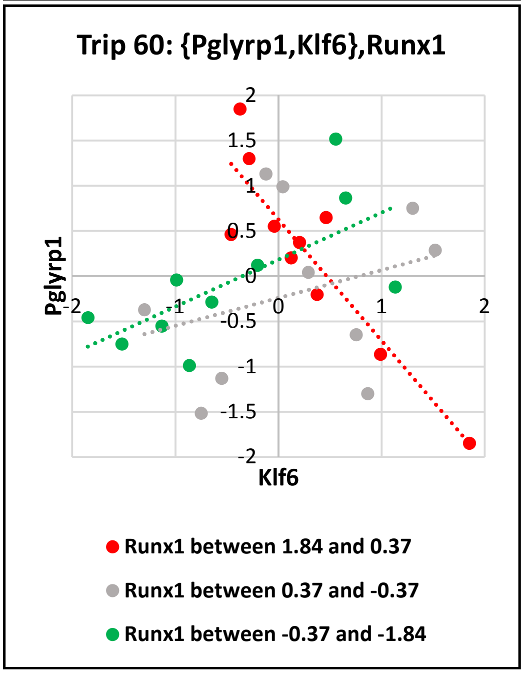

S4 Figs. Scatter plots of 12 triplets in which X_1 and X_2 are involved in the same biological process.

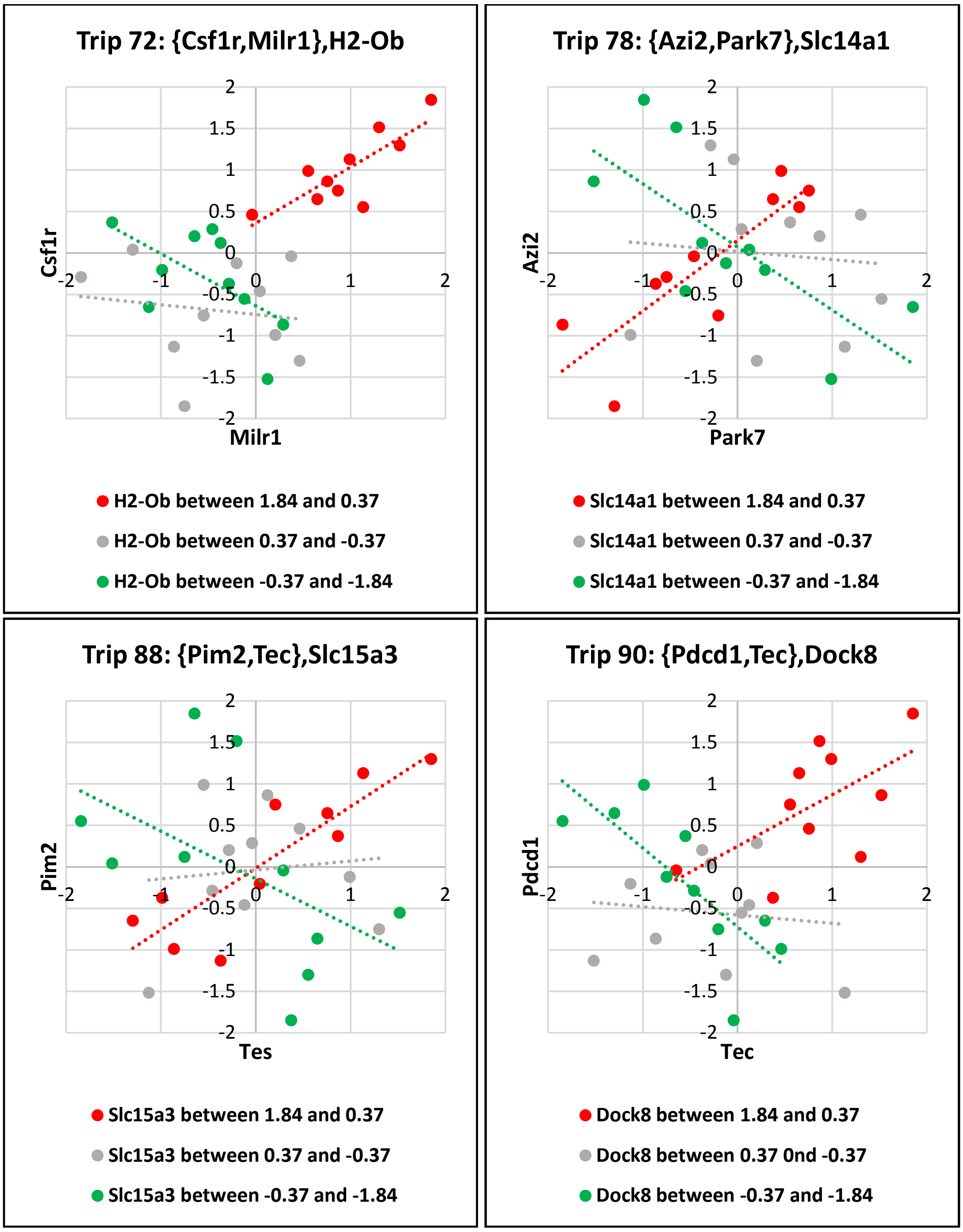

Supplement: S4 Fig — (PDF) [file pone.0184697.s009.pdf]
