# Supplementary material for: Three-way interaction model to trace the mechanisms involved in Alzheimer’s disease transgenic mice
Source: PLoS One. 2017 Sep 21;12(9):e0184697. doi: 10.1371/journal.pone.0184697 (PMC5608283; doi:10.1371/journal.pone.0184697)
Supplement: S1 Text — (PDF) [file pone.0184697.s010.pdf]

S1 Text: A-The reasons for choosing the mouse rather than human dataset. B- Comparison of this study results with the Lopez-Gonzalez et al. (2015) study.

S1 Text-A: The reasons for choosing the mouse rather than human dataset.

The reasons for choosing the mouse rather than human dataset are mentioned below:

Firstly, to the best of our knowledge, all of the microarray datasets for human Alzheimer's disease (AD) include merely "postmortem" samples. Note that in such datasets, the environmental effects and the medical history of the patients are not generally controlled. Furthermore, death is known to be an intervening factor *per se* to gene expression data (Tomita *et al.*, 2004; Franz *et al.*, 2005). Therefore, the postmortem data might not be the best data for our analysis. In contrast, we used mouse gene expression data to minimize the intervening factors.

Secondly, to study the switching mechanisms which involve in the trigger of Alzheimer's disease, we need microarray datasets that include a reasonably large set of microarray data of early stage AD. The E-MTAB-2121 dataset includes 15 samples of early stage AD and 17 controls

In conclusion, if there had been a human dataset including a large-enough dataset of early stage AD under controlled environmental and medical conditions, we would have certainly expected a reasonable similarity between the results of such a human dataset and our mouse dataset.

S1 Text: A-The reasons for choosing the mouse rather than human dataset. B- Comparison of this study results with the Lopez-Gonzalez et al. (2015) study.

S1 Text-B: Comparison of this study results with the Lopez-Gonzalez et al. (2015) study.

Lopez-Gonzalez et al. (2015) mentioned that they have observed 359 genes which are differentially expressed between APP/PS1 and wild type mice [Page 9, Paragraph 1]. Unfortunately, this list is not provided by the authors along with the published paper. More specifically, they found that in three, six and twelve months old mice, 2, 113 and 332 genes are differentially expressed, respectively (when APP/PS1 and wild type mice are compared). It should be noted that 88 genes are in common between the second and the third lists. In other words, the complete list of differentially expressed genes include 2+113+332 genes minus the 88 shared genes = 359 genes. From this list, Lopez-Gonzalez et al. chose 22 of the most significant genes, which are involved in inflammation [Page 9, Column 2, Paragraph 2], for further analysis. The list of these 22 genes are reported in their paper, together with the two other genes, namely App and Prnp, which are differentially expressed when three months old APP/PS1 and wild type mice are compared.

In our study, each of the X_3 genes has two characteristics: (i) it must be differentially expressed; and (ii) it must be a switch, i.e., there exists two other genes, say X_1 and X_2, whose correlation depends on the expression level of the X_3 gene. Therefore, the list of X_3 genes (35 genes) must certainly be a subset of the list of all 359 differentially expressed genes.

As discussed above, Lopez-Gonzalez et al. (2015), have only mentioned the IDs of 22 differentially expressed genes. Nevertheless, we found that 4 of these genes, namely Csf3r, Tlr4, Ccl3, and Ccl4 are included in the list of 22 most significant genes. Interestingly, the only two differentially expressed genes in the three months old mice, namely App and Prnp, are also found to be X_3 genes in our study, which suggest the important role of these genes in triggering the disease. [Lopez-Gonzalez et al. (2015), Page 9, Paragraph 1]. All of these 6 common genes are included in Supplementary files S4 and also S5.

S1 Text: A-The reasons for choosing the mouse rather than human dataset. B- Comparison of this study results with the Lopez-Gonzalez et al. (2015) study.

References:

Franz, H., Ullmann, C., Becker, A., Ryan, M., Bahn, S., Arendt, T., Simon, M., Pääbo, S. & Khaitovich, P. (2005) Systematic analysis of gene expression in human brains before and after death. *Genome biology*, **6**, R112.

Tomita, H., Vawter, M.P., Walsh, D.M., Evans, S.J., Choudary, P.V., Li, J., Overman, K.M., Atz, M.E., Myers, R.M., Jones, E.G., Watson, S.J., Akil, H. & Bunney, W.E., Jr. (2004) Effect of agonal and postmortem factors on gene expression profile: quality control in microarray analyses of postmortem human brain. *Biological psychiatry*, **55**, 346-352.
