# Supplementary material for: Three-way interaction model to trace the mechanisms involved in Alzheimer’s disease transgenic mice
Source: PLoS One. 2017 Sep 21;12(9):e0184697. doi: 10.1371/journal.pone.0184697 (PMC5608283; doi:10.1371/journal.pone.0184697)
Supplement: S1 File — (PDF) [file pone.0184697.s011.pdf]

S1 File-A. Gene regulatory network of the involved genes in the 106 triplets by considering *p*-value <0.0001 as the threshold. In this figure, the regulatory relationships of significant triplets are shown in red.

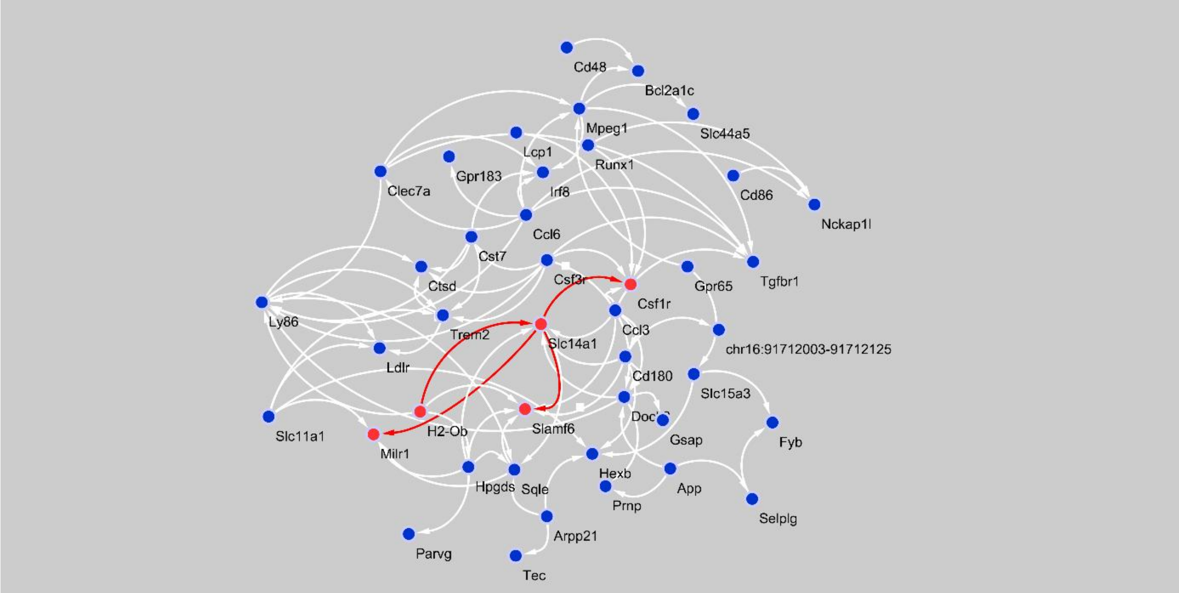

S1 File-B. As follow, the steps of construction gene regulatory network and detection of significant triplets in this network are explained.

Step1- We considered the complete list of genes which are involved in the “significant” triplets. Step2- For this list of genes, the GRN was constructed based on mutual information from pairwise relationships using geWorkbench_2.6.0 software application ARACNE. Step3- We traced the significant triplets in the GRN. For this purpose, we assume that there are 3 expected regulatory relationships between genes involved in a triplet.

1- X_3 regulate X_1
2- X_3 regulate X_2
3- X_3 regulate X_1 and X_3 regulate X_2

To identify the overlap between significant triplets obtained from liquid association method and GRN, we traced the shortest paths between X_3 and X_1 ($X_3 \rightarrow X_1$), also X_3 and X_2 ($X_3 \rightarrow X_2$) for each of the 106 significant triplets in the GRN.
